# Supplementary material for: Blood pressure and cognitive function across the eighth decade: a prospective study of the Lothian Birth Cohort of 1936
Source: BMJ Open. 2020 Jul 23;10(7):e033990. doi: 10.1136/bmjopen-2019-033990 (PMC7380861; doi:10.1136/bmjopen-2019-033990)
Supplement: Supplementary data [file bmjopen-2019-033990supp001.pdf]

Blood pressure and cognitive function across the eighth decade: a prospective study of the Lothian Birth Cohort of 1936

Supplementary Materials

Supplementary Figures 1, 2

Supplementary Tables 1 – 4

Supplementary Figure 1. Simplified path diagram of the hierarchical curve of factors latent growth model of blood pressure change in the LBC1936. Circles represent latent variables and squares represent measured variables. Growth curves, including latent level, linear slope, and quadrative slope factors, were estimated from both systolic and diastolic reading. Basis coefficients (loadings on the individual test slopes) were fixed at 0, 2.96, 6.72, and 9.79 for the linear slops and 0, 8.76, 45.16, and 95.84 for the quadratic slopes to precisely reflect the amount of time passing between assessments. Dashed lines indicate the relationships between covariates which differed between waves and their effects on the individual measurements at different waves. To preserve interpretability, not all variables and paths are shown. Solid, double arrowed lines indicate correlations between latent variables. Dashed lines ending in diamonds represent systolic and diastolic measurements, modelled the same way as at age 70.

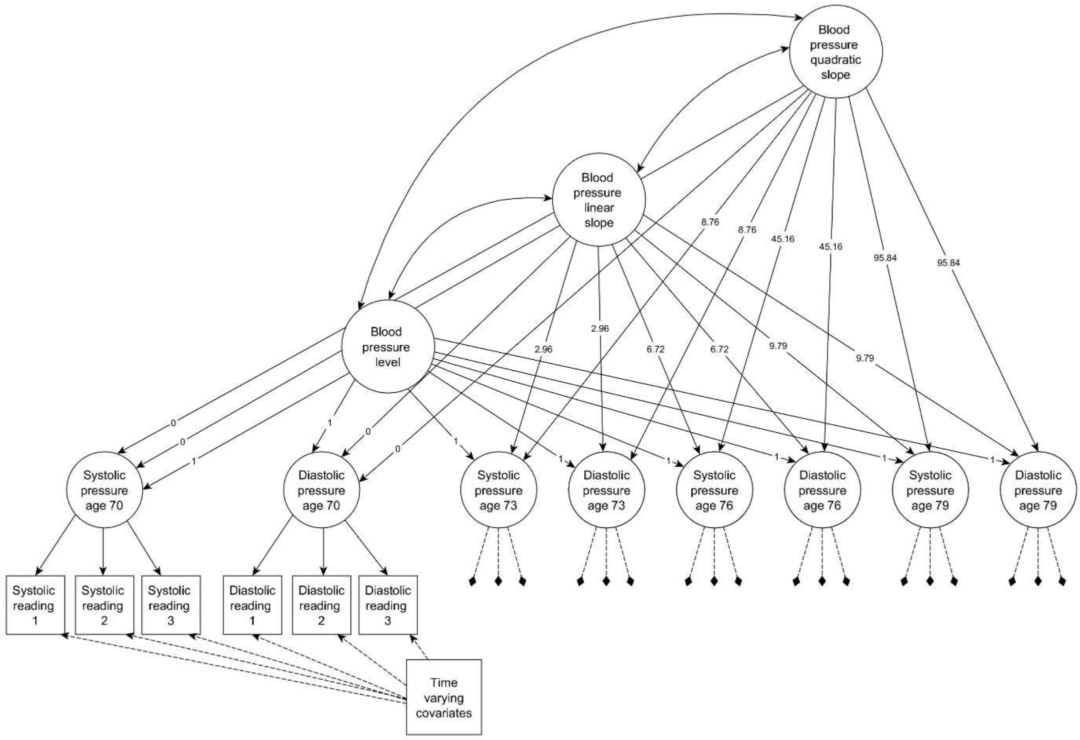

Supplementary Figure 2. Simplified path diagram of the bivariate growth curves of blood pressure and cognitive function, as well as the underlying structure of cognitive function. Circles represent latent variables and squares represent measured variables. Latent variables for blood pressure were modelled as shown in Figure 1. Growth curves, including a latent level and linear slope factor, were estimated for each cognitive test, and the intercepts and slopes were analyzed in a hierarchical model which contained domain and overall factors of both level and slope. Basis coefficients (loadings on the individual test slopes) were fixed at 0, 2.96, 6.72, and 9.79 to precisely represent the amount of time passing between assessments. Dashed lines indicate the relationships between covariates which were fixed across waves and the overall and domain latent variables. To preserve interpretability, not all covariate relationships are shown. Solid, double arrowed lines indicate correlations between latent variables. Although only two domains and four tests are shown here, the full model used all fourteen tests and four domains, as described in the Methods.

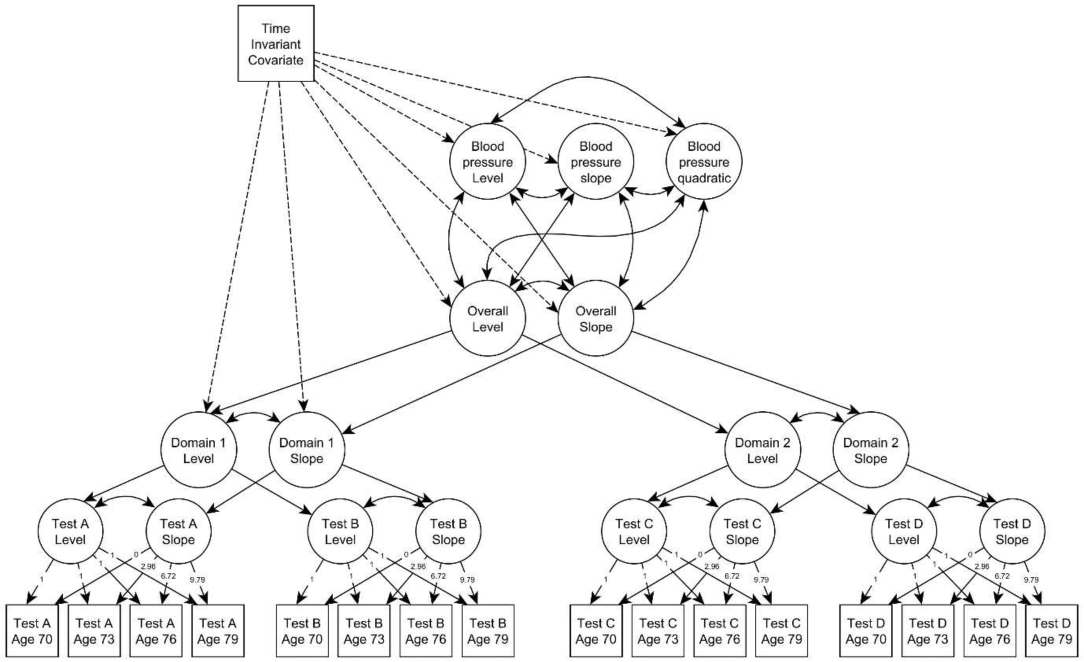

Supplementary Table 1. Descriptive statistics for cognitive, demographic, and clinical variables from study completers only (n = 539).

	Wave 1	Wave 2	Wave 3	Wave 4
Female	267 (49.5%)	-	-	-
Ever had high BP	193 (35.8%)	256 (47.5%)	286 (53.1%)	314 (58.3%)
Ever had CVD	123 (22.8%)	151 (28.0%)	185 (34.3%)	198 (36.7%)
Ever had stroke	18 (3.33%)	31 (5.75%)	55 (10.2%)	69 (12.5%)
Current smoker	24 (4.54%)	22 (4.08%)	19 (3.52%)	21 (3.90%)
Ex-smoker	226 (41.9%)	228 (42.3%)	222 (41.2%)	229 (42.4%)
Average sitting systolic BP	147.7 (17.63)	147.7 (18.18)	147.7 (18.75)	145.1 (18.7)
Average sitting diastolic BP	81.0 (9.53)	77.9 (9.53)	79.0 (10.3)	76.8 (10.1)
Years of education	10.9 (1.17)	-	-	-
Age 11 IQ	102.2 (15.15)	-	-	-
Matrix Reasoning*	14.7 (5.02)	13.2 (4.89)	13.3 (4.91)	13.0 (5.02)
Block Design*	35.7 (10.1)	34.7 (10.1)	32.7 (9.79)	31.3 (9.66)
Spatial Span*	7.27 (1.71)	7.15 (1.58)	7.15 (1.58)	6.75 (1.60)
NART†	35.6 (7.97)	35.2 (7.99)	35.6 (7.95)	35.7 (8.10)
WTAR†	41.9 (6.95)	41.8 (6.61)	41.6 (6.93)	41.7 (6.97)
Verbal Fluency†	43.6 (12.8)	44.4 (13.0)	43.7 (12.7)	43.7 (13.3)
Logical Memory‡	74.4 (16.9)	76.3 (16.9)	75.8 (18.4)	73.0 (20.3)
VPA‡	27.9 (8.53)	28.4 (9.04)	27.3 (9.25)	27.3 (9.44)
Digit Span‡	8.03 (2.37)	8.01 (2.32)	7.91 (2.41)	7.58 (2.19)
LNS‡	11.4 (3.11)	11.4 (3.08)	10.7 (2.98)	10.1 (2.87)
Symbol Search§	25.6 (6.58)	25.5 (5.92)	25.3 (6.26)	22.8 (6.64)
Digit-Symbol Coding§	59.0 (12.4)	58.4 (12.0)	55.4 (12.3)	51.4 (13.0)
Inspection Time§	113 (10.5)	112 (11.7)	111 (11.8)	107 (13.6)
Reaction Time§	0.63 (0.08)	0.64 (0.08)	0.67 (0.09)	0.70 (0.11)
MMSE	28.9 (1.28)	28.9 (1.21)	28.8 (1.53)	28.5 (2.16)

CVD, cardiovascular disease; BP, blood pressure; NART, National Adult Reading Test; WTAR, Wechsler Test of Adult Reading; VPA, Visual Paired Associates; LNS, Letter-Number Sequencing; MMSE, Mini-Mental State Examination; T2D, individuals with type 2 diabetes; T2D was defined as self-reported physician diagnosis of diabetes. * part of the visuospatial ability domain, † part of the crystallized ability domain, ‡ part of the memory domain, § part of the processing speed domain.

Supplementary Table 2. Bivariate associations of the latent growth curve model (1A) of blood pressure and cognitive function.

LHS	Path	RHS	Std. Estimate	S.E.
Blood pressure level	~~	Blood pressure quadratic	0.293	0.065
Blood pressure slope	~~	Blood pressure quadratic	-0.926	0.017
Blood pressure level	~~	Blood pressure slope	-0.424	0.052
Cognitive function level	~~	Cognitive function slope	0.326	0.059
Fluid function level	~~	Fluid function slope	-0.570	1.150
Processing speed level	~~	Processing speed slope	-0.255	0.123
Memory ability level	~~	Memory ability slope	-0.394	0.108
Crystallized ability level	~~	Crystallized ability slope	-0.277	0.377
Blood pressure level	~~	Cognitive function level	-0.024	0.036
Blood pressure slope	~~	Cognitive function slope	-0.097	0.077
Blood pressure quadratic	~~	Cognitive function slope	0.171	0.076
Blood pressure level	~~	Cognitive function slope	0.046	0.043
Blood pressure slope	~~	Cognitive function level	-0.003	0.055
Blood pressure quadratic	~~	Cognitive function level	0.015	0.069
Cognitive function slope	~	Sex	0.110	0.043
Cognitive function slope	~	Age 11 cognitive function	0.016	0.044
Cognitive function slope	~	Education	-0.069	0.049
Cognitive function slope	~	Sex x age 11 CF	-0.078	0.054
Blood pressure quadratic	~	Sex	0.102	0.054
Blood pressure quadratic	~	Age 11 cognitive function	0.023	0.095
Blood pressure quadratic	~	Education	0.060	0.060
Blood pressure quadratic	~	Sex x age 11 CF	-0.122	0.085
Blood pressure slope	~	Sex	-0.020	0.048
Blood pressure slope	~	Age 11 cognitive function	-0.031	0.079
Blood pressure slope	~	Education	-0.058	0.059
Blood pressure slope	~	Sex x age 11 CF	0.116	0.074

Blood pressure level	~	Sex	-0.059	0.032
Blood pressure level	~	Age 11 cognitive function	0.079	0.049
Blood pressure level	~	Education	-0.024	0.038
Blood pressure level	~	Sex x age 11 CF	-0.136	0.050
Cognitive function level	~	Sex x age 11 CF	0.022	0.029
Cognitive function level	~	Sex	-0.050	0.026
Cognitive function level	~	Age 11 cognitive function	0.694	0.037
Cognitive function level	~	Education	0.222	0.019
Systolic pressure measurement 1, wave 1	~	Age at wave 1	-0.010	0.025
Systolic pressure measurement 2, wave 1	~	Age at wave 1	-0.040	0.024
Systolic pressure measurement 3, wave 1	~	Age at wave 1	-0.057	0.028
Diastolic pressure measurement 1, wave 1	~	Age at wave 1	-0.074	0.025
Diastolic pressure measurement 2, wave 1	~	Age at wave 1	-0.061	0.027
Diastolic pressure measurement 3, wave 1	~	Age at wave 1	-0.089	0.028
Systolic pressure measurement 1, wave 2	~	Age at wave 2	-0.029	0.030
Systolic pressure measurement 2, wave 2	~	Age at wave 2	0.001	0.029
Systolic pressure measurement 3, wave 2	~	Age at wave 2	0.010	0.033
Diastolic pressure measurement 1, wave 2	~	Age at wave 2	0.019	0.039
Diastolic pressure measurement 2, wave 2	~	Age at wave 2	0.020	0.035
Diastolic pressure measurement 3, wave 2	~	Age at wave 2	0.014	0.033
Systolic pressure measurement 1, wave 3	~	Age at wave 3	-0.069	0.036
Systolic pressure measurement 2, wave 3	~	Age at wave 3	-0.092	0.032
Systolic pressure measurement 3, wave 3	~	Age at wave 3	-0.081	0.034
Diastolic pressure measurement 1, wave 3	~	Age at wave 3	0.026	0.038
Diastolic pressure measurement 2, wave 3	~	Age at wave 3	0.028	0.036
Diastolic pressure measurement 3, wave 3	~	Age at wave 3	0.014	0.039
Systolic pressure measurement 1, wave 4	~	Age at wave 4	-0.043	0.032
Systolic pressure measurement 2, wave 4	~	Age at wave 4	-0.077	0.027
Systolic pressure measurement 3, wave 4	~	Age at wave 4	-0.051	0.028
Diastolic pressure measurement 1, wave 4	~	Age at wave 4	0.049	0.042
Diastolic pressure measurement 2, wave 4	~	Age at wave 4	0.039	0.030

Diastolic pressure measurement 3, wave 4	~	Age at wave 4	0.017	0.026
Systolic pressure measurement 1, wave 1	~~	Systolic pressure measurement 1, wave 1	0.131	0.010
Systolic pressure measurement 2, wave 1	~~	Systolic pressure measurement 2, wave 1	0.074	0.008
Systolic pressure measurement 3, wave 1	~~	Systolic pressure measurement 3, wave 1	0.097	0.008
Diastolic pressure measurement 1, wave 1	~~	Diastolic pressure measurement 1, wave 1	0.141	0.014
Diastolic pressure measurement 2, wave 1	~~	Diastolic pressure measurement 2, wave 1	0.093	0.014
Diastolic pressure measurement 3, wave 1	~~	Diastolic pressure measurement 3, wave 1	0.150	0.015
Systolic pressure measurement 1, wave 2	~~	Systolic pressure measurement 1, wave 2	0.247	0.018
Systolic pressure measurement 2, wave 2	~~	Systolic pressure measurement 2, wave 2	0.039	0.008
Systolic pressure measurement 3, wave 2	~~	Systolic pressure measurement 3, wave 2	0.069	0.007
Diastolic pressure measurement 1, wave 2	~~	Diastolic pressure measurement 1, wave 2	0.113	0.013
Diastolic pressure measurement 2, wave 2	~~	Diastolic pressure measurement 2, wave 2	0.082	0.011
Diastolic pressure measurement 3, wave 2	~~	Diastolic pressure measurement 3, wave 2	0.094	0.016
Systolic pressure measurement 1, wave 3	~~	Systolic pressure measurement 1, wave 3	0.155	0.015
Systolic pressure measurement 2, wave 3	~~	Systolic pressure measurement 2, wave 3	0.068	0.013
Systolic pressure measurement 3, wave 3	~~	Systolic pressure measurement 3, wave 3	0.085	0.013
Diastolic pressure measurement 1, wave 3	~~	Diastolic pressure measurement 1, wave 3	0.144	0.021
Diastolic pressure measurement 2, wave 3	~~	Diastolic pressure measurement 2, wave 3	0.137	0.029
Diastolic pressure measurement 3, wave 3	~~	Diastolic pressure measurement 3, wave 3	0.168	0.016
Systolic pressure measurement 1, wave 4	~~	Systolic pressure measurement 1, wave 4	0.113	0.014
Systolic pressure measurement 2, wave 4	~~	Systolic pressure measurement 2, wave 4	0.045	0.009
Systolic pressure measurement 3, wave 4	~~	Systolic pressure measurement 3, wave 4	0.093	0.012
Diastolic pressure measurement 1, wave 4	~~	Diastolic pressure measurement 1, wave 4	0.140	0.025
Diastolic pressure measurement 2, wave 4	~~	Diastolic pressure measurement 2, wave 4	0.093	0.015
Diastolic pressure measurement 3, wave 4	~~	Diastolic pressure measurement 3, wave 4	0.102	0.011
Blood pressure level	~~	Blood pressure level	0.985	0.008
Blood pressure slope	~~	Blood pressure slope	0.989	0.013
Blood pressure quadratic	~~	Blood pressure quadratic	0.979	0.019
Fluid function level	~~	Fluid function level	0.389	0.045
Processing speed level	~~	Processing speed level	0.449	0.044
Memory ability level	~~	Memory ability level	0.258	0.034

Crystallized ability level	~~	Crystallized ability level	0.343	0.023
Cognitive function level	~~	Cognitive function level	0.314	0.028
Fluid function slope	~~	Fluid function slope	0.053	0.241
Processing speed slope	~~	Processing speed slope	0.177	0.089
Memory ability slope	~~	Memory ability slope	0.369	0.065
Crystallized ability slope	~~	Crystallized ability slope	0.311	0.655
Cognitive function slope	~~	Cognitive function slope	0.977	0.012
Sex	~~	Sex	1.000	0.000
Sex	~~	Age 11 cognitive function	0.065	0.000
Sex	~~	Education	-0.008	0.000
Sex	~~	Sex x age 11 CF	0.051	0.000
Sex	~~	Age at wave 1	0.004	0.000
Sex	~~	Age at wave 2	0.022	0.000
Sex	~~	Age at wave 3	-0.009	0.000
Sex	~~	Age at wave 4	0.001	0.000
Age 11 cognitive function	~~	Age 11 cognitive function	1.000	0.000
Age 11 cognitive function	~~	Education	0.432	0.000
Age 11 cognitive function	~~	Sex x age 11 CF	0.660	0.000
Age 11 cognitive function	~~	Age at wave 1	-0.097	0.000
Age 11 cognitive function	~~	Age at wave 2	-0.084	0.000
Age 11 cognitive function	~~	Age at wave 3	-0.103	0.000
Age 11 cognitive function	~~	Age at wave 4	-0.095	0.000
Education	~~	Education	1.000	0.000
Education	~~	Sex x age 11 CF	0.296	0.000
Education	~~	Age at wave 1	-0.075	0.000
Education	~~	Age at wave 2	-0.047	0.000
Education	~~	Age at wave 3	-0.085	0.000
Education	~~	Age at wave 4	-0.066	0.000
Sex x age 11 CF	~~	Sex x age 11 CF	1.000	0.000
Sex x age 11 CF	~~	Age at wave 1	-0.073	0.000
Sex x age 11 CF	~~	Age at wave 2	-0.065	0.000

Sex x age 11 CF	~~	Age at wave 3	-0.098	0.000
Sex x age 11 CF	~~	Age at wave 4	-0.086	0.000
Age at wave 1	~~	Age at wave 1	1.000	0.000
Age at wave 1	~~	Age at wave 2	0.945	0.000
Age at wave 1	~~	Age at wave 3	0.936	0.000
Age at wave 1	~~	Age at wave 4	0.893	0.000
Age at wave 2	~~	Age at wave 2	1.000	0.000
Age at wave 2	~~	Age at wave 3	0.922	0.000
Age at wave 2	~~	Age at wave 4	0.880	0.000
Age at wave 3	~~	Age at wave 3	1.000	0.000
Age at wave 3	~~	Age at wave 4	0.912	0.000
Age at wave 4	~~	Age at wave 4	1.000	0.000

LHS = Left-hand side (of the structural equation); RHS = right-hand side. The path column indicates the type of relationship between the two variables, ~ indicates a regression, and ~~ indicates a correlation or covariance.

Supplementary Table 3. Bivariate associations of the latent growth curve model (1B) of blood pressure and cognitive function with control covariates.

LHS	Path	RHS	Std. Estimate	S.E.
Blood pressure level	~~	Blood pressure quadratic	0.264	0.095
Blood pressure slope	~~	Blood pressure quadratic	-0.929	0.018
Blood pressure level	~~	Blood pressure slope	-0.385	0.077
Cognitive function level	~~	Cognitive function slope	0.358	0.095
Fluid function level	~~	Fluid function slope	-0.682	0.504
Processing speed level	~~	Processing speed slope	-0.395	0.123
Memory ability level	~~	Memory ability slope	-0.455	0.092
Crystallized ability level	~~	Crystallized ability slope	-0.174	0.330
Blood pressure level	~~	Cognitive function level	-0.046	0.059
Blood pressure slope	~~	Cognitive function slope	-0.110	0.081
Blood pressure quadratic	~~	Cognitive function slope	0.168	0.092
Blood pressure level	~~	Cognitive function slope	0.063	0.057
Blood pressure slope	~~	Cognitive function level	0.038	0.090
Blood pressure quadratic	~~	Cognitive function level	-0.005	0.092
Cognitive function slope	~	Age 11 cognitive function	-0.011	0.088
Cognitive function slope	~	Education	-0.081	0.064
Cognitive function slope	~	APOEe4	-0.183	0.064
Cognitive function slope	~	Sex	0.111	0.055
Cognitive function slope	~	Sex x age 11 CF	0.027	0.066
Blood pressure quadratic	~	Age 11 cognitive function	-0.043	0.112
Blood pressure quadratic	~	Education	0.156	0.088
Blood pressure quadratic	~	Current smoker	-0.194	0.100
Blood pressure quadratic	~	Former Current smoker	-0.012	0.075

Blood pressure quadratic	~	Sex	0.171	0.061
Blood pressure quadratic	~	Sex x age 11 CF	-0.035	0.110
Blood pressure slope	~	Age 11 cognitive function	0.002	0.099
Blood pressure slope	~	Education	-0.140	0.075
Blood pressure slope	~	Current smoker	0.178	0.089
Blood pressure slope	~	Former Current smoker	0.014	0.072
Blood pressure slope	~	Sex	-0.073	0.049
Blood pressure slope	~	Sex x age 11 CF	0.054	0.102
Blood pressure level	~	Age 11 cognitive function	0.099	0.072
Blood pressure level	~	Education	-0.029	0.053
Blood pressure level	~	Current smoker	-0.104	0.040
Blood pressure level	~	Former Current smoker	0.011	0.047
Blood pressure level	~	Sex	-0.055	0.046
Blood pressure level	~	Sex x age 11 CF	-0.106	0.058
Cognitive function level	~	Age 11 cognitive function	0.741	0.035
Cognitive function level	~	Education	0.242	0.031
Cognitive function level	~	APOEe4	-0.083	0.027
Cognitive function level	~	Sex	-0.051	0.025
Cognitive function level	~	Sex x age 11 CF	0.011	0.051
Systolic pressure measurement 1, wave 1	~	Age at wave 1	-0.050	0.047
Systolic pressure measurement 1, wave 1	~	CVD history at wave 1	0.011	0.038
Systolic pressure measurement 1, wave 1	~	Stroke history at wave 1	0.037	0.047
Systolic pressure measurement 2, wave 1	~	Age at wave 1	-0.097	0.040
Systolic pressure measurement 2, wave 1	~	CVD history at wave 1	0.005	0.039
Systolic pressure measurement 2, wave 1	~	Stroke history at wave 1	0.063	0.049
Systolic pressure measurement 3, wave 1	~	Age at wave 1	-0.096	0.048
Systolic pressure measurement 3, wave 1	~	CVD history at wave 1	0.012	0.037
Systolic pressure measurement 3, wave 1	~	Stroke history at wave 1	0.061	0.048
Diastolic pressure measurement 1, wave 1	~	Age at wave 1	-0.103	0.038
Diastolic pressure measurement 1, wave 1	~	CVD history at wave 1	-0.050	0.045

Diastolic pressure measurement 1, wave 1	~	Stroke history at wave 1	0.028	0.029
Diastolic pressure measurement 2, wave 1	~	Age at wave 1	-0.079	0.044
Diastolic pressure measurement 2, wave 1	~	CVD history at wave 1	-0.012	0.045
Diastolic pressure measurement 2, wave 1	~	Stroke history at wave 1	0.030	0.028
Diastolic pressure measurement 3, wave 1	~	Age at wave 1	-0.102	0.037
Diastolic pressure measurement 3, wave 1	~	CVD history at wave 1	-0.041	0.044
Diastolic pressure measurement 3, wave 1	~	Stroke history at wave 1	0.035	0.027
Systolic pressure measurement 1, wave 2	~	Age at wave 2	-0.024	0.043
Systolic pressure measurement 1, wave 2	~	CVD history at wave 2	-0.122	0.037
Systolic pressure measurement 1, wave 2	~	Stroke history at wave 2	0.025	0.042
Systolic pressure measurement 2, wave 2	~	Age at wave 2	0.011	0.049
Systolic pressure measurement 2, wave 2	~	CVD history at wave 2	-0.045	0.035
Systolic pressure measurement 2, wave 2	~	Stroke history at wave 2	-0.014	0.045
Systolic pressure measurement 3, wave 2	~	Age at wave 2	0.026	0.046
Systolic pressure measurement 3, wave 2	~	CVD history at wave 2	-0.026	0.031
Systolic pressure measurement 3, wave 2	~	Stroke history at wave 2	-0.011	0.042
Diastolic pressure measurement 1, wave 2	~	Age at wave 2	0.048	0.043
Diastolic pressure measurement 1, wave 2	~	CVD history at wave 2	-0.053	0.053
Diastolic pressure measurement 1, wave 2	~	Stroke history at wave 2	-0.017	0.037
Diastolic pressure measurement 2, wave 2	~	Age at wave 2	0.053	0.041
Diastolic pressure measurement 2, wave 2	~	CVD history at wave 2	-0.051	0.051
Diastolic pressure measurement 2, wave 2	~	Stroke history at wave 2	-0.019	0.035
Diastolic pressure measurement 3, wave 2	~	Age at wave 2	0.042	0.042
Diastolic pressure measurement 3, wave 2	~	CVD history at wave 2	-0.037	0.053
Diastolic pressure measurement 3, wave 2	~	Stroke history at wave 2	-0.014	0.038
Systolic pressure measurement 1, wave 3	~	Age at wave 3	-0.101	0.048
Systolic pressure measurement 1, wave 3	~	CVD history at wave 3	-0.108	0.037
Systolic pressure measurement 1, wave 3	~	Stroke history at wave 3	-0.078	0.040
Systolic pressure measurement 2, wave 3	~	Age at wave 3	-0.092	0.048
Systolic pressure measurement 2, wave 3	~	CVD history at wave 3	-0.116	0.035

Systolic pressure measurement 2, wave 3	~	Stroke history at wave 3	-0.045	0.041
Systolic pressure measurement 3, wave 3	~	Age at wave 3	-0.103	0.049
Systolic pressure measurement 3, wave 3	~	CVD history at wave 3	-0.083	0.038
Systolic pressure measurement 3, wave 3	~	Stroke history at wave 3	-0.028	0.041
Diastolic pressure measurement 1, wave 3	~	Age at wave 3	0.043	0.041
Diastolic pressure measurement 1, wave 3	~	CVD history at wave 3	-0.055	0.056
Diastolic pressure measurement 1, wave 3	~	Stroke history at wave 3	-0.047	0.046
Diastolic pressure measurement 2, wave 3	~	Age at wave 3	0.060	0.043
Diastolic pressure measurement 2, wave 3	~	CVD history at wave 3	-0.033	0.042
Diastolic pressure measurement 2, wave 3	~	Stroke history at wave 3	-0.044	0.045
Diastolic pressure measurement 3, wave 3	~	Age at wave 3	0.026	0.044
Diastolic pressure measurement 3, wave 3	~	CVD history at wave 3	-0.049	0.053
Diastolic pressure measurement 3, wave 3	~	Stroke history at wave 3	-0.046	0.045
Systolic pressure measurement 1, wave 4	~	Age at wave 4	-0.062	0.031
Systolic pressure measurement 1, wave 4	~	CVD history at wave 4	0.013	0.034
Systolic pressure measurement 1, wave 4	~	Stroke history at wave 4	-0.082	0.037
Systolic pressure measurement 2, wave 4	~	Age at wave 4	-0.092	0.031
Systolic pressure measurement 2, wave 4	~	CVD history at wave 4	-0.008	0.034
Systolic pressure measurement 2, wave 4	~	Stroke history at wave 4	-0.029	0.040
Systolic pressure measurement 3, wave 4	~	Age at wave 4	-0.068	0.036
Systolic pressure measurement 3, wave 4	~	CVD history at wave 4	0.004	0.037
Systolic pressure measurement 3, wave 4	~	Stroke history at wave 4	-0.058	0.044
Diastolic pressure measurement 1, wave 4	~	Age at wave 4	0.056	0.046
Diastolic pressure measurement 1, wave 4	~	CVD history at wave 4	-0.061	0.038
Diastolic pressure measurement 1, wave 4	~	Stroke history at wave 4	-0.058	0.045
Diastolic pressure measurement 2, wave 4	~	Age at wave 4	0.039	0.043
Diastolic pressure measurement 2, wave 4	~	CVD history at wave 4	-0.049	0.042
Diastolic pressure measurement 2, wave 4	~	Stroke history at wave 4	-0.030	0.046
Diastolic pressure measurement 3, wave 4	~	Age at wave 4	0.024	0.045
Diastolic pressure measurement 3, wave 4	~	CVD history at wave 4	-0.042	0.044

Diastolic pressure measurement 3, wave 4	~	Stroke history at wave 4	-0.022	0.049
Systolic pressure measurement 1, wave 1	~~	Systolic pressure measurement 1, wave 1	0.129	0.015
Systolic pressure measurement 2, wave 1	~~	Systolic pressure measurement 2, wave 1	0.093	0.010
Systolic pressure measurement 3, wave 1	~~	Systolic pressure measurement 3, wave 1	0.097	0.013
Diastolic pressure measurement 1, wave 1	~~	Diastolic pressure measurement 1, wave 1	0.135	0.018
Diastolic pressure measurement 2, wave 1	~~	Diastolic pressure measurement 2, wave 1	0.099	0.013
Diastolic pressure measurement 3, wave 1	~~	Diastolic pressure measurement 3, wave 1	0.165	0.019
Systolic pressure measurement 1, wave 2	~~	Systolic pressure measurement 1, wave 2	0.197	0.016
Systolic pressure measurement 2, wave 2	~~	Systolic pressure measurement 2, wave 2	0.039	0.008
Systolic pressure measurement 3, wave 2	~~	Systolic pressure measurement 3, wave 2	0.082	0.009
Diastolic pressure measurement 1, wave 2	~~	Diastolic pressure measurement 1, wave 2	0.100	0.015
Diastolic pressure measurement 2, wave 2	~~	Diastolic pressure measurement 2, wave 2	0.075	0.011
Diastolic pressure measurement 3, wave 2	~~	Diastolic pressure measurement 3, wave 2	0.110	0.021
Systolic pressure measurement 1, wave 3	~~	Systolic pressure measurement 1, wave 3	0.141	0.018
Systolic pressure measurement 2, wave 3	~~	Systolic pressure measurement 2, wave 3	0.083	0.010
Systolic pressure measurement 3, wave 3	~~	Systolic pressure measurement 3, wave 3	0.085	0.012
Diastolic pressure measurement 1, wave 3	~~	Diastolic pressure measurement 1, wave 3	0.135	0.022
Diastolic pressure measurement 2, wave 3	~~	Diastolic pressure measurement 2, wave 3	0.146	0.034
Diastolic pressure measurement 3, wave 3	~~	Diastolic pressure measurement 3, wave 3	0.172	0.025
Systolic pressure measurement 1, wave 4	~~	Systolic pressure measurement 1, wave 4	0.118	0.013
Systolic pressure measurement 2, wave 4	~~	Systolic pressure measurement 2, wave 4	0.042	0.007
Systolic pressure measurement 3, wave 4	~~	Systolic pressure measurement 3, wave 4	0.104	0.013
Diastolic pressure measurement 1, wave 4	~~	Diastolic pressure measurement 1, wave 4	0.148	0.023
Diastolic pressure measurement 2, wave 4	~~	Diastolic pressure measurement 2, wave 4	0.102	0.018
Diastolic pressure measurement 3, wave 4	~~	Diastolic pressure measurement 3, wave 4	0.098	0.015
Blood pressure level	~~	Blood pressure level	0.976	0.015
Blood pressure slope	~~	Blood pressure slope	0.950	0.035
Blood pressure quadratic	~~	Blood pressure quadratic	0.922	0.045
Fluid function level	~~	Fluid function level	0.489	0.052
Processing speed level	~~	Processing speed level	0.545	0.051

Memory ability level	~~	Memory ability level	0.350	0.045
Crystallized ability level	~~	Crystallized ability level	0.325	0.037
Cognitive function level	~~	Cognitive function level	0.212	0.031
Fluid function slope	~~	Fluid function slope	0.158	0.249
Processing speed slope	~~	Processing speed slope	0.239	0.100
Memory ability slope	~~	Memory ability slope	0.409	0.104
Crystallized ability slope	~~	Crystallized ability slope	0.361	0.916
Cognitive function slope	~~	Cognitive function slope	0.944	0.026
Age 11 cognitive function	~~	Age 11 cognitive function	1.000	0.000
Age 11 cognitive function	~~	Education	0.445	0.000
Age 11 cognitive function	~~	APOEe4	-0.043	0.000
Age 11 cognitive function	~~	Sex	0.091	0.000
Age 11 cognitive function	~~	Sex x age 11 CF	0.659	0.000
Age 11 cognitive function	~~	Current smoker	0.012	0.000
Age 11 cognitive function	~~	Former Current smoker	-0.092	0.000
Age 11 cognitive function	~~	Age at wave 1	-0.090	0.000
Age 11 cognitive function	~~	CVD history at wave 1	-0.128	0.000
Age 11 cognitive function	~~	Stroke history at wave 1	0.001	0.000
Age 11 cognitive function	~~	Age at wave 2	-0.076	0.000
Age 11 cognitive function	~~	CVD history at wave 2	-0.097	0.000
Age 11 cognitive function	~~	Stroke history at wave 2	0.039	0.000
Age 11 cognitive function	~~	Age at wave 3	-0.081	0.000
Age 11 cognitive function	~~	CVD history at wave 3	-0.055	0.000
Age 11 cognitive function	~~	Stroke history at wave 3	0.019	0.000
Age 11 cognitive function	~~	Age at wave 4	-0.073	0.000
Age 11 cognitive function	~~	CVD history at wave 4	-0.043	0.000
Age 11 cognitive function	~~	Stroke history at wave 4	-0.027	0.000
Education	~~	Education	1.000	0.000
Education	~~	APOEe4	-0.028	0.000
Education	~~	Sex	0.025	0.000

Education	~~	Sex x age 11 CF	0.263	0.000
Education	~~	Current smoker	0.046	0.000
Education	~~	Former Current smoker	-0.091	0.000
Education	~~	Age at wave 1	-0.088	0.000
Education	~~	CVD history at wave 1	-0.056	0.000
Education	~~	Stroke history at wave 1	-0.035	0.000
Education	~~	Age at wave 2	-0.054	0.000
Education	~~	CVD history at wave 2	-0.043	0.000
Education	~~	Stroke history at wave 2	-0.006	0.000
Education	~~	Age at wave 3	-0.098	0.000
Education	~~	CVD history at wave 3	-0.042	0.000
Education	~~	Stroke history at wave 3	0.032	0.000
Education	~~	Age at wave 4	-0.075	0.000
Education	~~	CVD history at wave 4	-0.064	0.000
Education	~~	Stroke history at wave 4	0.033	0.000
APOEe4	~~	APOEe4	1.000	0.000
APOEe4	~~	Sex	-0.104	0.000
APOEe4	~~	Sex x age 11 CF	-0.013	0.000
APOEe4	~~	Current smoker	-0.111	0.000
APOEe4	~~	Former Current smoker	0.009	0.000
APOEe4	~~	Age at wave 1	0.048	0.000
APOEe4	~~	CVD history at wave 1	0.013	0.000
APOEe4	~~	Stroke history at wave 1	-0.047	0.000
APOEe4	~~	Age at wave 2	0.030	0.000
APOEe4	~~	CVD history at wave 2	0.017	0.000
APOEe4	~~	Stroke history at wave 2	-0.044	0.000
APOEe4	~~	Age at wave 3	0.055	0.000
APOEe4	~~	CVD history at wave 3	0.015	0.000
APOEe4	~~	Stroke history at wave 3	0.000	0.000
APOEe4	~~	Age at wave 4	0.033	0.000

APOEe4	~~	CVD history at wave 4	-0.001	0.000
APOEe4	~~	Stroke history at wave 4	0.000	0.000
Sex	~~	Sex	1.000	0.000
Sex	~~	Sex x age 11 CF	0.179	0.000
Sex	~~	Current smoker	0.078	0.000
Sex	~~	Former Current smoker	-0.123	0.000
Sex	~~	Age at wave 1	0.020	0.000
Sex	~~	CVD history at wave 1	-0.122	0.000
Sex	~~	Stroke history at wave 1	-0.021	0.000
Sex	~~	Age at wave 2	0.043	0.000
Sex	~~	CVD history at wave 2	-0.149	0.000
Sex	~~	Stroke history at wave 2	-0.024	0.000
Sex	~~	Age at wave 3	0.003	0.000
Sex	~~	CVD history at wave 3	-0.113	0.000
Sex	~~	Stroke history at wave 3	-0.038	0.000
Sex	~~	Age at wave 4	0.018	0.000
Sex	~~	CVD history at wave 4	-0.084	0.000
Sex	~~	Stroke history at wave 4	-0.047	0.000
Sex x age 11 CF	~~	Sex x age 11 CF	1.000	0.000
Sex x age 11 CF	~~	Current smoker	0.021	0.000
Sex x age 11 CF	~~	Former Current smoker	-0.066	0.000
Sex x age 11 CF	~~	Age at wave 1	-0.046	0.000
Sex x age 11 CF	~~	CVD history at wave 1	-0.104	0.000
Sex x age 11 CF	~~	Stroke history at wave 1	-0.018	0.000
Sex x age 11 CF	~~	Age at wave 2	-0.032	0.000
Sex x age 11 CF	~~	CVD history at wave 2	-0.082	0.000
Sex x age 11 CF	~~	Stroke history at wave 2	0.027	0.000
Sex x age 11 CF	~~	Age at wave 3	-0.068	0.000
Sex x age 11 CF	~~	CVD history at wave 3	-0.054	0.000
Sex x age 11 CF	~~	Stroke history at wave 3	0.016	0.000

Sex x age 11 CF	~~	Age at wave 4	-0.047	0.000
Sex x age 11 CF	~~	CVD history at wave 4	-0.073	0.000
Sex x age 11 CF	~~	Stroke history at wave 4	-0.041	0.000
Current smoker	~~	Current smoker	1.000	0.000
Current smoker	~~	Former Current smoker	-0.176	0.000
Current smoker	~~	Age at wave 1	0.056	0.000
Current smoker	~~	CVD history at wave 1	0.069	0.000
Current smoker	~~	Stroke history at wave 1	0.081	0.000
Current smoker	~~	Age at wave 2	0.049	0.000
Current smoker	~~	CVD history at wave 2	0.085	0.000
Current smoker	~~	Stroke history at wave 2	0.043	0.000
Current smoker	~~	Age at wave 3	0.026	0.000
Current smoker	~~	CVD history at wave 3	0.054	0.000
Current smoker	~~	Stroke history at wave 3	0.006	0.000
Current smoker	~~	Age at wave 4	0.058	0.000
Current smoker	~~	CVD history at wave 4	0.020	0.000
Current smoker	~~	Stroke history at wave 4	0.026	0.000
Former Current smoker	~~	Former Current smoker	1.000	0.000
Former Current smoker	~~	Age at wave 1	0.006	0.000
Former Current smoker	~~	CVD history at wave 1	0.109	0.000
Former Current smoker	~~	Stroke history at wave 1	0.004	0.000
Former Current smoker	~~	Age at wave 2	-0.025	0.000
Former Current smoker	~~	CVD history at wave 2	0.102	0.000
Former Current smoker	~~	Stroke history at wave 2	0.027	0.000
Former Current smoker	~~	Age at wave 3	-0.002	0.000
Former Current smoker	~~	CVD history at wave 3	0.080	0.000
Former Current smoker	~~	Stroke history at wave 3	0.076	0.000
Former Current smoker	~~	Age at wave 4	-0.011	0.000
Former Current smoker	~~	CVD history at wave 4	0.130	0.000
Former Current smoker	~~	Stroke history at wave 4	0.053	0.000

Age at wave 1	~~	Age at wave 1	1.000	0.000
Age at wave 1	~~	CVD history at wave 1	0.111	0.000
Age at wave 1	~~	Stroke history at wave 1	0.148	0.000
Age at wave 1	~~	Age at wave 2	0.949	0.000
Age at wave 1	~~	CVD history at wave 2	0.080	0.000
Age at wave 1	~~	Stroke history at wave 2	0.110	0.000
Age at wave 1	~~	Age at wave 3	0.944	0.000
Age at wave 1	~~	CVD history at wave 3	0.035	0.000
Age at wave 1	~~	Stroke history at wave 3	0.067	0.000
Age at wave 1	~~	Age at wave 4	0.893	0.000
Age at wave 1	~~	CVD history at wave 4	0.098	0.000
Age at wave 1	~~	Stroke history at wave 4	0.024	0.000
CVD history at wave 1	~~	CVD history at wave 1	1.000	0.000
CVD history at wave 1	~~	Stroke history at wave 1	0.010	0.000
CVD history at wave 1	~~	Age at wave 2	0.102	0.000
CVD history at wave 1	~~	CVD history at wave 2	0.779	0.000
CVD history at wave 1	~~	Stroke history at wave 2	-0.002	0.000
CVD history at wave 1	~~	Age at wave 3	0.105	0.000
CVD history at wave 1	~~	CVD history at wave 3	0.635	0.000
CVD history at wave 1	~~	Stroke history at wave 3	0.027	0.000
CVD history at wave 1	~~	Age at wave 4	0.085	0.000
CVD history at wave 1	~~	CVD history at wave 4	0.567	0.000
CVD history at wave 1	~~	Stroke history at wave 4	0.036	0.000
Stroke history at wave 1	~~	Stroke history at wave 1	1.000	0.000
Stroke history at wave 1	~~	Age at wave 2	0.148	0.000
Stroke history at wave 1	~~	CVD history at wave 2	-0.014	0.000
Stroke history at wave 1	~~	Stroke history at wave 2	0.761	0.000
Stroke history at wave 1	~~	Age at wave 3	0.132	0.000
Stroke history at wave 1	~~	CVD history at wave 3	-0.014	0.000
Stroke history at wave 1	~~	Stroke history at wave 3	0.570	0.000

Stroke history at wave 1	~~	Age at wave 4	0.132	0.000
Stroke history at wave 1	~~	CVD history at wave 4	-0.047	0.000
Stroke history at wave 1	~~	Stroke history at wave 4	0.511	0.000
Age at wave 2	~~	Age at wave 2	1.000	0.000
Age at wave 2	~~	CVD history at wave 2	0.056	0.000
Age at wave 2	~~	Stroke history at wave 2	0.111	0.000
Age at wave 2	~~	Age at wave 3	0.923	0.000
Age at wave 2	~~	CVD history at wave 3	0.020	0.000
Age at wave 2	~~	Stroke history at wave 3	0.060	0.000
Age at wave 2	~~	Age at wave 4	0.877	0.000
Age at wave 2	~~	CVD history at wave 4	0.067	0.000
Age at wave 2	~~	Stroke history at wave 4	0.013	0.000
CVD history at wave 2	~~	CVD history at wave 2	1.000	0.000
CVD history at wave 2	~~	Stroke history at wave 2	-0.014	0.000
CVD history at wave 2	~~	Age at wave 3	0.082	0.000
CVD history at wave 2	~~	CVD history at wave 3	0.738	0.000
CVD history at wave 2	~~	Stroke history at wave 3	0.014	0.000
CVD history at wave 2	~~	Age at wave 4	0.053	0.000
CVD history at wave 2	~~	CVD history at wave 4	0.684	0.000
CVD history at wave 2	~~	Stroke history at wave 4	0.000	0.000
Stroke history at wave 2	~~	Stroke history at wave 2	1.000	0.000
Stroke history at wave 2	~~	Age at wave 3	0.107	0.000
Stroke history at wave 2	~~	CVD history at wave 3	-0.027	0.000
Stroke history at wave 2	~~	Stroke history at wave 3	0.719	0.000
Stroke history at wave 2	~~	Age at wave 4	0.104	0.000
Stroke history at wave 2	~~	CVD history at wave 4	-0.039	0.000
Stroke history at wave 2	~~	Stroke history at wave 4	0.643	0.000
Age at wave 3	~~	Age at wave 3	1.000	0.000
Age at wave 3	~~	CVD history at wave 3	0.049	0.000
Age at wave 3	~~	Stroke history at wave 3	0.076	0.000

Age at wave 3	~~	Age at wave 4	0.902	0.000
Age at wave 3	~~	CVD history at wave 4	0.102	0.000
Age at wave 3	~~	Stroke history at wave 4	0.034	0.000
CVD history at wave 3	~~	CVD history at wave 3	1.000	0.000
CVD history at wave 3	~~	Stroke history at wave 3	0.015	0.000
CVD history at wave 3	~~	Age at wave 4	0.032	0.000
CVD history at wave 3	~~	CVD history at wave 4	0.783	0.000
CVD history at wave 3	~~	Stroke history at wave 4	-0.020	0.000
Stroke history at wave 3	~~	Stroke history at wave 3	1.000	0.000
Stroke history at wave 3	~~	Age at wave 4	0.065	0.000
Stroke history at wave 3	~~	CVD history at wave 4	0.012	0.000
Stroke history at wave 3	~~	Stroke history at wave 4	0.807	0.000
Age at wave 4	~~	Age at wave 4	1.000	0.000
Age at wave 4	~~	CVD history at wave 4	0.061	0.000
Age at wave 4	~~	Stroke history at wave 4	0.030	0.000
CVD history at wave 4	~~	CVD history at wave 4	1.000	0.000
CVD history at wave 4	~~	Stroke history at wave 4	-0.039	0.000
Stroke history at wave 4	~~	Stroke history at wave 4	1.000	0.000

LHS = Left-hand side (of the structural equation); RHS = right-hand side. The path column indicates the type of relationship between the two variables, ~ indicates a regression, and ~~ indicates a correlation or covariance.

Supplementary Table 4. Cross-sectional Pearson correlations between cognitive function and blood pressure scores.

Wave	Domain	Adjustment	*r*	95% C.I.	*p*
1	General	None	-0.007	-0.069 — 0.055	0.823
		Points added	-0.048	-0.109 — 0.015	0.133
	Fluid	None	-0.010	-0.072 — 0.053	0.762
		Points added	-0.047	-0.108 — 0.016	0.143
	Speed	None	0.036	-0.026 — 0.098	0.255
		Points added	-0.007	-0.069 — 0.055	0.818
	Memory	None	-0.017	-0.079 — 0.045	0.590
		Points added	-0.055	-0.117 — 0.007	0.083
	Crystallized	None	-0.011	-0.073 — 0.052	0.737
		Points added	-0.043	-0.105 — 0.019	0.173
2	General	None	0.013	-0.056 — 0.082	0.718
		Points added	-0.007	-0.076 — 0.062	0.846
	Fluid	None	-0.004	-0.073 — 0.065	0.901
		Points added	-0.021	-0.090 — 0.048	0.554
	Speed	None	0.024	-0.045 — 0.093	0.494
		Points added	-0.003	-0.072 — 0.065	0.921
	Memory	None	0.014	-0.055 — 0.083	0.696
		Points added	-0.002	-0.071 — 0.067	0.962
	Crystallized	None	0.020	-0.049 — 0.089	0.562
		Points added	0.000	-0.069 — 0.069	0.995
3	General	None	-0.013	-0.092 — 0.067	0.750
		Points added	-0.033	-0.112 — 0.046	0.413
	Fluid	None	-0.001	-0.080 — 0.079	0.982
		Points added	-0.022	-0.101 — 0.058	0.596
	Speed	None	-0.006	-0.086 — 0.073	0.874

		Points added	-0.029	-0.109 — 0.050	0.470
	Memory	None	-0.018	-0.097 — 0.062	0.663
		Points added	-0.037	-0.116 — 0.042	0.360
	Crystallized	None	-0.018	-0.097 — 0.062	0.662
		Points added	-0.026	-0.105 — 0.054	0.530
4	General	None	0.006	-0.088 — 0.101	0.895
		Points added	-0.011	-0.106 — 0.084	0.826
	Fluid	None	-0.005	-0.099 — 0.090	0.925
		Points added	-0.024	-0.118 — 0.071	0.625
	Speed	None	0.007	-0.087 — 0.102	0.878
		Points added	-0.001	-0.096 — 0.094	0.985
	Memory	None	0.022	-0.073 — 0.116	0.655
		Points added	0.007	-0.088 — 0.102	0.887
	Crystal	None	-0.036	-0.131 — 0.059	0.454
		Points added	-0.059	-0.153 — 0.037	0.227
