# Supplementary material for: Blood pressure and cognitive function across the eighth decade: a prospective study of the Lothian Birth Cohort of 1936
Source: BMJ Open. 2020 Jul 23;10(7):e033990. doi: 10.1136/bmjopen-2019-033990 (PMC7380861; doi:10.1136/bmjopen-2019-033990)
Supplement: Author's manuscript [file bmjopen-2019-033990.draft_revisions.pdf]

BMJ Open is committed to open peer review. As part of this commitment we make the peer review history of every article we publish publicly available.

When an article is published we post the peer reviewers' comments and the authors' responses online. We also post the versions of the paper that were used during peer review. These are the versions that the peer review comments apply to.

The versions of the paper that follow are the versions that were submitted during the peer review process. They are not the versions of record or the final published versions. They should not be cited or distributed as the published version of this manuscript.

BMJ Open is an open access journal and the full, final, typeset and author-corrected version of record of the manuscript is available on our site with no access controls, subscription charges or pay-per-view fees (http://bmjopen.bmj.com).

If you have any questions on BMJ Open's open peer review process please email info.bmjopen@bmj.com

BMJ Open

Blood pressure and cognitive function across the eighth decade: a prospective study of the Lothian Birth Cohort of 1936

Journal:	*BMJ Open*
Manuscript ID	bmjopen-2019-033990
Article Type:	Original research
Date Submitted by the Author:	02-Sep-2019
Complete List of Authors:	Altschul, Drew; The University of Edinburgh, Psychology<br>Starr, John; Royal Victoria Hospital,<br>Deary, Ian; University of Edinburgh, Department of Psychology
Keywords:	Hypertension < CARDIOLOGY, EPIDEMIOLOGY, Old age psychiatry < PSYCHIATRY, GERIATRIC MEDICINE

SCHOLARONE™
Manuscripts

I, the Submitting Author has the right to grant and does grant on behalf of all authors of the Work (as defined in the below author licence), an exclusive licence and/or a non-exclusive licence for contributions from authors who are: i) UK Crown employees; ii) where BMJ has agreed a CC-BY licence shall apply, and/or iii) in accordance with the terms applicable for US Federal Government officers or employees acting as part of their official duties; on a worldwide, perpetual, irrevocable, royalty-free basis to BMJ Publishing Group Ltd ("BMJ") its licensees and where the relevant Journal is co-owned by BMJ to the co-owners of the Journal, to publish the Work in this journal and any other BMJ products and to exploit all rights, as set out in our licence.

The Submitting Author accepts and understands that any supply made under these terms is made by BMJ to the Submitting Author unless you are acting as an employee on behalf of your employer or a postgraduate student of an affiliated institution which is paying any applicable article publishing charge ("APC") for Open Access articles. Where the Submitting Author wishes to make the Work available on an Open Access basis (and intends to pay the relevant APC), the terms of reuse of such Open Access shall be governed by a Creative Commons licence – details of these licences and which Creative Commons licence will apply to this Work are set out in our licence referred to above.

Other than as permitted in any relevant BMJ Author's Self Archiving Policies, I confirm this Work has not been accepted for publication elsewhere, is not being considered for publication elsewhere and does not duplicate material already published. I confirm all authors consent to publication of this Work and authorise the granting of this licence.

For peer review only

Blood pressure and cognitive function across the eighth decade: a prospective study of the Lothian Birth Cohort of 1936

Running title: Blood pressure and cognitive function across the eighth decade

Drew M Altschul[1,2,3*], John M Starr[1,4], Ian J Deary[1,3]

1. Department of Psychology, The University of Edinburgh, Edinburgh, UK
2. Mental Health Data Science Scotland
3. Centre for Cognitive Ageing and Cognitive Epidemiology, The University of Edinburgh, Edinburgh, UK
4. Alzheimer Scotland Dementia Research Centre, The University of Edinburgh, Edinburgh, UK

* corresponding author: dmaltschul@gmail.com

Word count: 3985

Data elements: Figures 1 – 3; Tables 1, 2; Supplemental file 1

For peer review only - http://bmjopen.bmj.com/site/about/guidelines.xhtml

Abstract

Objectives

We investigated the associations among blood pressure and cognitive functions across the eighth decade, while accounting for anti-hypertensive medication and lifetime stability in cognitive function.

Design

Prospective cohort study.

Setting

This study used data from the Lothian Birth Cohort 1936 (LBC1936) study, which recruited participants living in the Lothian region of Scotland when aged 70 years, most of whom had completed an intelligence test at age 11 years.

Participants

1091 members of the LBC1936 with assessments of cognitive ability in childhood and older adulthood, and blood pressure measurements in older adulthood.

Primary and secondary outcome measures

Participants were followed up at ages 70, 73, 76, and 79, and latent growth curve models and linear mixed models were used to analyse both cognitive functions and blood pressure as primary outcomes.

Results

Blood pressure followed a quadratic trajectory in the eighth decade: on average blood pressure rose in the first waves and subsequently fell. Intercepts and trajectories were not associated between blood pressure and cognitive functions. Women with higher early-life cognitive function generally had lower blood pressure during the eighth decade. Being

For peer review only

For peer review only - http://bmjopen.bmj.com/site/about/guidelines.xhtml

prescribed anti-hypertensive medication was associated with lower blood pressure, but not with better cognitive function.

Conclusions

Our findings indicate an association between early-life cognitive function and later-life blood pressure, specifically among women. However, we did not find support for the hypothesis that rises in blood pressure and worse cognitive decline are associated with one another in the eighth decade.

Keywords: hypertension, old age psychiatry, geriatric medicine, epidemiology

For peer review only

Strengths and limitations of this study

- This study used direct blood pressure measurements to model a continuous blood pressure score, as well as anti-hypertensive medication data, which were used to adjust blood pressure measurements.
- This study had comprehensive tests of cognitive ability measured in both childhood and old age which allowed us to investigate whether childhood and old age cognitive ability are distinctly related to blood pressure.
- Latent growth curve modelling allowed us to evaluate whether changes in either blood pressure or cognitive functions have downstream associations with one another.
- Larger samples and longer follow-up times, both from earlier and later in life, are needed to understand the long-term relationships among blood pressure, anti-hypertensive treatment, and cognitive functions.

For peer review only

Introduction

Hypertension is a major issue in middle and older age populations.[1 2] Hypertension has been consistently linked to cardiovascular diseases such as coronary artery disease (CAD) and stroke.[1 3] It is also a risk factor for neurocognitive conditions such as age-related cognitive decline, vascular dementia, and possibly Alzheimer's disease.[4] Accelerated cognitive decline is associated with lower well-being, higher morbidity and mortality and, as cognitive function worsens, the clinical conditions of mild cognitive impairment and dementia can develop.[5] Some of hypertension's negative impacts on cognitive function have likely causal pathways: hypertension disrupts cerebral blood vessel structure and function, and is associated with stroke in relevant white matter regions.[6] Worldwide, hypertension, age-related cognitive decline, and dementia are on the rise.[6 7]

A major objective of modern gerontology is to understand how age-related cognitive changes systematically differ between individuals and groups. Typically, hypertension is thought of as a risk factor for later life cognitive decline. In some samples of older people, hypertension is associated with lower cognitive functioning and faster decline.[6 8 9] However, there is also evidence for the relationship operating in the opposite direction, i.e. that higher cognitive function in youth is associated with having lower risk of developing hypertension[10] and experiencing hypertension-related stroke and coronary artery events later in life.[11] These latter findings are part of a field known as cognitive epidemiology, which has found that higher cognitive function in early life is associated with lower risk of a number of physical and mental ailments later in life.[12-16]

Men are more likely to develop cardiovascular conditions than women,[17] a reason why men have been the subject of more intervention studies than women.[18] Nevertheless, cardiovascular disease is the leading cause of death in both women and men.[19] Some differences in hypertension are biologically-based in differences between men and women, e.g. through hormones and gene dosage from the sex chromosomes, and these differences are consistent across different countries and ethnic groups.[18] Additionally, traditional gender

For peer review only - http://bmjopen.bmj.com/site/about/guidelines.xhtml

roles are associated with men behaving in ways (e.g., higher smoking rates) that increase their risk for physical health conditions, including hypertension.[20] Previous work on the cognitive epidemiology of hypertension, CAD, and stroke found significant interactions between sex and cognitive function in youth: individuals with higher cognitive function were at lower risk for hypertension, CAD, and stroke, and the associations were stronger in women.[11 21]

In the present study we tested two hypotheses regarding the relationships between cognitive functions, some of which steadily decline in mean level in older participants, and hypertension, a condition that becomes increasingly common with age and is related to cardiovascular health and cognitive impairment. First, we hypothesized that the association between higher cognitive function at age 11 years and lower blood pressure in the eighth decade of life is stronger in women than it is in men.[11] Second, we tested the hypothesis that cognitive function and blood pressure will be reciprocally associated with each other across the eighth decade. We were able to test these hypotheses using multi-wave data from the Lothian Birth Cohort 1936 (LBC1936), a narrow-age cohort of over 1000 community-dwelling people, and longitudinal modelling of four waves of data that were collected from age 70 to 79 years. LBC1936 provides cognitive function data from age 11, as well as other control variables, such as education, which has been implicated as a mediator in the relationship between cognitive function in youth and cardiovascular risk,[22-24] and a variety of related behavioral and physical health variables.

Method

Participants

The Lothian Birth Cohort 1936 (LBC1936) is a community-dwelling sample of 1091 initially healthy individuals. All were born in 1936 and were at school in Scotland on 4 June 1947, when most took part in a group-administered intelligence test: the Moray House Test

For peer review only - http://bmjopen.bmj.com/site/about/guidelines.xhtml

(MHT) No. 12. They were followed up in four waves of one-to-one cognitive and health testing between 2004 and 2017, at mean ages 70 (*N* = 1091), 73 (*N* = 866), 76 (*N* = 697), and 79 (*N* = 550) years. Further details on the background, recruitment, attrition, and data collection procedures are available.[25 26] Participants provided written informed consent. Descriptive statistics for the individual participating in each wave of the study are presented in Table 1. Study completers only are described in supplementary table 1.

Cognitive functions

The Moray House Test No. 12 (MHT) is a broad cognitive ability test that includes word classification, proverbs, spatial items, and arithmetic. The test correlated about 0.8 at age 11 years with the Terman Merrill revision of the Stanford-Binet test, providing concurrent validity.[27]

In older age, cognitive function is known to show decline across multiple, but not all, subdomains.[28] We assessed fluid cognitive ability, processing speed, and memory, all of which decline on average with age. We also assessed crystallised ability, which remains relatively stable in later life.[29] There are strong correlations among the subdomains, and because of this, cognitive function can be modelled hierarchically, with a general cognitive function factor that captures overall ability. Beneath that general factor, specific subdomains capture variation beyond general cognitive function.[30] The relationships among cognitive tests and subdomains are described in the Statistical Analyses section below. Cognitive functions in waves 1–4 were assessed using 14 individually-administered cognitive tests at the same clinical research facility and using the same equipment and procedure for all four waves. The tests are fully described and referenced in an open-access protocol article.[26] The fluid subdomain consisted of matrix reasoning and block design from the Wechsler Adult Intelligence Scale (WAIS),[31] and spatial span forward and backward from the Wechsler Memory Scale (WMS.)[32] Processing speed was measured through symbol search and digit symbol substitution from the WAIS, plus four-choice reaction time[33] and inspection time.[34] Memory was assessed using verbal paired associates and logical memory from the WMS,[32]

For peer review only - http://bmjopen.bmj.com/site/about/guidelines.xhtml

and the letter–number sequencing and digit span backward subtests of the WAIS.[31] Crystallised ability was measured through the National Adult Reading Test,[35] Wechsler Test of Adult Reading,[36] and a phonemic verbal fluency test.[37]

Blood pressure

Blood pressure was measured six times at each wave, three times sitting and three times standing. Blood pressure measurements were divided into systolic pressure (maximum during one heartbeat) and diastolic pressure (minimum in between two heartbeats).

Adjustments for anti-hypertensive medications were made to both systolic and diastolic blood pressure. 15 points were added to systolic pressure and 10 points were added to diastolic pressure[38] if an individual was recorded as taking antihypertensive medication, such as atenolol, lisinopril, bisoprolol, etc. at that wave.

Covariates

Sex and education were both included as covariates in all models. Education was recorded as the total number of years spent in formal education. Age 11 cognitive function was also included in all models, as was the interaction between sex and age 11 function.

Several behavioural and diagnosed health variable were included. Smoking status was dummy coded with two variables, current smokers and ex-smokers, with non-smokers as the reference group. Participants were genotyped for the presence of an *APOE* ε4 allele. Cardiovascular disease and stroke history were both recorded for each wave; each was coded as a binary variable, with a 1 indicating that the individual had a history of the disease. Testing dates varied slightly for every individual at every wave, so the exact age of each participant at testing was also recorded.

For peer review only - http://bmjopen.bmj.com/site/about/guidelines.xhtml

Statistical analyses

All four LBC1936 Waves were analysed in latent growth curve models (LGCMs), a structural equation modelling technique that allows the user simultaneously to define and analyse multiple latent and measured variables.[39] For each variable modelled longitudinally, LGCMs model level (i.e. an intercept) and slope (i.e. a trajectory of change) parameters. We modelled blood pressure variables using a hierarchical ‘curve of factors’ model (supplementary figure 1). At the bottom tier were individual systolic and diastolic readings (only sitting readings were used for LGCMs), grouped by wave. For each grouping, both a systolic and diastolic latent variable was specified, creating four latent variables each for systolic and diastolic pressure, one each per wave. From these latent systolic and diastolic variables, latent variables modelling growth were specified, in much the same way as with cognitive function. To model overall blood pressure level, each of the eight lower-order latent variables contributed its unit loaded value (i.e. x1). For blood pressure, the best fit models were those that included both linear and quadratic slope latent variables. To model linear slope of blood pressure, linearly increasing loadings were given as the average age at a given wave progressed (x0, x2.96, x6.72, x9.79). To model quadratic slope, quadratically increasing loadings were assigned (x0, x8.76, x45.16, x95.84).

We modelled cognitive function using a slightly different hierarchical ‘factor of curves’ model, previously established with these data.[30] For each cognitive test we modelled a level (essentially the age 70 baseline) and a linear slope (the change between age 70 and age 79, taking all four measurement occasions into account) with the same loadings as with the age-respective blood pressure variables. For each cognitive domain (see Cognitive Function above) a latent level and linear slope variable was correspondingly composed of the individual tests’ latent level and latent slope variables. At the top of this cognitive hierarchy, latent levels and slopes for general cognitive function were formed from the level and slope variables of each cognitive domain. Similar models have been used and diagrams

For peer review only - http://bmjopen.bmj.com/site/about/guidelines.xhtml

presented elsewhere[30 40] and a structural diagram to illustrate these measured and latent variables and the associations among them is presented in supplementary figure 2.

Put simply, BP level is analogous to BP at the study's beginning (age 70 years), BP slope is analogous to the linear magnitude at which BP increases or decreases over the following decade, and BP quadratic (slope) is analogous to the curvature of the change in BP over the following decade. Cognitive function level is analogous to cognitive function at age 70, and is known to be highly correlated with cognitive function at age 11,[30] demonstrating some stability of individual differences in cognitive function across the life course. Slopes of cognitive function represent the rate at which cognitive functions change (mostly decrease, in fact) over subsequent waves, which is otherwise known as cognitive decline.

Covariates that applied to all waves of data (sex, age 11 cognitive function, their interaction, years of education, and smoking history) were associated with the intercept and slope parameters for latent levels and slopes, including those for the domains of cognitive function, but not onto individual cognitive tests' levels or slopes. *APOE* ε4 status only applied to cognitive function latent variables. Individual systolic and diastolic BP measurements were associated with characteristics of individual waves: age at testing, cardiovascular disease history, and stroke history.

Model parameters were estimated using full-information maximum likelihood, i.e. all data were used for all participants, even individuals who did not complete all waves. Standard errors were calculated using the robust Ruber-White method, and *P*-values were computed using the Yuan-Bentler scaled test statistic.[41] The false-discovery rate correction for multiple testing was applied to the variables in each model that were not control covariates, which included the associations between the latent variables of cognitive function and its subdomains and BP, as well as associations from sex, age 11 cognitive function, and their interaction. All analyses were conducted in the R programming language (version 3.3.2), using the 'lavaan' package for modelling.[42]

For peer review only - http://bmjopen.bmj.com/site/about/guidelines.xhtml
For peer review only

Patient and public involvement

LBC1936 participants were not involved in the development of any part of this study. The results will be disseminated to participants via a quarterly newsletter sent to LBC1936 participants.

Results

Modelling blood pressure

The best fit model of BP was a good fit for these data (χ^2 = 2052.76, df = 259, P < 0.001, CFI = 0.931, RMSEA = 0.080). The correlations between the level, linear slope, and quadratic slope latent BP variables were as follows: $r_{level,slope}$ = -0.466 (z = -10.216, P < 0.001), $r_{level,quad}$ = 0.323 (z = 5.525, P < 0.001), $r_{slope,quad}$ = -0.466 (z = -90.460, P < 0.001).

Blood pressure scores for each wave were derived from the latent variables and illustrated in Figure 1. In both hypertensive and normotensive individuals, blood pressure scores were similar at each age except for a notable but unsurprising difference in magnitude: individuals with hypertension had much higher blood pressure than those who did not have hypertension. Both groups show a quadratic effect, blood pressure rose between ages 70 and 76, and then declined between age 76 and 79.

Blood pressure and cognitive function

The best fit model of both blood pressure and cognitive function included latent variables for level and linear slope (χ^2 = 8911.01, df = 4023, P < 0.001, CFI = 0.928, RMSEA = 0.033). Models including a quadratic factor of cognitive function change either would not converge successfully or fit very poorly and could not be trusted to produce reliable estimates for cognitive function, and thus were not included in our models. Blood pressure was best modelled with a quadratic factor of change.

For peer review only

For peer review only - http://bmjopen.bmj.com/site/about/guidelines.xhtml

Overall, we found no associations between latent variables of cognitive function with those of blood pressure in our bivariate growth curve (Model 1A, Figure 2 & supplementary table 2). After correction for multiple comparisons, only three tested associations (out of 22) survived. First, a correlation between eighth decade cognitive function level and slope (r = 0.326, SE = 0.059, P < 0.001), which suggests that individuals with higher overall cognitive function have steeper cognitive decline over the eighth decade, likely because they have more functional ability to lose. Second, there was a regression path from age 11 cognitive function to eighth decade cognitive function level (β = 0.693, SE = 0.036, P < 0.001), which reproduces a well-known phenomenon: cognitive function shows substantial stability of individual differences across the life course,[43] and different measures taken at different times will still be strongly associated.

Third, a significant regression path was found from the interaction of age 11 cognitive function and sex to eighth decade blood pressure level (β = -0.136, SE = 0.050, P = 0.047). This interaction association indicates that women with higher cognitive function at age 11 have lower blood pressure in later life, and women with lower cognitive function have higher blood pressure. The opposite was true for men, higher cognitive function men had higher blood pressure and vice versa (Figure 3). No additional associations were found between any specific cognitive domains and blood pressure variables. All these tested associations are visualized in Figure 2.

Incorporating control variables into the LGCM (Model 1B, supplementary table 3) reduced the regression weight from the sex and age 11 cognitive function interaction to blood pressure level, so that it was no longer significant (β = -0.106, SE = 0.058, P = 0.215). The variable that most likely caused this change in the model was smoking behaviour; specifically, being a current smoker at age 70 had an association with eighth decade blood pressure (β = -0.104, SE = 0.040, post-hoc uncorrected P = 0.009).

For peer review only - http://bmjopen.bmj.com/site/about/guidelines.xhtml

Diagnosis and treatment effects

In an effort to understand why we found no associations between cognitive function and blood pressure in the eighth decade, we examined the role of medication in post-hoc analyses. Most individuals diagnosed with hypertension in the LBC1936 were prescribed medication for the condition. Following from Figure 1, though individuals with hypertension would have had higher blood pressure were they not being treated, they are being treated, and their blood pressure appears to generally decline over the eighth decade.

We fit linear mixed effect models to explore the associations of diagnosis and medication with blood pressure. Using latent variables for blood pressure, we generated blood pressure scores at each wave, but, by contrast with the previous analyses, the scores were based on the raw, medication-unadjusted systolic and diastolic blood pressure measurements. Using these scores, we first fit a mixed model predicting blood pressure score from a 2-way model that included the wave of the study, whether an individual had a hypertension diagnosis at that wave, and the interaction of the two (Model 2A, Table 2). We found a significant main effect of wave on blood pressure score (β = -0.048, SE = 0.014, P < 0.001) and interaction between wave and hypertension diagnosis (β = -0.111, SE = 0.028, P < 0.001), suggesting that individuals who are given a hypertension diagnosis have decreasing blood pressure over time.

In a second mixed effects model we replaced hypertension diagnosis by wave with a single variable indicating if a hypertension diagnosis was given at any wave, i.e. if a participant was diagnosed with hypertension during the eighth decade. This operationalization of diagnosis may capture more information than individual wave diagnoses; for example, an individual who first reports a hypertension diagnosis at wave 3 might already have hypertension at wave 2, and even if they do not have hypertension at wave 1, their blood pressure is likely to be elevated as they will be on their way to developing clinical hypertension. By coding all this information in a single variable we reduce the number of comparisons in the models as well as potentially improving our

For peer review only - http://bmjopen.bmj.com/site/about/guidelines.xhtml

operationalization. As a result (Model 2B, Table 2) we found similar associations as in model 2A, except that we also found a main effect of being diagnosed with hypertension at any wave, which suggests that individuals with a diagnosis generally had higher blood pressure as we would expect. However, the interaction suggests that over time, blood pressure decreased among individuals with hypertension. This suggests that diagnosed participants of this sample are taking their medication and this is having a significant, ameliorative effect on their raised blood pressure.

We fit two additional linear mixed effects models to examine associations between hypertension diagnosis and cognitive function. Because later life cognitive function is strongly associated with early life cognitive function, we included age 11 cognitive function as an independent variable in our regression models, and included all 2-way and 3-way interactions with wave and whether an individual was diagnosed with hypertension.

These models (Models 3A & 3B, Table 2) reproduced several established effects [30 43]. Age 11 cognitive function is associated with cognitive function scores in the eighth decade (β = 0.680, SE = 0.023, P < 0.001). Cognitive function declines across successive waves (β = -0.154, SE = 0.007, P < 0.001). Individuals with higher cognitive function early in life have more ability to lose, so declines are greater for these individuals across time (β = -0.039, SE = 0.014, P = 0.006).

In the second model of this set of models (Model 3B, Table 2), there was also a main effect of being diagnosed with hypertension at any wave and overall cognitive function level (β = -0.105, SE = 0.029, P < 0.001). Having a hypertension diagnosis was associated with lower cognitive function, which could not be fully explained by cognitive function scores from earlier in life. This suggests that hypertension may be associated with negative cognitive change between childhood and older age in this sample. Put another way, being diagnosed with hypertension did not have any protective associations with cognitive function across the eighth decade: it did not improve cognitive function or make up for the negative associations between blood pressure and cognitive decline. In previous models (Table 2) being

For peer review only - http://bmjopen.bmj.com/site/about/guidelines.xhtml

diagnosed with hypertension was associated with reduced blood pressure over time. Therefore, whereas hypertension diagnoses and anti-hypertensive medication appear to successfully treat high blood pressure in this cohort, they do not appear to impact associated cognitive deficiencies.

Discussion

Our results show that sex and cognitive function from early-life interact to predict blood pressure level during the eighth decade. Women with higher cognitive function are less likely than higher cognitively functioning men to have higher blood pressure, but the opposite is also true: lower cognitive function women are more likely to have higher blood pressure than similarly functioning men (Figure 3). This finding provides mixed support for our first hypothesis and the existing supporting literature,[18 21] as the effect was not robust to the inclusion of covariates. Some of this association can be explained by health behaviors and other health conditions, some of which stand out. Notably, whether one is a smoker or not appeared to explain some of the same variation as the sex by cognitive function interaction.

We did not find compelling evidence to support our second hypothesis. There were no associations between high blood pressure and cognitive function, either in level or trajectory of function, nor were any other health or sociodemographic variables associated with change in either blood pressure or cognitive function in the eighth decade. As a null result, this is a difficult finding to interpret. It may be that we did not have sufficient power to detect such associations in our sample, although nearly 40% of the sample had been diagnosed with hypertension by the initial wave at age 70. Another possibility is that cognitive function is most impacted by side effects of hypertension before the eighth decade: our findings tentatively support this, as individuals with a hypertension diagnosis tended to

For peer review only - http://bmjopen.bmj.com/site/about/guidelines.xhtml

have lower cognitive function, below what we would expect from baseline assessments at age 11.

Another possibility is that anti-hypertensive medications were effective at reducing hypertensive symptoms, which includes the connections between cognitive function and hypertension. We were able to demonstrate that anti-hypertensive medication was effective at lowering blood pressure, but we found no evidence that either medication or behavioral changes that might result from being diagnosed with hypertension have a positive association with subsequent cognitive function. In other words, treatment from antihypertensive medication in the eighth decade did not impact age or hypertension-related cognitive decline in this sample.

There is existing evidence for the importance of lifestyle factors in explaining the associations between cognitive function and physical health. In the National Longitudinal Study of Youth 1979, higher cognitive function women were less likely to be given a hypertension diagnosis,[21] an interaction that closely matches our initial finding in the LBC1936. However, this effect was explained by income differences. The Aberdeen Children of the 1950s cohort yielded results that were also similar to ours; specifically, associations between childhood cognitive function and both stroke and coronary artery events were present in both sexes, but stronger in women.[11] However, in their analyses, the sex by cognitive function interaction effects on stroke and CAD outcomes could be accounted for by education. In both of these cases, there were socioeconomic factors that seemed to drive the interaction between early life cognitive function and sex, whereas we found that behaviors, specifically smoking, seemed to explain some of the interaction. These samples vary in age and location, so chronological and geographic-cultural cohort differences might explain the discrepancies.[44 45]

The present study is limited by a non-trivial proportion of missing data, particularly from individuals who died over the course of the study, or were too frail or otherwise unwilling to continue participating. Moreover, our analytic sample was more affluent than the

For peer review only - http://bmjopen.bmj.com/site/about/guidelines.xhtml

average population, and may thus limit generalizability. The diagnoses and medications analyzed in the present study were self-reported and we were not able to cross-reference these reports with any physician records. Although we took steps to treat these variables conservatively in our analyses, self-reported diagnoses of hypertension tend to have lower validity than those drawn from medical records.[46]

In general, our results are consistent with previous work that has indicated that the effects of lower childhood cognitive function on hypertension are stronger and more consistent in women.[11 21] Despite there being known connections between high blood pressure and cognitive impairment, we did not find any evidence for this in the eighth decade. It appears that any cognitive lowering associated with hypertension may occur earlier in life. Future research ought to corroborate this and, in general, investigate the critical periods when hypertension might be detrimental to cognitive health.

For peer review only

For peer review only - http://bmjopen.bmj.com/site/about/guidelines.xhtml

Contributors: DMA discussed and planned the study and analyses, analysed the data, interpreted the data and drafted the initial manuscript. JMS discussed and planned the study and analyses, interpreted the data and contributed to the manuscript. IJD discussed and planned the study and analyses, interpreted the data and contributed to the manuscript.

Competing interests: The authors declare that they have no conflicts of interest.

Funding statement: The LBC1936 data were collected using a Research Into Ageing programme grant; this research continues as part of the Age UK-funded Disconnected Mind project. DMA, JMS, and IJD are members of The University of Edinburgh Centre for Cognitive Ageing and Cognitive Epidemiology, which is funded by the Biotechnology and Biological Sciences Research Council and Medical Research Council (MR/K026992/1). DMA is also funded by an MRC Mental Health Data Pathfinder award (MC_PC_17209).

Ethics approval: Ethics permissions were obtained from the Multicentre Research Ethics Committee for Scotland (wave 1, MREC/01/0/56), the Lothian Research Ethics Committee (wave 1, LREC/2003/2/29), and the Scotland a Research Ethics Committee (waves 2–4, 07/MRE00/58).

Data sharing statement: Lothian Birth Cohort 1936 data can be requested from the Lothian Birth Cohort 1936 research team, following completion of a data request application. More information can be found online (http://www.lothianbirthcohort.ed.ac.uk/content/collaboration).

Acknowledgements

The LBC1936 study is funded by Age UK (Disconnected Mind project). We thank the Scottish Council for Research in Education for allowing access to the SMS1947. We thank the LBC1936 Study participants and research team members.

For peer review only

References

1. Staessen JA, Gasowski J, Wang JG, et al. Risks of untreated and treated isolated systolic hypertension in the elderly: meta-analysis of outcome trials. *The Lancet* 2000;355(9207):865-72.
2. Beckett NS, Peters R, Fletcher AE, et al. Treatment of hypertension in patients 80 years of age or older. *New Engl J Med* 2008;358(18):1887-98.
3. McInnes GT. Hypertension and coronary artery disease: cause and effect. *Journal of Hypertension* 1995;13:S49-S56.
4. Barnes DE, Yaffe K. The projected effect of risk factor reduction on Alzheimer's disease prevalence. *The Lancet Neurology* 2011;10(9):819-28.
5. Salthouse T. Consequences of age-related cognitive declines. *Annual review of psychology* 2012;63:201-26.
6. Iadecola C, Yaffe K, Biller J, et al. Impact of hypertension on cognitive function: a scientific statement from the American Heart Association. *Hypertension* 2016;68(6):e67-e94.
7. Matthews F, Stephan B, Robinson L, et al. A two decade dementia incidence comparison from the Cognitive Function and Ageing Studies I and II. *Nature communications* 2016;7
8. Hughes TM, Sink KM. Hypertension and its role in cognitive function: current evidence and challenges for the future. *American journal of hypertension* 2015;29(2):149-57.
9. Birns J, Kalra L. Cognitive function and hypertension. *Journal of human hypertension* 2009;23(2):86.
10. Wraw C, Deary IJ, Gale CR, et al. Intelligence in youth and health at age 50. *Intelligence* 2015;53:23-32.
11. Lawlor DA, Batty GD, Clark H, et al. Association of childhood intelligence with risk of coronary heart disease and stroke: findings from the Aberdeen Children of the 1950s cohort study. *Eur J Epidemiol* 2008;23(10):695-706.
12. Calvin CM, Batty GD, Deary IJ. Cognitive epidemiology. *The Wiley-Blackwell Handbook of Individual Differences* 2011:427-60.
13. Batty GD, Deary IJ, Gottfredson LS. Premorbid (early life) IQ and later mortality risk: Systematic review. *Ann Epidemiol* 2007;17:278-88.
14. Deary IJ. Cognitive epidemiology: Its rise, its current issues, and its challenges. *Personality and individual differences* 2010;49(4):337-43.
15. Deary IJ. Looking for 'system integrity'in cognitive epidemiology. *Gerontology* 2012;58(6):545-53.
16. Lubinski D. Cognitive epidemiology: With emphasis on untangling cognitive ability and socioeconomic status. *Intelligence* 2009;37(6):625-33.
17. Jousilahti P, Vartiainen E, Tuomilehto J, et al. Sex, age, cardiovascular risk factors, and coronary heart disease. *Circulation* 1999;99(9):1165-72.
18. Sandberg K, Ji H. Sex differences in primary hypertension. *Biology of sex differences* 2012;3(1):7.
19. Bots SH, Peters SA, Woodward M. Sex differences in coronary heart disease and stroke mortality: a global assessment of the effect of ageing between 1980 and 2010. *BMJ global health* 2017;2(2):e000298.
20. Courtenay WH. Constructions of masculinity and their influence on men's well-being: a theory of gender and health. *Social science & medicine* 2000;50(10):1385-401.
21. Altschul DM, Wraw C, Der G, et al. Hypertension development by midlife and the roles of pre-morbid cognitive function, sex, and their interaction. *Hypertension* 2019
22. Hagger-Johnson G, Mõttus R, Craig LC, et al. Pathways from childhood intelligence and socioeconomic status to late-life cardiovascular disease risk. *Health Psychol* 2012;31(4):403.
23. Leng B, Jin Y, Li G, et al. Socioeconomic status and hypertension: a meta-analysis. *Journal of hypertension* 2015;33(2):221-29.

For peer review only - http://bmjopen.bmj.com/site/about/guidelines.xhtml

24. Backholer K, Peters SAE, Bots SH, et al. Sex differences in the relationship between socioeconomic status and cardiovascular disease: a systematic review and meta-analysis. *J Epidemiol Community Health* 2017;71(6):550-57. doi: 10.1136/jech-2016-207890
25. Taylor AM, Pattie A, Deary IJ. Cohort Profile Update: The Lothian Birth Cohorts of 1921 and 1936. *Int J Epidemiol* 2018;47(4):1042-42r. doi: 10.1093/ije/dyy022
26. Deary IJ, Gow AJ, Taylor MD, et al. The Lothian Birth Cohort 1936: a study to examine influences on cognitive ageing from age 11 to age 70 and beyond. *BMC Geriatr* 2007;7(1):28.
27. Deary IJ, Whalley LJ, Starr JM. A lifetime of intelligence: Follow-up studies of the Scottish mental surveys of 1932 and 1947: American Psychological Association 2009.
28. Verhaeghen P, Salthouse TA. Meta-analyses of age–cognition relations in adulthood: Estimates of linear and nonlinear age effects and structural models. *Psychological bulletin* 1997;122(3):231.
29. Horn JL, Cattell RB. Age differences in fluid and crystallized intelligence. *Acta psychologica* 1967;26:107-29.
30. Ritchie SJ, Tucker-Drob EM, Cox SR, et al. Predictors of ageing-related decline across multiple cognitive functions. *Intelligence* 2016;59:115-26.
31. Wechsler D. Adult intelligence scale. *New York* 1997;21
32. Wechsler D. Wechsler memory scale. 1945
33. Deary IJ, Der G, Ford G. Reaction times and intelligence differences: A population-based cohort study. *Intelligence* 2001;29:389–99.
34. Deary IJ, Johnson W, Starr JM. Are processing speed tasks biomarkers of cognitive aging? *Psychology and aging* 2010;25(1):219.
35. Nelson HE, Willison J. National adult reading test (NART): Nfer-Nelson Windsor 1991.
36. Wechsler D. Wechsler Test of Adult Reading: WTAR: Psychological Corporation 2001.
37. Lezak MD. Neuropsychological assessment: Oxford University Press, USA 2004.
38. Tobin MD, Sheehan NA, Scurrah KJ, et al. Adjusting for treatment effects in studies of quantitative traits: antihypertensive therapy and systolic blood pressure. *Stat Med* 2005;24(19):2911-35. doi: 10.1002/sim.2165
39. Preacher KJ. Latent growth curve modeling: Sage 2008.
40. Altschul DM, Starr JM, Deary IJ. Cognitive function in early and later life is associated with blood glucose in older individuals: analysis of the Lothian Birth Cohort of 1936. *Diabetologia* 2018;61(9):1946-55.
41. Yuan K-H, Bentler PM. Mean and covariance structure analysis: Theoretical and practical improvements. *Journal of the American statistical association* 1997;92(438):767-74.
42. Rosseel Y. lavaan: an R package for structural equation modeling and more Version 0.4-9 (BETA): Ghent University, 2011.
43. Deary IJ, Pattie A, Starr JM. The stability of intelligence from age 11 to age 90 years: the Lothian birth cohort of 1921. *Psychol Sci* 2013;24(12):2361-68.
44. Tucker-Drob EM, Bates TC. Large cross-national differences in gene× socioeconomic status interaction on intelligence. *Psychol Sci* 2016;27(2):138-49.
45. Allender S, Scarborough P, O'Flaherty M, et al. Patterns of coronary heart disease mortality over the 20 th century in England and Wales: Possible plateaus in the rate of decline. *BMC Public Health* 2008;8(1):148.
46. Gonçalves VS, Andrade KR, Carvalho K, et al. Accuracy of self-reported hypertension: a systematic review and meta-analysis. *Journal of hypertension* 2018;36(5):970-78.

For peer review only

For peer review only - http://bmjopen.bmj.com/site/about/guidelines.xhtml

Table 1. Descriptive statistics for cognitive, demographic, and clinical variables.

	Wave 1	Wave 2	Wave 3	Wave 4
Total	1091	866	697	550
Female	543 (49.8%)	418 (48.3%)	337 (48.4%)	275 (50.0%)
Ever had high BP	433 (39.7%)	425 (49.1%)	378 (54.2%)	317 (57.6%)
Ever had CVD	268 (24.6%)	250 (28.9%)	236 (33.95)	204 (37.1%)
Ever had stroke	54 (4.95%)	55 (6.35%)	73 (10.5%)	69 (12.5%)
Current smoker	125 (11.5%)	73 (8.43%)	44 (6.31%)	12 (3.81%)
Ex-smoker	465 (42.6%)	378 (43.6%)	293 (42.0%)	232 (42.2%)
Average sitting systolic BP	149.6 (19.04)	148.7 (18.83)	148.1 (19.31)	145.1 (18.8)
Average sitting diastolic BP	81.3 (10.14)	78.0 (9.81)	78.9 (10.3)	76.8 (10.1)
Years of education	10.7 (1.13)	10.8 (1.14)	10.8 (1.14)	10.9 (1.18)
Age 11 IQ	100.0 (14.99)	100.7 (15.3)	101.5 (15.3)	101.9 (15.3)
Matrix Reasoning*	13.5 (5.13)	13.2 (4.96)	13.0 (4.91)	12.9 (5.03)
Block Design*	33.8 (10.3)	33.6 (10.1)	32.2 (9.95)	31.2 (9.63)
Spatial Span*	7.04 (1.74)	7.06 (1.61)	7.05 (1.59)	6.74 (1.60)
NART†	34.5 (8.15)	34.4 (8.18)	35.0 (8.03)	35.6 (8.19)
WTAR†	41.0 (7.17)	41.0 (6.97)	41.1 (7.02)	41.6 (7.03)
Verbal Fluency†	42.4 (12.5)	43.2 (12.9)	42.9 (12.8)	43.6 (13.3)
Logical Memory‡	71.5 (18.0)	74.3 (17.9)	74.6 (19.2)	72.7 (20.4)
VPA‡	26.4 (9.13)	27.2 (9.46)	26.4 (9.56)	27.1 (9.55)
Digit Span‡	7.7 (2.26)	7.81 (2.29)	7.77 (2.37)	7.56 (2.18)
LNS‡	10.9 (3.16)	10.9 (3.08)	10.5 (2.99)	10.1 (2.89)
Symbol Search§	24.7 (6.39)	24.6 (6.18)	24.6 (6.46)	22.7 (6.63)
Digit-Symbol Coding§	56.6 (12.9)	56.4 (12.3)	53.8 (12.9)	51.2 (13.0)
Inspection Time§	112 (11.0)	111 (11.8)	110 (12.5)	107 (13.6)
Reaction Time§	0.64 (0.09)	0.65 (0.09)	0.68 (0.10)	0.71 (0.11)
MMSE	28.8 (1.43)	28.8 (1.42)	28.6 (1.70)	28.48 (2.16)

CVD, cardiovascular disease; BP, blood pressure; NART, National Adult Reading Test; WTAR, Wechsler Test of Adult Reading; VPA, Visual Paired Associates; LNS, Letter-Number Sequencing; MMSE, Mini-Mental State Examination; T2D, individuals with type 2 diabetes; T2D was defined as self-reported physician diagnosis of diabetes. * part of the visuospatial ability domain, † part of the crystallized ability domain, ‡ part of the memory domain, § part of the processing speed domain.

For peer review only - http://bmjopen.bmj.com/site/about/guidelines.xhtml

Table 2. Linear mixed effects regression models of blood pressure and cognitive function scores predicted by antihypertensive treatment over time.

Variable	Std Estimate	SE	Wald χ2	*P*
Predicting blood pressure score - Model 2A				
Wave	-0.048	0.014	11.854	< 0.001
Did the individual have HT at this wave?	-0.031	0.023	0.339	0.560
Wave x having HT	-0.111	0.028	15.370	< 0.001
Predicting blood pressure score - Model 2B				
Wave	-0.086	0.015	20.929	< 0.001
Did the individual have HT at any wave?	0.170	0.031	35.162	< 0.001
Wave x having HT	-0.208	0.032	43.536	< 0.001
Predicting cognitive function score - Model 3A				
Wave	-0.154	0.007	490.085	< 0.001
Did the individual have HT at **this** wave?	-0.008	0.013	0.552	0.458
Age 11 cognitive function	0.680	0.023	945.862	< 0.001
Wave x having HT	0.006	0.015	0.148	0.701
Wave x age 11 cognitive function	-0.039	0.014	7.512	0.006
Having HT x age 11 cognitive function	-0.037	0.028	1.751	0.186
Wave x having HT x age 11 function	-0.003	0.029	0.009	0.923
Predicting cognitive function score - Model 3B				
Wave	-0.157	0.007	517.451	< 0.001
Did the individual have HT at **any** wave?	-0.105	0.029	13.230	< 0.001
Age 11 cognitive function	0.678	0.025	754.084	< 0.001
Wave x having HT	-0.007	0.015	0.284	0.594
Wave x age 11 cognitive function	-0.041	0.015	7.747	0.005
Having HT x age 11 cognitive function	0.020	0.054	0.165	0.685
Wave x having HT x age 11 function	-0.014	0.029	0.216	0.642

For peer review only

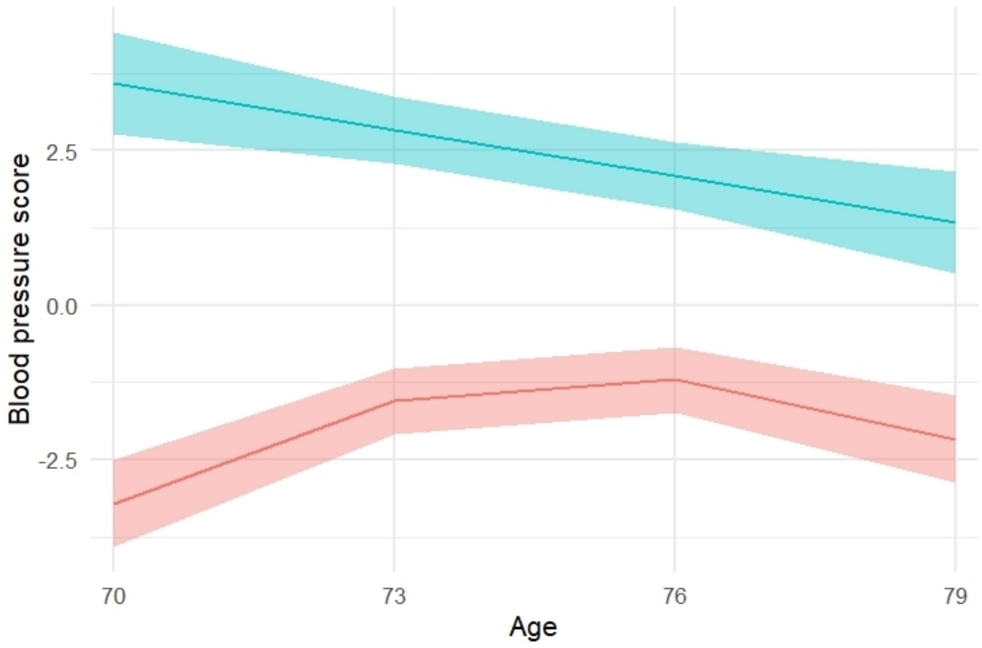

Figure 1. Blood pressure trajectories in the eighth decade for individuals with and without hypertension diagnoses. Only study completers, those participants who were present at every wave of the study, were included in this plot. All study participants were used in the statistical analyses. Blood pressure score was derived from the latent variable that represent blood pressure in individual waves. Blood pressure score is a standardized, unitless measure of overall blood pressure magnitude; A score of 0 indicates the individual is in line with average blood pressure, and 1 would indicate that an individual was 1SD higher blood pressure at that time. The upper green line illustrates individuals diagnosed with hypertension at any wave (n = 337) and the lower red line illustrates normotensive individuals (n = 204). The shaded areas represent 95% confidence regions.

63x41mm (300 x 300 DPI)

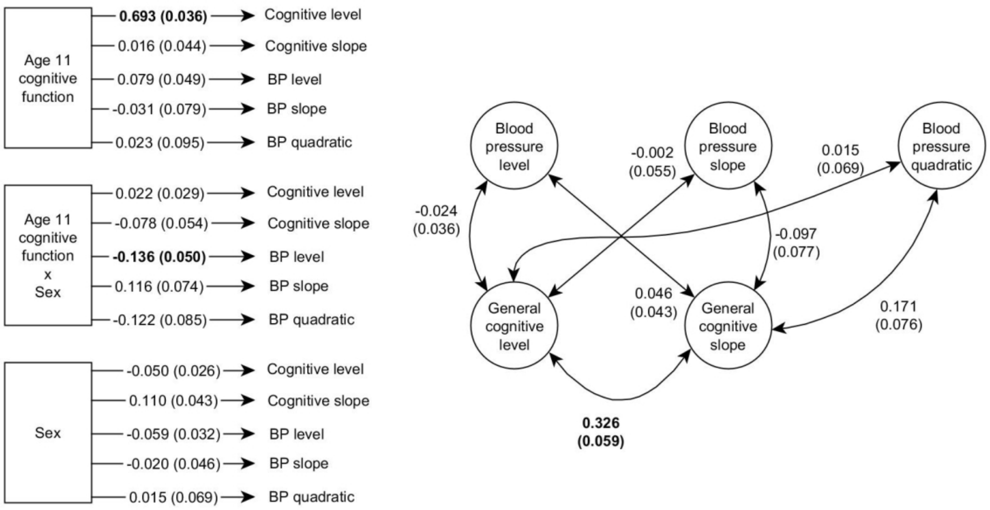

Figure 2. Path diagram displaying the statistical tests in a latent growth curve model of cognitive function and blood pressure. Circles represent latent variables and squares represent measured variables. Double arrowed lines represent correlations. Single arrowed lines (on the left side) represent regressions. Coefficients and SEs are the result of model 1A; numbers printed in bold indicate coefficients that were statistically significant after correction for multiple comparisons.

136x73mm (300 x 300 DPI)

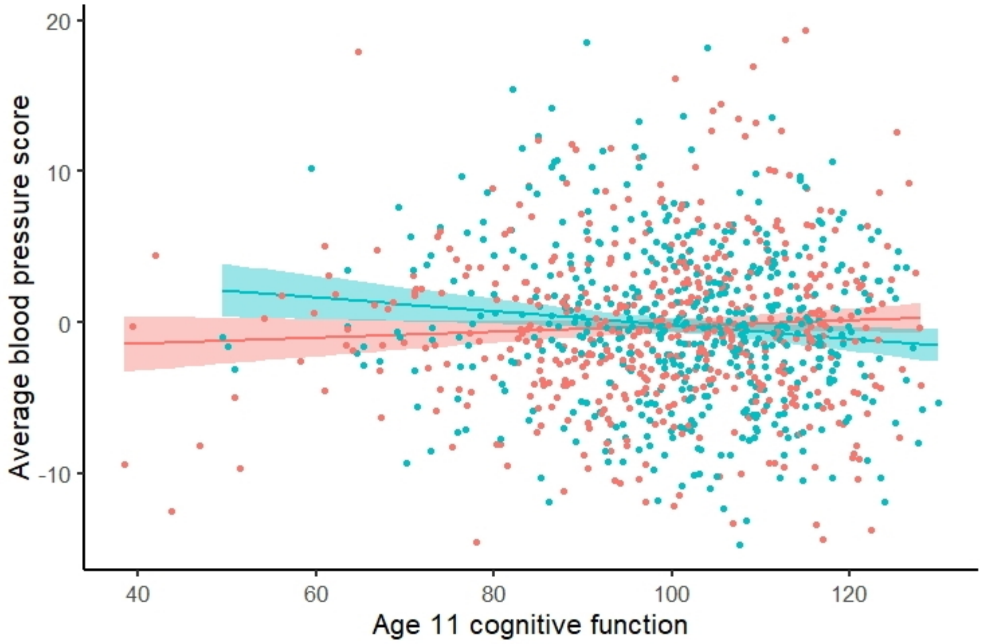

Figure 3. Blood pressure in older age and its relationship with cognitive function at age 11 years, sex, and their interaction. Blood pressure score was derived from the latent variable that represent blood pressure in individual waves. The green dots and line represent women, and the red dots and line represent men. The shaded areas represent 95% confidence regions.

63x41mm (300 x 300 DPI)

Blood pressure and cognitive function across the eighth decade: a prospective study of the Lothian Birth Cohort of 1936

Supplementary Materials

Supplementary Figures 1, 2

Supplementary Tables 1 – 3

For peer review only

Supplementary Figure 1. Simplified path diagram of the hierarchical curve of factors latent growth model of blood pressure change in the LBC1936. Circles represent latent variables and squares represent measured variables. Growth curves, including latent level, linear slope, and quadrative slope factors, were estimated from both systolic and diastolic reading. Basis coefficients (loadings on the individual test slopes) were fixed at 0, 2.96, 6.72, and 9.79 for the linear slops and 0, 8.76, 45.16, and 95.84 for the quadratic slopes to precisely reflect the amount of time passing between assessments. Dashed lines indicate the relationships between covariates which differed between waves and their effects on the individual measurements at different waves. To preserve interpretability, not all variables and paths are shown. Solid, double arrowed lines indicate correlations between latent variables. Dashed lines ending in diamonds represent systolic and diastolic measurements, modelled the same way as at age 70.

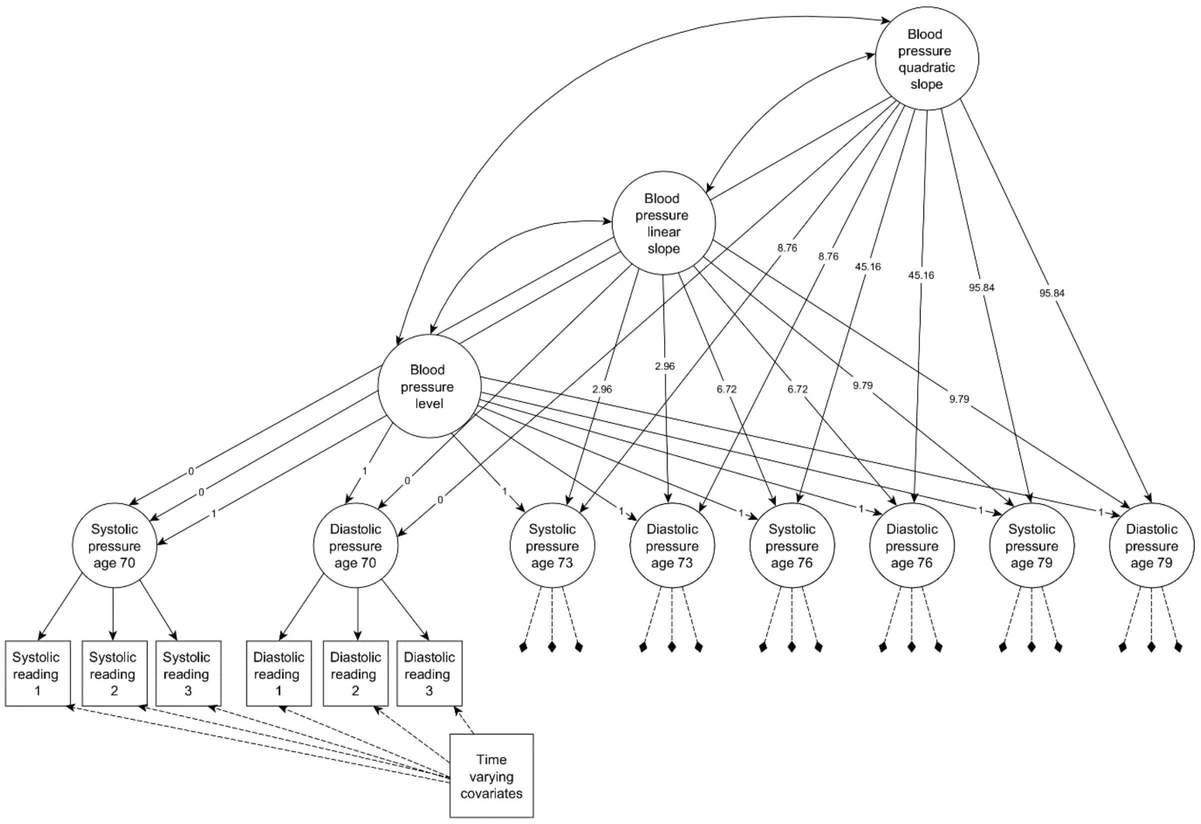

Supplementary Figure 2. Simplified path diagram of the bivariate growth curves of blood pressure and cognitive function, as well as the underlying structure of cognitive function. Circles represent latent variables and squares represent measured variables. Latent variables for blood pressure were modelled as shown in Figure 1. Growth curves, including a latent level and linear slope factor, were estimated for each cognitive test, and the intercepts and slopes were analyzed in a hierarchical model which contained domain and overall factors of both level and slope. Basis coefficients (loadings on the individual test slopes) were fixed at 0, 2.96, 6.72, and 9.79 to precisely represent the amount of time passing between assessments. Dashed lines indicate the relationships between covariates which were fixed across waves and the overall and domain latent variables. To preserve interpretability, not all covariate relationships are shown. Solid, double arrowed lines indicate correlations between latent variables. Although only two domains and four tests are shown here, the full model used all fourteen tests and four domains, as described in the Methods.

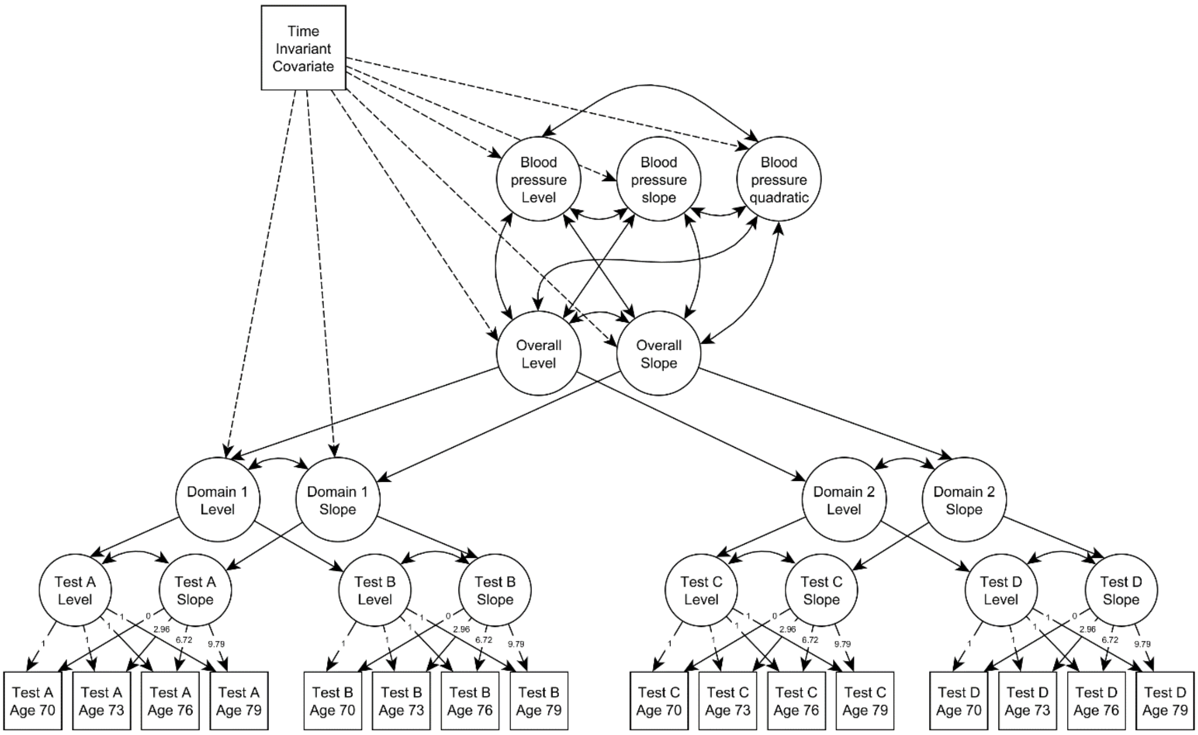

Supplementary Table 1. Descriptive statistics for cognitive, demographic, and clinical variables from study completers only (n = 539).

	Wave 1	Wave 2	Wave 3	Wave 4
Female	267 (49.5%)	-	-	-
Ever had high BP	193 (35.8%)	256 (47.5%)	286 (53.1%)	314 (58.3%)
Ever had CVD	123 (22.8%)	151 (28.0%)	185 (34.3%)	198 (36.7%)
Ever had stroke	18 (3.33%)	31 (5.75%)	55 (10.2%)	69 (12.5%)
Current smoker	24 (4.54%)	22 (4.08%)	19 (3.52%)	21 (3.90%)
Ex-smoker	226 (41.9%)	228 (42.3%)	222 (41.2%)	229 (42.4%)
Average sitting systolic BP	147.7 (17.63)	147.7 (18.18)	147.7 (18.75)	145.1 (18.7)
Average sitting diastolic BP	81.0 (9.53)	77.9 (9.53)	79.0 (10.3)	76.8 (10.1)
Years of education	10.9 (1.17)	-	-	-
Age 11 IQ	102.2 (15.15)	-	-	-
Matrix Reasoning*	14.7 (5.02)	13.2 (4.89)	13.3 (4.91)	13.0 (5.02)
Block Design*	35.7 (10.1)	34.7 (10.1)	32.7 (9.79)	31.3 (9.66)
Spatial Span*	7.27 (1.71)	7.15 (1.58)	7.15 (1.58)	6.75 (1.60)
NART†	35.6 (7.97)	35.2 (7.99)	35.6 (7.95)	35.7 (8.10)
WTAR†	41.9 (6.95)	41.8 (6.61)	41.6 (6.93)	41.7 (6.97)
Verbal Fluency†	43.6 (12.8)	44.4 (13.0)	43.7 (12.7)	43.7 (13.3)
Logical Memory‡	74.4 (16.9)	76.3 (16.9)	75.8 (18.4)	73.0 (20.3)
VPA‡	27.9 (8.53)	28.4 (9.04)	27.3 (9.25)	27.3 (9.44)
Digit Span‡	8.03 (2.37)	8.01 (2.32)	7.91 (2.41)	7.58 (2.19)
LNS‡	11.4 (3.11)	11.4 (3.08)	10.7 (2.98)	10.1 (2.87)
Symbol Search§	25.6 (6.58)	25.5 (5.92)	25.3 (6.26)	22.8 (6.64)
Digit-Symbol Coding§	59.0 (12.4)	58.4 (12.0)	55.4 (12.3)	51.4 (13.0)
Inspection Time§	113 (10.5)	112 (11.7)	111 (11.8)	107 (13.6)
Reaction Time§	0.63 (0.08)	0.64 (0.08)	0.67 (0.09)	0.70 (0.11)
MMSE	28.9 (1.28)	28.9 (1.21)	28.8 (1.53)	28.5 (2.16)

CVD, cardiovascular disease; BP, blood pressure; NART, National Adult Reading Test; WTAR, Wechsler Test of Adult Reading; VPA, Visual Paired Associates; LNS, Letter-Number Sequencing; MMSE, Mini-Mental State Examination; T2D, individuals with type 2 diabetes; T2D was defined as self-reported physician diagnosis of diabetes. * part of the visuospatial ability domain, † part of the crystallized ability domain, ‡ part of the memory domain, § part of the processing speed domain.

For peer review only

Supplementary Table 2. Bivariate associations of the latent growth curve model (1A) of blood pressure and cognitive function.

LHS	Path	RHS	Std. Estimate	S.E.
Blood pressure level	~~	Blood pressure quadratic	0.293	0.065
Blood pressure slope	~~	Blood pressure quadratic	-0.926	0.017
Blood pressure level	~~	Blood pressure slope	-0.424	0.052
Cognitive function level	~~	Cognitive function slope	0.326	0.059
Fluid function level	~~	Fluid function slope	-0.570	1.150
Processing speed level	~~	Processing speed slope	-0.255	0.123
Memory ability level	~~	Memory ability slope	-0.394	0.108
Crystiallized ability level	~~	Crystiallized ability slope	-0.277	0.377
Blood pressure level	~~	Cognitive function level	-0.024	0.036
Blood pressure slope	~~	Cognitive function slope	-0.097	0.077
Blood pressure quadratic	~~	Cognitive function slope	0.171	0.076
Blood pressure level	~~	Cognitive function slope	0.046	0.043
Blood pressure slope	~~	Cognitive function level	-0.003	0.055
Blood pressure quadratic	~~	Cognitive function level	0.015	0.069
Cognitive function slope	~	Sex	0.110	0.043
Cognitive function slope	~	Age 11 cognitive function	0.016	0.044
Cognitive function slope	~	Education	-0.069	0.049
Cognitive function slope	~	Sex x age 11 CF	-0.078	0.054
Blood pressure quadratic	~	Sex	0.102	0.054
Blood pressure quadratic	~	Age 11 cognitive function	0.023	0.095
Blood pressure quadratic	~	Education	0.060	0.060
Blood pressure quadratic	~	Sex x age 11 CF	-0.122	0.085
Blood pressure slope	~	Sex	-0.020	0.048
Blood pressure slope	~	Age 11 cognitive function	-0.031	0.079
Blood pressure slope	~	Education	-0.058	0.059
Blood pressure slope	~	Sex x age 11 CF	0.116	0.074

For peer review only

Blood pressure level	~	Sex	-0.059	0.032
Blood pressure level	~	Age 11 cognitive function	0.079	0.049
Blood pressure level	~	Education	-0.024	0.038
Blood pressure level	~	Sex x age 11 CF	-0.136	0.050
Cognitive function level	~	Sex x age 11 CF	0.022	0.029
Cognitive function level	~	Sex	-0.050	0.026
Cognitive function level	~	Age 11 cognitive function	0.694	0.037
Cognitive function level	~	Education	0.222	0.019
Systolic pressure measurement 1, wave 1	~	Age at wave 1	-0.010	0.025
Systolic pressure measurement 2, wave 1	~	Age at wave 1	-0.040	0.024
Systolic pressure measurement 3, wave 1	~	Age at wave 1	-0.057	0.028
Diastolic pressure measurement 1, wave 1	~	Age at wave 1	-0.074	0.025
Diastolic pressure measurement 2, wave 1	~	Age at wave 1	-0.061	0.027
Diastolic pressure measurement 3, wave 1	~	Age at wave 1	-0.089	0.028
Systolic pressure measurement 1, wave 2	~	Age at wave 2	-0.029	0.030
Systolic pressure measurement 2, wave 2	~	Age at wave 2	0.001	0.029
Systolic pressure measurement 3, wave 2	~	Age at wave 2	0.010	0.033
Diastolic pressure measurement 1, wave 2	~	Age at wave 2	0.019	0.039
Diastolic pressure measurement 2, wave 2	~	Age at wave 2	0.020	0.035
Diastolic pressure measurement 3, wave 2	~	Age at wave 2	0.014	0.033
Systolic pressure measurement 1, wave 3	~	Age at wave 3	-0.069	0.036
Systolic pressure measurement 2, wave 3	~	Age at wave 3	-0.092	0.032
Systolic pressure measurement 3, wave 3	~	Age at wave 3	-0.081	0.034
Diastolic pressure measurement 1, wave 3	~	Age at wave 3	0.026	0.038
Diastolic pressure measurement 2, wave 3	~	Age at wave 3	0.028	0.036
Diastolic pressure measurement 3, wave 3	~	Age at wave 3	0.014	0.039
Systolic pressure measurement 1, wave 4	~	Age at wave 4	-0.043	0.032
Systolic pressure measurement 2, wave 4	~	Age at wave 4	-0.077	0.027
Systolic pressure measurement 3, wave 4	~	Age at wave 4	-0.051	0.028
Diastolic pressure measurement 1, wave 4	~	Age at wave 4	0.049	0.042
Diastolic pressure measurement 2, wave 4	~	Age at wave 4	0.039	0.030

For peer review only

Diastolic pressure measurement 3, wave 4	~	Age at wave 4	0.017	0.026
Systolic pressure measurement 1, wave 1	~~	Systolic pressure measurement 1, wave 1	0.131	0.010
Systolic pressure measurement 2, wave 1	~~	Systolic pressure measurement 2, wave 1	0.074	0.008
Systolic pressure measurement 3, wave 1	~~	Systolic pressure measurement 3, wave 1	0.097	0.008
Diastolic pressure measurement 1, wave 1	~~	Diastolic pressure measurement 1, wave 1	0.141	0.014
Diastolic pressure measurement 2, wave 1	~~	Diastolic pressure measurement 2, wave 1	0.093	0.014
Diastolic pressure measurement 3, wave 1	~~	Diastolic pressure measurement 3, wave 1	0.150	0.015
Systolic pressure measurement 1, wave 2	~~	Systolic pressure measurement 1, wave 2	0.247	0.018
Systolic pressure measurement 2, wave 2	~~	Systolic pressure measurement 2, wave 2	0.039	0.008
Systolic pressure measurement 3, wave 2	~~	Systolic pressure measurement 3, wave 2	0.069	0.007
Diastolic pressure measurement 1, wave 2	~~	Diastolic pressure measurement 1, wave 2	0.113	0.013
Diastolic pressure measurement 2, wave 2	~~	Diastolic pressure measurement 2, wave 2	0.082	0.011
Diastolic pressure measurement 3, wave 2	~~	Diastolic pressure measurement 3, wave 2	0.094	0.016
Systolic pressure measurement 1, wave 3	~~	Systolic pressure measurement 1, wave 3	0.155	0.015
Systolic pressure measurement 2, wave 3	~~	Systolic pressure measurement 2, wave 3	0.068	0.013
Systolic pressure measurement 3, wave 3	~~	Systolic pressure measurement 3, wave 3	0.085	0.013
Diastolic pressure measurement 1, wave 3	~~	Diastolic pressure measurement 1, wave 3	0.144	0.021
Diastolic pressure measurement 2, wave 3	~~	Diastolic pressure measurement 2, wave 3	0.137	0.029
Diastolic pressure measurement 3, wave 3	~~	Diastolic pressure measurement 3, wave 3	0.168	0.016
Systolic pressure measurement 1, wave 4	~~	Systolic pressure measurement 1, wave 4	0.113	0.014
Systolic pressure measurement 2, wave 4	~~	Systolic pressure measurement 2, wave 4	0.045	0.009
Systolic pressure measurement 3, wave 4	~~	Systolic pressure measurement 3, wave 4	0.093	0.012
Diastolic pressure measurement 1, wave 4	~~	Diastolic pressure measurement 1, wave 4	0.140	0.025
Diastolic pressure measurement 2, wave 4	~~	Diastolic pressure measurement 2, wave 4	0.093	0.015
Diastolic pressure measurement 3, wave 4	~~	Diastolic pressure measurement 3, wave 4	0.102	0.011
Blood pressure level	~~	Blood pressure level	0.985	0.008
Blood pressure slope	~~	Blood pressure slope	0.989	0.013
Blood pressure quadratic	~~	Blood pressure quadratic	0.979	0.019
Fluid function level	~~	Fluid function level	0.389	0.045
Processing speed level	~~	Processing speed level	0.449	0.044
Memory ability level	~~	Memory ability level	0.258	0.034

For peer review only

Crystiallized ability level	~~	Crystiallized ability level	0.343	0.023
Cognitive function level	~~	Cognitive function level	0.314	0.028
Fluid function slope	~~	Fluid function slope	0.053	0.241
Processing speed slope	~~	Processing speed slope	0.177	0.089
Memory ability slope	~~	Memory ability slope	0.369	0.065
Crystiallized ability slope	~~	Crystiallized ability slope	0.311	0.655
Cognitive function slope	~~	Cognitive function slope	0.977	0.012
Sex	~~	Sex	1.000	0.000
Sex	~~	Age 11 cognitive function	0.065	0.000
Sex	~~	Education	-0.008	0.000
Sex	~~	Sex x age 11 CF	0.051	0.000
Sex	~~	Age at wave 1	0.004	0.000
Sex	~~	Age at wave 2	0.022	0.000
Sex	~~	Age at wave 3	-0.009	0.000
Sex	~~	Age at wave 4	0.001	0.000
Age 11 cognitive function	~~	Age 11 cognitive function	1.000	0.000
Age 11 cognitive function	~~	Education	0.432	0.000
Age 11 cognitive function	~~	Sex x age 11 CF	0.660	0.000
Age 11 cognitive function	~~	Age at wave 1	-0.097	0.000
Age 11 cognitive function	~~	Age at wave 2	-0.084	0.000
Age 11 cognitive function	~~	Age at wave 3	-0.103	0.000
Age 11 cognitive function	~~	Age at wave 4	-0.095	0.000
Education	~~	Education	1.000	0.000
Education	~~	Sex x age 11 CF	0.296	0.000
Education	~~	Age at wave 1	-0.075	0.000
Education	~~	Age at wave 2	-0.047	0.000
Education	~~	Age at wave 3	-0.085	0.000
Education	~~	Age at wave 4	-0.066	0.000
Sex x age 11 CF	~~	Sex x age 11 CF	1.000	0.000
Sex x age 11 CF	~~	Age at wave 1	-0.073	0.000
Sex x age 11 CF	~~	Age at wave 2	-0.065	0.000

For peer review only

Sex x age 11 CF	~~	Age at wave 3	-0.098	0.000
Sex x age 11 CF	~~	Age at wave 4	-0.086	0.000
Age at wave 1	~~	Age at wave 1	1.000	0.000
Age at wave 1	~~	Age at wave 2	0.945	0.000
Age at wave 1	~~	Age at wave 3	0.936	0.000
Age at wave 1	~~	Age at wave 4	0.893	0.000
Age at wave 2	~~	Age at wave 2	1.000	0.000
Age at wave 2	~~	Age at wave 3	0.922	0.000
Age at wave 2	~~	Age at wave 4	0.880	0.000
Age at wave 3	~~	Age at wave 3	1.000	0.000
Age at wave 3	~~	Age at wave 4	0.912	0.000
Age at wave 4	~~	Age at wave 4	1.000	0.000

LHS = Left-hand side (of the structural equation); RHS = right-hand side. The path column indicates the type of relationship between the two variables, ~ indicates a regression, and ~~ indicates a correlation or covariance.

For peer review only

Supplementary Table 3. Bivariate associations of the latent growth curve model (1B) of blood pressure and cognitive function with control covariates.

LHS	Path	RHS	Std. Estimate	S.E.
Blood pressure level	~~	Blood pressure quadratic	0.264	0.095
Blood pressure slope	~~	Blood pressure quadratic	-0.929	0.018
Blood pressure level	~~	Blood pressure slope	-0.385	0.077
Cognitive function level	~~	Cognitive function slope	0.358	0.095
Fluid function level	~~	Fluid function slope	-0.682	0.504
Processing speed level	~~	Processing speed slope	-0.395	0.123
Memory ability level	~~	Memory ability slope	-0.455	0.092
Crystiallized ability level	~~	Crystiallized ability slope	-0.174	0.330
Blood pressure level	~~	Cognitive function level	-0.046	0.059
Blood pressure slope	~~	Cognitive function slope	-0.110	0.081
Blood pressure quadratic	~~	Cognitive function slope	0.168	0.092
Blood pressure level	~~	Cognitive function slope	0.063	0.057
Blood pressure slope	~~	Cognitive function level	0.038	0.090
Blood pressure quadratic	~~	Cognitive function level	-0.005	0.092
Cognitive function slope	~	Age 11 cognitive function	-0.011	0.088
Cognitive function slope	~	Education	-0.081	0.064
Cognitive function slope	~	APOEe4	-0.183	0.064
Cognitive function slope	~	Sex	0.111	0.055
Cognitive function slope	~	Sex x age 11 CF	0.027	0.066
Blood pressure quadratic	~	Age 11 cognitive function	-0.043	0.112
Blood pressure quadratic	~	Education	0.156	0.088
Blood pressure quadratic	~	Current smoker	-0.194	0.100
Blood pressure quadratic	~	Former Current smoker	-0.012	0.075

For peer review only

Blood pressure quadratic	~	Sex	0.171	0.061
Blood pressure quadratic	~	Sex x age 11 CF	-0.035	0.110
Blood pressure slope	~	Age 11 cognitive function	0.002	0.099
Blood pressure slope	~	Education	-0.140	0.075
Blood pressure slope	~	Current smoker	0.178	0.089
Blood pressure slope	~	Former Current smoker	0.014	0.072
Blood pressure slope	~	Sex	-0.073	0.049
Blood pressure slope	~	Sex x age 11 CF	0.054	0.102
Blood pressure level	~	Age 11 cognitive function	0.099	0.072
Blood pressure level	~	Education	-0.029	0.053
Blood pressure level	~	Current smoker	-0.104	0.040
Blood pressure level	~	Former Current smoker	0.011	0.047
Blood pressure level	~	Sex	-0.055	0.046
Blood pressure level	~	Sex x age 11 CF	-0.106	0.058
Cognitive function level	~	Age 11 cognitive function	0.741	0.035
Cognitive function level	~	Education	0.242	0.031
Cognitive function level	~	APOEe4	-0.083	0.027
Cognitive function level	~	Sex	-0.051	0.025
Cognitive function level	~	Sex x age 11 CF	0.011	0.051
Systolic pressure measurement 1, wave 1	~	Age at wave 1	-0.050	0.047
Systolic pressure measurement 1, wave 1	~	CVD history at wave 1	0.011	0.038
Systolic pressure measurement 1, wave 1	~	Stroke history at wave 1	0.037	0.047
Systolic pressure measurement 2, wave 1	~	Age at wave 1	-0.097	0.040
Systolic pressure measurement 2, wave 1	~	CVD history at wave 1	0.005	0.039
Systolic pressure measurement 2, wave 1	~	Stroke history at wave 1	0.063	0.049
Systolic pressure measurement 3, wave 1	~	Age at wave 1	-0.096	0.048
Systolic pressure measurement 3, wave 1	~	CVD history at wave 1	0.012	0.037
Systolic pressure measurement 3, wave 1	~	Stroke history at wave 1	0.061	0.048
Diastolic pressure measurement 1, wave 1	~	Age at wave 1	-0.103	0.038
Diastolic pressure measurement 1, wave 1	~	CVD history at wave 1	-0.050	0.045

For peer review only

Diastolic pressure measurement 1, wave 1	~	Stroke history at wave 1	0.028	0.029
Diastolic pressure measurement 2, wave 1	~	Age at wave 1	-0.079	0.044
Diastolic pressure measurement 2, wave 1	~	CVD history at wave 1	-0.012	0.045
Diastolic pressure measurement 2, wave 1	~	Stroke history at wave 1	0.030	0.028
Diastolic pressure measurement 3, wave 1	~	Age at wave 1	-0.102	0.037
Diastolic pressure measurement 3, wave 1	~	CVD history at wave 1	-0.041	0.044
Diastolic pressure measurement 3, wave 1	~	Stroke history at wave 1	0.035	0.027
Systolic pressure measurement 1, wave 2	~	Age at wave 2	-0.024	0.043
Systolic pressure measurement 1, wave 2	~	CVD history at wave 2	-0.122	0.037
Systolic pressure measurement 1, wave 2	~	Stroke history at wave 2	0.025	0.042
Systolic pressure measurement 2, wave 2	~	Age at wave 2	0.011	0.049
Systolic pressure measurement 2, wave 2	~	CVD history at wave 2	-0.045	0.035
Systolic pressure measurement 2, wave 2	~	Stroke history at wave 2	-0.014	0.045
Systolic pressure measurement 3, wave 2	~	Age at wave 2	0.026	0.046
Systolic pressure measurement 3, wave 2	~	CVD history at wave 2	-0.026	0.031
Systolic pressure measurement 3, wave 2	~	Stroke history at wave 2	-0.011	0.042
Diastolic pressure measurement 1, wave 2	~	Age at wave 2	0.048	0.043
Diastolic pressure measurement 1, wave 2	~	CVD history at wave 2	-0.053	0.053
Diastolic pressure measurement 1, wave 2	~	Stroke history at wave 2	-0.017	0.037
Diastolic pressure measurement 2, wave 2	~	Age at wave 2	0.053	0.041
Diastolic pressure measurement 2, wave 2	~	CVD history at wave 2	-0.051	0.051
Diastolic pressure measurement 2, wave 2	~	Stroke history at wave 2	-0.019	0.035
Diastolic pressure measurement 3, wave 2	~	Age at wave 2	0.042	0.042
Diastolic pressure measurement 3, wave 2	~	CVD history at wave 2	-0.037	0.053
Diastolic pressure measurement 3, wave 2	~	Stroke history at wave 2	-0.014	0.038
Systolic pressure measurement 1, wave 3	~	Age at wave 3	-0.101	0.048
Systolic pressure measurement 1, wave 3	~	CVD history at wave 3	-0.108	0.037
Systolic pressure measurement 1, wave 3	~	Stroke history at wave 3	-0.078	0.040
Systolic pressure measurement 2, wave 3	~	Age at wave 3	-0.092	0.048
Systolic pressure measurement 2, wave 3	~	CVD history at wave 3	-0.116	0.035

For peer review only

Systolic pressure measurement 2, wave 3	~	Stroke history at wave 3	-0.045	0.041
Systolic pressure measurement 3, wave 3	~	Age at wave 3	-0.103	0.049
Systolic pressure measurement 3, wave 3	~	CVD history at wave 3	-0.083	0.038
Systolic pressure measurement 3, wave 3	~	Stroke history at wave 3	-0.028	0.041
Diastolic pressure measurement 1, wave 3	~	Age at wave 3	0.043	0.041
Diastolic pressure measurement 1, wave 3	~	CVD history at wave 3	-0.055	0.056
Diastolic pressure measurement 1, wave 3	~	Stroke history at wave 3	-0.047	0.046
Diastolic pressure measurement 2, wave 3	~	Age at wave 3	0.060	0.043
Diastolic pressure measurement 2, wave 3	~	CVD history at wave 3	-0.033	0.042
Diastolic pressure measurement 2, wave 3	~	Stroke history at wave 3	-0.044	0.045
Diastolic pressure measurement 3, wave 3	~	Age at wave 3	0.026	0.044
Diastolic pressure measurement 3, wave 3	~	CVD history at wave 3	-0.049	0.053
Diastolic pressure measurement 3, wave 3	~	Stroke history at wave 3	-0.046	0.045
Systolic pressure measurement 1, wave 4	~	Age at wave 4	-0.062	0.031
Systolic pressure measurement 1, wave 4	~	CVD history at wave 4	0.013	0.034
Systolic pressure measurement 1, wave 4	~	Stroke history at wave 4	-0.082	0.037
Systolic pressure measurement 2, wave 4	~	Age at wave 4	-0.092	0.031
Systolic pressure measurement 2, wave 4	~	CVD history at wave 4	-0.008	0.034
Systolic pressure measurement 2, wave 4	~	Stroke history at wave 4	-0.029	0.040
Systolic pressure measurement 3, wave 4	~	Age at wave 4	-0.068	0.036
Systolic pressure measurement 3, wave 4	~	CVD history at wave 4	0.004	0.037
Systolic pressure measurement 3, wave 4	~	Stroke history at wave 4	-0.058	0.044
Diastolic pressure measurement 1, wave 4	~	Age at wave 4	0.056	0.046
Diastolic pressure measurement 1, wave 4	~	CVD history at wave 4	-0.061	0.038
Diastolic pressure measurement 1, wave 4	~	Stroke history at wave 4	-0.058	0.045
Diastolic pressure measurement 2, wave 4	~	Age at wave 4	0.039	0.043
Diastolic pressure measurement 2, wave 4	~	CVD history at wave 4	-0.049	0.042
Diastolic pressure measurement 2, wave 4	~	Stroke history at wave 4	-0.030	0.046
Diastolic pressure measurement 3, wave 4	~	Age at wave 4	0.024	0.045
Diastolic pressure measurement 3, wave 4	~	CVD history at wave 4	-0.042	0.044

For peer review only

Diastolic pressure measurement 3, wave 4	~	Stroke history at wave 4	-0.022	0.049
Systolic pressure measurement 1, wave 1	~~	Systolic pressure measurement 1, wave 1	0.129	0.015
Systolic pressure measurement 2, wave 1	~~	Systolic pressure measurement 2, wave 1	0.093	0.010
Systolic pressure measurement 3, wave 1	~~	Systolic pressure measurement 3, wave 1	0.097	0.013
Diastolic pressure measurement 1, wave 1	~~	Diastolic pressure measurement 1, wave 1	0.135	0.018
Diastolic pressure measurement 2, wave 1	~~	Diastolic pressure measurement 2, wave 1	0.099	0.013
Diastolic pressure measurement 3, wave 1	~~	Diastolic pressure measurement 3, wave 1	0.165	0.019
Systolic pressure measurement 1, wave 2	~~	Systolic pressure measurement 1, wave 2	0.197	0.016
Systolic pressure measurement 2, wave 2	~~	Systolic pressure measurement 2, wave 2	0.039	0.008
Systolic pressure measurement 3, wave 2	~~	Systolic pressure measurement 3, wave 2	0.082	0.009
Diastolic pressure measurement 1, wave 2	~~	Diastolic pressure measurement 1, wave 2	0.100	0.015
Diastolic pressure measurement 2, wave 2	~~	Diastolic pressure measurement 2, wave 2	0.075	0.011
Diastolic pressure measurement 3, wave 2	~~	Diastolic pressure measurement 3, wave 2	0.110	0.021
Systolic pressure measurement 1, wave 3	~~	Systolic pressure measurement 1, wave 3	0.141	0.018
Systolic pressure measurement 2, wave 3	~~	Systolic pressure measurement 2, wave 3	0.083	0.010
Systolic pressure measurement 3, wave 3	~~	Systolic pressure measurement 3, wave 3	0.085	0.012
Diastolic pressure measurement 1, wave 3	~~	Diastolic pressure measurement 1, wave 3	0.135	0.022
Diastolic pressure measurement 2, wave 3	~~	Diastolic pressure measurement 2, wave 3	0.146	0.034
Diastolic pressure measurement 3, wave 3	~~	Diastolic pressure measurement 3, wave 3	0.172	0.025
Systolic pressure measurement 1, wave 4	~~	Systolic pressure measurement 1, wave 4	0.118	0.013
Systolic pressure measurement 2, wave 4	~~	Systolic pressure measurement 2, wave 4	0.042	0.007
Systolic pressure measurement 3, wave 4	~~	Systolic pressure measurement 3, wave 4	0.104	0.013
Diastolic pressure measurement 1, wave 4	~~	Diastolic pressure measurement 1, wave 4	0.148	0.023
Diastolic pressure measurement 2, wave 4	~~	Diastolic pressure measurement 2, wave 4	0.102	0.018
Diastolic pressure measurement 3, wave 4	~~	Diastolic pressure measurement 3, wave 4	0.098	0.015
Blood pressure level	~~	Blood pressure level	0.976	0.015
Blood pressure slope	~~	Blood pressure slope	0.950	0.035
Blood pressure quadratic	~~	Blood pressure quadratic	0.922	0.045
Fluid function level	~~	Fluid function level	0.489	0.052
Processing speed level	~~	Processing speed level	0.545	0.051

For peer review only

Memory ability level	~~	Memory ability level	0.350	0.045
Crystiallized ability level	~~	Crystiallized ability level	0.325	0.037
Cognitive function level	~~	Cognitive function level	0.212	0.031
Fluid function slope	~~	Fluid function slope	0.158	0.249
Processing speed slope	~~	Processing speed slope	0.239	0.100
Memory ability slope	~~	Memory ability slope	0.409	0.104
Crystiallized ability slope	~~	Crystiallized ability slope	0.361	0.916
Cognitive function slope	~~	Cognitive function slope	0.944	0.026
Age 11 cognitive function	~~	Age 11 cognitive function	1.000	0.000
Age 11 cognitive function	~~	Education	0.445	0.000
Age 11 cognitive function	~~	APOEe4	-0.043	0.000
Age 11 cognitive function	~~	Sex	0.091	0.000
Age 11 cognitive function	~~	Sex x age 11 CF	0.659	0.000
Age 11 cognitive function	~~	Current smoker	0.012	0.000
Age 11 cognitive function	~~	Former Current smoker	-0.092	0.000
Age 11 cognitive function	~~	Age at wave 1	-0.090	0.000
Age 11 cognitive function	~~	CVD history at wave 1	-0.128	0.000
Age 11 cognitive function	~~	Stroke history at wave 1	0.001	0.000
Age 11 cognitive function	~~	Age at wave 2	-0.076	0.000
Age 11 cognitive function	~~	CVD history at wave 2	-0.097	0.000
Age 11 cognitive function	~~	Stroke history at wave 2	0.039	0.000
Age 11 cognitive function	~~	Age at wave 3	-0.081	0.000
Age 11 cognitive function	~~	CVD history at wave 3	-0.055	0.000
Age 11 cognitive function	~~	Stroke history at wave 3	0.019	0.000
Age 11 cognitive function	~~	Age at wave 4	-0.073	0.000
Age 11 cognitive function	~~	CVD history at wave 4	-0.043	0.000
Age 11 cognitive function	~~	Stroke history at wave 4	-0.027	0.000
Education	~~	Education	1.000	0.000
Education	~~	APOEe4	-0.028	0.000
Education	~~	Sex	0.025	0.000

For peer review only

Education	~~	Sex x age 11 CF	0.263	0.000
Education	~~	Current smoker	0.046	0.000
Education	~~	Former Current smoker	-0.091	0.000
Education	~~	Age at wave 1	-0.088	0.000
Education	~~	CVD history at wave 1	-0.056	0.000
Education	~~	Stroke history at wave 1	-0.035	0.000
Education	~~	Age at wave 2	-0.054	0.000
Education	~~	CVD history at wave 2	-0.043	0.000
Education	~~	Stroke history at wave 2	-0.006	0.000
Education	~~	Age at wave 3	-0.098	0.000
Education	~~	CVD history at wave 3	-0.042	0.000
Education	~~	Stroke history at wave 3	0.032	0.000
Education	~~	Age at wave 4	-0.075	0.000
Education	~~	CVD history at wave 4	-0.064	0.000
Education	~~	Stroke history at wave 4	0.033	0.000
APOEe4	~~	APOEe4	1.000	0.000
APOEe4	~~	Sex	-0.104	0.000
APOEe4	~~	Sex x age 11 CF	-0.013	0.000
APOEe4	~~	Current smoker	-0.111	0.000
APOEe4	~~	Former Current smoker	0.009	0.000
APOEe4	~~	Age at wave 1	0.048	0.000
APOEe4	~~	CVD history at wave 1	0.013	0.000
APOEe4	~~	Stroke history at wave 1	-0.047	0.000
APOEe4	~~	Age at wave 2	0.030	0.000
APOEe4	~~	CVD history at wave 2	0.017	0.000
APOEe4	~~	Stroke history at wave 2	-0.044	0.000
APOEe4	~~	Age at wave 3	0.055	0.000
APOEe4	~~	CVD history at wave 3	0.015	0.000
APOEe4	~~	Stroke history at wave 3	0.000	0.000
APOEe4	~~	Age at wave 4	0.033	0.000

For peer review only

APOEe4	~~	CVD history at wave 4	-0.001	0.000
APOEe4	~~	Stroke history at wave 4	0.000	0.000
Sex	~~	Sex	1.000	0.000
Sex	~~	Sex x age 11 CF	0.179	0.000
Sex	~~	Current smoker	0.078	0.000
Sex	~~	Former Current smoker	-0.123	0.000
Sex	~~	Age at wave 1	0.020	0.000
Sex	~~	CVD history at wave 1	-0.122	0.000
Sex	~~	Stroke history at wave 1	-0.021	0.000
Sex	~~	Age at wave 2	0.043	0.000
Sex	~~	CVD history at wave 2	-0.149	0.000
Sex	~~	Stroke history at wave 2	-0.024	0.000
Sex	~~	Age at wave 3	0.003	0.000
Sex	~~	CVD history at wave 3	-0.113	0.000
Sex	~~	Stroke history at wave 3	-0.038	0.000
Sex	~~	Age at wave 4	0.018	0.000
Sex	~~	CVD history at wave 4	-0.084	0.000
Sex	~~	Stroke history at wave 4	-0.047	0.000
Sex x age 11 CF	~~	Sex x age 11 CF	1.000	0.000
Sex x age 11 CF	~~	Current smoker	0.021	0.000
Sex x age 11 CF	~~	Former Current smoker	-0.066	0.000
Sex x age 11 CF	~~	Age at wave 1	-0.046	0.000
Sex x age 11 CF	~~	CVD history at wave 1	-0.104	0.000
Sex x age 11 CF	~~	Stroke history at wave 1	-0.018	0.000
Sex x age 11 CF	~~	Age at wave 2	-0.032	0.000
Sex x age 11 CF	~~	CVD history at wave 2	-0.082	0.000
Sex x age 11 CF	~~	Stroke history at wave 2	0.027	0.000
Sex x age 11 CF	~~	Age at wave 3	-0.068	0.000
Sex x age 11 CF	~~	CVD history at wave 3	-0.054	0.000
Sex x age 11 CF	~~	Stroke history at wave 3	0.016	0.000

For peer review only

Sex x age 11 CF	~~	Age at wave 4	-0.047	0.000
Sex x age 11 CF	~~	CVD history at wave 4	-0.073	0.000
Sex x age 11 CF	~~	Stroke history at wave 4	-0.041	0.000
Current smoker	~~	Current smoker	1.000	0.000
Current smoker	~~	Former Current smoker	-0.176	0.000
Current smoker	~~	Age at wave 1	0.056	0.000
Current smoker	~~	CVD history at wave 1	0.069	0.000
Current smoker	~~	Stroke history at wave 1	0.081	0.000
Current smoker	~~	Age at wave 2	0.049	0.000
Current smoker	~~	CVD history at wave 2	0.085	0.000
Current smoker	~~	Stroke history at wave 2	0.043	0.000
Current smoker	~~	Age at wave 3	0.026	0.000
Current smoker	~~	CVD history at wave 3	0.054	0.000
Current smoker	~~	Stroke history at wave 3	0.006	0.000
Current smoker	~~	Age at wave 4	0.058	0.000
Current smoker	~~	CVD history at wave 4	0.020	0.000
Current smoker	~~	Stroke history at wave 4	0.026	0.000
Former Current smoker	~~	Former Current smoker	1.000	0.000
Former Current smoker	~~	Age at wave 1	0.006	0.000
Former Current smoker	~~	CVD history at wave 1	0.109	0.000
Former Current smoker	~~	Stroke history at wave 1	0.004	0.000
Former Current smoker	~~	Age at wave 2	-0.025	0.000
Former Current smoker	~~	CVD history at wave 2	0.102	0.000
Former Current smoker	~~	Stroke history at wave 2	0.027	0.000
Former Current smoker	~~	Age at wave 3	-0.002	0.000
Former Current smoker	~~	CVD history at wave 3	0.080	0.000
Former Current smoker	~~	Stroke history at wave 3	0.076	0.000
Former Current smoker	~~	Age at wave 4	-0.011	0.000
Former Current smoker	~~	CVD history at wave 4	0.130	0.000
Former Current smoker	~~	Stroke history at wave 4	0.053	0.000

For peer review only

Age at wave 1	~~	Age at wave 1	1.000	0.000
Age at wave 1	~~	CVD history at wave 1	0.111	0.000
Age at wave 1	~~	Stroke history at wave 1	0.148	0.000
Age at wave 1	~~	Age at wave 2	0.949	0.000
Age at wave 1	~~	CVD history at wave 2	0.080	0.000
Age at wave 1	~~	Stroke history at wave 2	0.110	0.000
Age at wave 1	~~	Age at wave 3	0.944	0.000
Age at wave 1	~~	CVD history at wave 3	0.035	0.000
Age at wave 1	~~	Stroke history at wave 3	0.067	0.000
Age at wave 1	~~	Age at wave 4	0.893	0.000
Age at wave 1	~~	CVD history at wave 4	0.098	0.000
Age at wave 1	~~	Stroke history at wave 4	0.024	0.000
CVD history at wave 1	~~	CVD history at wave 1	1.000	0.000
CVD history at wave 1	~~	Stroke history at wave 1	0.010	0.000
CVD history at wave 1	~~	Age at wave 2	0.102	0.000
CVD history at wave 1	~~	CVD history at wave 2	0.779	0.000
CVD history at wave 1	~~	Stroke history at wave 2	-0.002	0.000
CVD history at wave 1	~~	Age at wave 3	0.105	0.000
CVD history at wave 1	~~	CVD history at wave 3	0.635	0.000
CVD history at wave 1	~~	Stroke history at wave 3	0.027	0.000
CVD history at wave 1	~~	Age at wave 4	0.085	0.000
CVD history at wave 1	~~	CVD history at wave 4	0.567	0.000
CVD history at wave 1	~~	Stroke history at wave 4	0.036	0.000
Stroke history at wave 1	~~	Stroke history at wave 1	1.000	0.000
Stroke history at wave 1	~~	Age at wave 2	0.148	0.000
Stroke history at wave 1	~~	CVD history at wave 2	-0.014	0.000
Stroke history at wave 1	~~	Stroke history at wave 2	0.761	0.000
Stroke history at wave 1	~~	Age at wave 3	0.132	0.000
Stroke history at wave 1	~~	CVD history at wave 3	-0.014	0.000
Stroke history at wave 1	~~	Stroke history at wave 3	0.570	0.000

For peer review only

Stroke history at wave 1	~~	Age at wave 4	0.132	0.000
Stroke history at wave 1	~~	CVD history at wave 4	-0.047	0.000
Stroke history at wave 1	~~	Stroke history at wave 4	0.511	0.000
Age at wave 2	~~	Age at wave 2	1.000	0.000
Age at wave 2	~~	CVD history at wave 2	0.056	0.000
Age at wave 2	~~	Stroke history at wave 2	0.111	0.000
Age at wave 2	~~	Age at wave 3	0.923	0.000
Age at wave 2	~~	CVD history at wave 3	0.020	0.000
Age at wave 2	~~	Stroke history at wave 3	0.060	0.000
Age at wave 2	~~	Age at wave 4	0.877	0.000
Age at wave 2	~~	CVD history at wave 4	0.067	0.000
Age at wave 2	~~	Stroke history at wave 4	0.013	0.000
CVD history at wave 2	~~	CVD history at wave 2	1.000	0.000
CVD history at wave 2	~~	Stroke history at wave 2	-0.014	0.000
CVD history at wave 2	~~	Age at wave 3	0.082	0.000
CVD history at wave 2	~~	CVD history at wave 3	0.738	0.000
CVD history at wave 2	~~	Stroke history at wave 3	0.014	0.000
CVD history at wave 2	~~	Age at wave 4	0.053	0.000
CVD history at wave 2	~~	CVD history at wave 4	0.684	0.000
CVD history at wave 2	~~	Stroke history at wave 4	0.000	0.000
Stroke history at wave 2	~~	Stroke history at wave 2	1.000	0.000
Stroke history at wave 2	~~	Age at wave 3	0.107	0.000
Stroke history at wave 2	~~	CVD history at wave 3	-0.027	0.000
Stroke history at wave 2	~~	Stroke history at wave 3	0.719	0.000
Stroke history at wave 2	~~	Age at wave 4	0.104	0.000
Stroke history at wave 2	~~	CVD history at wave 4	-0.039	0.000
Stroke history at wave 2	~~	Stroke history at wave 4	0.643	0.000
Age at wave 3	~~	Age at wave 3	1.000	0.000
Age at wave 3	~~	CVD history at wave 3	0.049	0.000
Age at wave 3	~~	Stroke history at wave 3	0.076	0.000

For peer review only

Age at wave 3	~~	Age at wave 4	0.902	0.000
Age at wave 3	~~	CVD history at wave 4	0.102	0.000
Age at wave 3	~~	Stroke history at wave 4	0.034	0.000
CVD history at wave 3	~~	CVD history at wave 3	1.000	0.000
CVD history at wave 3	~~	Stroke history at wave 3	0.015	0.000
CVD history at wave 3	~~	Age at wave 4	0.032	0.000
CVD history at wave 3	~~	CVD history at wave 4	0.783	0.000
CVD history at wave 3	~~	Stroke history at wave 4	-0.020	0.000
Stroke history at wave 3	~~	Stroke history at wave 3	1.000	0.000
Stroke history at wave 3	~~	Age at wave 4	0.065	0.000
Stroke history at wave 3	~~	CVD history at wave 4	0.012	0.000
Stroke history at wave 3	~~	Stroke history at wave 4	0.807	0.000
Age at wave 4	~~	Age at wave 4	1.000	0.000
Age at wave 4	~~	CVD history at wave 4	0.061	0.000
Age at wave 4	~~	Stroke history at wave 4	0.030	0.000
CVD history at wave 4	~~	CVD history at wave 4	1.000	0.000
CVD history at wave 4	~~	Stroke history at wave 4	-0.039	0.000
Stroke history at wave 4	~~	Stroke history at wave 4	1.000	0.000

LHS = Left-hand side (of the structural equation); RHS = right-hand side. The path column indicates the type of relationship between the two variables, ~ indicates a regression, and ~~ indicates a correlation or covariance.

For peer review only

STROBE 2007 (v4) Statement—Checklist of items that should be included in reports of *cohort studies*

Section/Topic	Item #	Recommendation	Reported on page #
**Title and abstract**	1	(*a*) Indicate the study's design with a commonly used term in the title or the abstract	1
		(*b*) Provide in the abstract an informative and balanced summary of what was done and what was found	2,3
**Introduction**			
Background/rationale	2	Explain the scientific background and rationale for the investigation being reported	5, 6
Objectives	3	State specific objectives, including any prespecified hypotheses	6
**Methods**			
Study design	4	Present key elements of study design early in the paper	6, 7
Setting	5	Describe the setting, locations, and relevant dates, including periods of recruitment, exposure, follow-up, and data collection	6, 7
Participants	6	(*a*) Give the eligibility criteria, and the sources and methods of selection of participants. Describe methods of follow-up	6, 7
		(*b*) For matched studies, give matching criteria and number of exposed and unexposed	NA
Variables	7	Clearly define all outcomes, exposures, predictors, potential confounders, and effect modifiers. Give diagnostic criteria, if applicable	7, 8
Data sources/ measurement	8*	For each variable of interest, give sources of data and details of methods of assessment (measurement). Describe comparability of assessment methods if there is more than one group	7, 8
Bias	9	Describe any efforts to address potential sources of bias	10
Study size	10	Explain how the study size was arrived at	6, 7
Quantitative variables	11	Explain how quantitative variables were handled in the analyses. If applicable, describe which groupings were chosen and why	9, 10
Statistical methods	12	(*a*) Describe all statistical methods, including those used to control for confounding	9, 10
		(*b*) Describe any methods used to examine subgroups and interactions	9, 10
		(*c*) Explain how missing data were addressed	10
		(*d*) If applicable, explain how loss to follow-up was addressed	10
		(*e*) Describe any sensitivity analyses	NA
**Results**			

For peer review only

Participants	13*	(a) Report numbers of individuals at each stage of study—eg numbers potentially eligible, examined for eligibility, confirmed eligible, included in the study, completing follow-up, and analysed	Table 1, pg 22
		(b) Give reasons for non-participation at each stage	7
		(c) Consider use of a flow diagram	NA
Descriptive data	14*	(a) Give characteristics of study participants (eg demographic, clinical, social) and information on exposures and potential confounders	Table 1, pg 22
		(b) Indicate number of participants with missing data for each variable of interest	Table 1, pg 22; SMs
		(c) Summarise follow-up time (eg, average and total amount)	7, 11
Outcome data	15*	Report numbers of outcome events or summary measures over time	Table 1, pg 22
Main results	16	(*a*) Give unadjusted estimates and, if applicable, confounder-adjusted estimates and their precision (eg, 95% confidence interval). Make clear which confounders were adjusted for and why they were included	11-14
		(*b*) Report category boundaries when continuous variables were categorized	NA
		(*c*) If relevant, consider translating estimates of relative risk into absolute risk for a meaningful time period	NA
Other analyses	17	Report other analyses done—eg analyses of subgroups and interactions, and sensitivity analyses	13-15
**Discussion**			
Key results	18	Summarise key results with reference to study objectives	15
**Limitations**			
Interpretation	20	Give a cautious overall interpretation of results considering objectives, limitations, multiplicity of analyses, results from similar studies, and other relevant evidence	15, 16
Generalisability	21	Discuss the generalisability (external validity) of the study results	16, 17
**Other information**			
Funding	22	Give the source of funding and the role of the funders for the present study and, if applicable, for the original study on which the present article is based	18

*Give information separately for cases and controls in case-control studies and, if applicable, for exposed and unexposed groups in cohort and cross-sectional studies.

Note: An Explanation and Elaboration article discusses each checklist item and gives methodological background and published examples of transparent reporting. The STROBE checklist is best used in conjunction with this article (freely available on the Web sites of PLoS Medicine at http://www.plosmedicine.org/, Annals of Internal Medicine at http://www.annals.org/, and Epidemiology at http://www.epidem.com/). Information on the STROBE Initiative is available at www.strobe-statement.org.

For peer review only

BMJ Open

Blood pressure and cognitive function across the eighth decade: a prospective study of the Lothian Birth Cohort of 1936

Journal:	*BMJ Open*
Manuscript ID	bmjopen-2019-033990.R1
Article Type:	Original research
Date Submitted by the Author:	26-May-2020
Complete List of Authors:	Altschul, Drew; The University of Edinburgh, Psychology<br>Starr, John; Royal Victoria Hospital,<br>Deary, Ian; University of Edinburgh, Department of Psychology
**Primary Subject Heading**:	Epidemiology
Secondary Subject Heading:	Geriatric medicine, Cardiovascular medicine, Public health, Mental health
Keywords:	EPIDEMIOLOGY, Old age psychiatry < PSYCHIATRY, GERIATRIC MEDICINE, Hypertension < CARDIOLOGY

SCHOLARONE™
Manuscripts

I, the Submitting Author has the right to grant and does grant on behalf of all authors of the Work (as defined in the below author licence), an exclusive licence and/or a non-exclusive licence for contributions from authors who are: i) UK Crown employees; ii) where BMJ has agreed a CC-BY licence shall apply, and/or iii) in accordance with the terms applicable for US Federal Government officers or employees acting as part of their official duties; on a worldwide, perpetual, irrevocable, royalty-free basis to BMJ Publishing Group Ltd ("BMJ") its licensees and where the relevant Journal is co-owned by BMJ to the co-owners of the Journal, to publish the Work in this journal and any other BMJ products and to exploit all rights, as set out in our licence.

The Submitting Author accepts and understands that any supply made under these terms is made by BMJ to the Submitting Author unless you are acting as an employee on behalf of your employer or a postgraduate student of an affiliated institution which is paying any applicable article publishing charge ("APC") for Open Access articles. Where the Submitting Author wishes to make the Work available on an Open Access basis (and intends to pay the relevant APC), the terms of reuse of such Open Access shall be governed by a Creative Commons licence – details of these licences and which Creative Commons licence will apply to this Work are set out in our licence referred to above.

Other than as permitted in any relevant BMJ Author's Self Archiving Policies, I confirm this Work has not been accepted for publication elsewhere, is not being considered for publication elsewhere and does not duplicate material already published. I confirm all authors consent to publication of this Work and authorise the granting of this licence.

For peer review only

Blood pressure and cognitive function across the eighth decade: a prospective study of the Lothian Birth Cohort of 1936

Running title: Blood pressure and cognitive function across the eighth decade

Drew M Altschul[1,2,3*], John M Starr[1,4], Ian J Deary[1,3]

1. Department of Psychology, The University of Edinburgh, Edinburgh, UK
2. Mental Health Data Science Scotland
3. Lothian Birth Cohorts group, The University of Edinburgh, Edinburgh, UK
4. Alzheimer Scotland Dementia Research Centre, The University of Edinburgh, Edinburgh, UK

* corresponding author: dmaltschul@gmail.com

Word count: 4458

Data elements: Figures 1 – 4; Tables 1, 2; Supplemental file 1

For peer review only - http://bmjopen.bmj.com/site/about/guidelines.xhtml

Abstract

Objectives

We investigated the associations among blood pressure and cognitive functions across the eighth decade, while accounting for anti-hypertensive medication and lifetime stability in cognitive function.

Design

Prospective cohort study.

Setting

This study used data from the Lothian Birth Cohort 1936 (LBC1936) study, which recruited participants living in the Lothian region of Scotland when aged 70 years, most of whom had completed an intelligence test at age 11 years.

Participants

1091 members of the LBC1936 with assessments of cognitive ability in childhood and older adulthood, and blood pressure measurements in older adulthood.

Primary and secondary outcome measures

Participants were followed up at ages 70, 73, 76, and 79, and latent growth curve models and linear mixed models were used to analyse both cognitive functions and blood pressure as primary outcomes.

Results

Blood pressure followed a quadratic trajectory in the eighth decade: on average blood pressure rose in the first waves and subsequently fell. Intercepts and trajectories were not associated between blood pressure and cognitive functions. Women with higher early-life cognitive function generally had lower blood pressure during the eighth decade. Being

For peer review only

For peer review only - http://bmjopen.bmj.com/site/about/guidelines.xhtml

prescribed anti-hypertensive medication was associated with lower blood pressure, but not with better cognitive function.

Conclusions

Our findings indicate that women with higher early-life cognitive function had lower later-life blood pressure. However, we did not find support for the hypothesis that rises in blood pressure and worse cognitive decline are associated with one another in the eighth decade.

Keywords: hypertension, old age psychiatry, geriatric medicine, epidemiology

For peer review only - http://bmjopen.bmj.com/site/about/guidelines.xhtml

Strengths and limitations of this study

- This study used direct blood pressure measurements to model a continuous blood pressure score, as well as anti-hypertensive medication data, which were used to adjust blood pressure measurements.
- This study had comprehensive tests of cognitive ability measured in both childhood and old age which allowed us to investigate whether childhood and old age cognitive ability are distinctly related to blood pressure.
- Latent growth curve modelling allowed us to evaluate whether changes in either blood pressure or cognitive functions have downstream associations with one another.
- Larger samples and longer follow-up times, both from earlier and later in life, are needed to understand the long-term relationships among blood pressure, anti-hypertensive treatment, and cognitive functions.

For peer review only

Introduction

Hypertension is a major issue in middle and older age populations.[1 2] Hypertension has been consistently linked to cardiovascular diseases such as coronary artery disease (CAD), heart failure, and stroke.[1 3] It is also a risk factor for neurocognitive conditions such as age-related cognitive decline, vascular dementia, and possibly Alzheimer's disease.[4] Accelerated cognitive decline is associated with lower well-being, higher morbidity and mortality and, as cognitive function worsens, the clinical conditions of mild cognitive impairment and dementia can develop.[5] Some of hypertension's negative impacts on cognitive function have likely causal pathways: hypertension disrupts cerebral blood vessel structure and function, and is associated with stroke in relevant white matter regions.[6] Deeper study of these relationships is warranted as hypertension, age-related cognitive decline, and dementia are on the rise around the world.[6 7]

A major objective of modern gerontology is to understand how age-related cognitive changes systematically differ between individuals and groups. Typically, hypertension is thought of as a risk factor for later life cognitive decline. In some samples of older people, having hypertension is associated with lower cognitive functioning and faster decline.[6 8 9] However, there is also evidence for the relationship operating in the opposite direction, i.e. that higher cognitive function from earlier in life is associated with having lower risk of developing hypertension[10] and experiencing hypertension-related stroke and coronary artery events later in life.[11] These latter findings are part of a field known as cognitive epidemiology, which has found that higher cognitive function in early-life is associated with lower risk of a number of physical and mental ailments later in life.[12-16] Reality is usually more complex than can be captured by a unidirectional association, as may be the case for hypertension and cognitive decline. Lower blood flow to the brain can result from hypotension, which can gradually lead to brain damage, damage that might cause further blood pressure dysregulation, and subsequently worse cerebral blood flow issues.[17]

For peer review only - http://bmjopen.bmj.com/site/about/guidelines.xhtml

Men are more likely to develop cardiovascular conditions than women,[18] a reason why men have been the subject of more intervention studies than women.[19] Nevertheless, cardiovascular disease is the leading cause of death in both women and men.[20] Some differences in hypertension are biologically-based in differences between men and women, e.g. through hormones and gene dosage from the sex chromosomes, where women's additional X chromosome makes more copies of X linked genes available for transcription. These differences are consistent across different countries and ethnic groups.[19] Additionally, traditional gender roles are associated with men behaving in ways (e.g., higher smoking rates) that increase their risk for physical health conditions, including hypertension.[21] Previous work on the cognitive epidemiology of hypertension, CAD, and stroke found significant interactions between sex and cognitive function in youth: individuals with higher cognitive function were at lower risk for hypertension, CAD, and stroke, and the associations were stronger in women.[11 22] Socioeconomic conditions are the major avenues whereby youth cognitive function is associated with circulatory conditions. Previous work has established such factors as education[11] and income[22] as potential mediators of this sex difference.

Some cognitive functions steadily decline in mean levels in older participants, while hypertension is a condition that becomes increasingly common with age and is related to cardiovascular health and cognitive impairment. In light of this, we tested two hypotheses regarding the relationships between cognitive functions and blood pressure in the present study. First, we hypothesized that the association between higher cognitive function at age 11 years and lower blood pressure in the eighth decade of life is stronger in women than it is in men.[11] Second, we tested the hypothesis that cognitive function and blood pressure will be reciprocally associated with each other across the eighth decade. We were able to test these hypotheses using multi-wave data from the Lothian Birth Cohort 1936 (LBC1936), a narrow-age cohort of over 1000 community-dwelling people, and longitudinal modelling of

For peer review only - http://bmjopen.bmj.com/site/about/guidelines.xhtml

four waves of data that were collected from age 70 to 79 years. LBC1936 provides cognitive function data from age 11, as well as other control variables, such as education, which has been implicated as a mediator in the relationship between cognitive function in youth and cardiovascular risk,[23-25] and a variety of related behavioral and physical health variables.

Method

Participants

The Lothian Birth Cohort 1936 (LBC1936) is a community-dwelling sample of 1091 initially healthy individuals. All were born in 1936 and were at school in Scotland on 4 June 1947, when most took part in a group-administered intelligence test: the Moray House Test (MHT) No. 12. They were followed up in four waves of one-to-one cognitive and health testing between 2004 and 2017, at mean ages 70 (N = 1091), 73 (N = 866), 76 (N = 697), and 79 (N = 550) years. Further details on the background, recruitment, attrition, and data collection procedures are available.[26 27] Participants provided written informed consent. Descriptive statistics for the individual participating in each wave of the study are presented in Table 1. Study completers only are described in supplementary table 1.

Cognitive functions

The Moray House Test No. 12 (MHT) is a broad cognitive ability test that includes word classification, proverbs, spatial items, and arithmetic. The test correlated about 0.8 at age 11 years with the Terman Merrill revision of the Stanford-Binet test, providing concurrent validity.[28]

In older age, cognitive function is known to show decline across multiple, but not all, subdomains.[29] We assessed, processing speed, memory, and fluid cognitive ability, all of which decline on average with age. We also assessed crystallised ability, which remains relatively stable in later life.[30] Fluid and crystallised abilities are the two divisions of

For peer review only

For peer review only - http://bmjopen.bmj.com/site/about/guidelines.xhtml

intelligence theorized by Cattell and Horn:[31] fluid intelligence is the inductive ability to use reasoning to solve novel problems, and crystallised intelligence is the ability to recall and apply already-known information. There are strong correlations among the subdomains, and because of this, cognitive function can be modelled hierarchically, with a general cognitive function factor that captures overall ability. Beneath that general factor, specific subdomains capture variation beyond general cognitive function.[32] The relationships among cognitive tests and subdomains are described in the Statistical Analyses section below.

Cognitive functions in waves 1–4 were assessed using 14 individually-administered cognitive tests at the same clinical research facility and using the same equipment and procedure for all four waves. The membership of any test within a particular subdomain was determined empirically, ultimately base on model fit from confirmatory factor analysis.[32-35] The tests are fully described and referenced in an open-access protocol article.[27] The fluid subdomain consisted of matrix reasoning and block design from the Wechsler Adult Intelligence Scale (WAIS),[36] and spatial span forward and backward from the Wechsler Memory Scale (WMS.)[37] Processing speed was measured through symbol search and digit symbol substitution from the WAIS, plus four-choice reaction time[38] and inspection time.[39] Memory was assessed using verbal paired associates and logical memory from the WMS,[37] and the letter–number sequencing and digit span backward subtests of the WAIS.[36] Crystallised ability was measured through the National Adult Reading Test,[40] Wechsler Test of Adult Reading,[41] and a phonemic verbal fluency test.[42]

Blood pressure

Blood pressure was measured six times at each wave, three times sitting and three times standing. Blood pressure measurements were divided into systolic pressure (maximum during one heartbeat) and diastolic pressure (minimum in between two heartbeats).

For peer review only

In addition to working with raw, unadjusted blood pressure measurements, we also wished to study a more natural course of blood pressure development that removed the potentially confounding influence of anti-hypertensive medication. Adjustments for anti-hypertensive medications were made to both systolic and diastolic blood pressure. 15 points were added to systolic pressure and 10 points were added to diastolic pressure[43] if an individual was recorded as taking antihypertensive medication, such as atenolol, lisinopril, bisoprolol, etc. at that wave.

Covariates

Sex and education were both included as covariates in all models. Education was recorded as the total number of years spent in formal education. Age 11 cognitive function was also included in all models, as was the interaction between sex and age 11 function.

Several behavioural and diagnosed health variable were included. Smoking status was dummy coded with two variables, current smokers and ex-smokers, with non-smokers as the reference group. Participants were genotyped for the presence of an *APOE* ε4 allele. Cardiovascular disease and stroke history were both recorded for each wave; each was coded as a binary variable, with a 1 indicating that the individual had a history of the disease. Testing dates varied slightly for every individual at every wave, so the exact age of each participant at testing was also recorded.

Statistical analyses

All four LBC1936 Waves were analysed in latent growth curve models (LGCMs), a structural equation modelling technique that allows the user simultaneously to define and analyse multiple latent and measured variables.[44] LGCMs make use of latent variables, which are constructed from multiple indicators, i.e. observed variables. Incorporating the

For peer review only - http://bmjopen.bmj.com/site/about/guidelines.xhtml
For peer review only

multiple measures of systolic and diastolic blood pressure allows the latent variables in LGCMs to account for measurement error,[45] and in LGCMs blood pressure can be modelled holistically and longitudinally with level (i.e. an intercept) and slope (i.e. a trajectory of change) parameters.

We modelled blood pressure variables using a hierarchical 'curve of factors' model (supplementary figure 1). At the bottom tier were individual systolic and diastolic readings (only sitting readings were used for LGCMs), grouped by wave. For each grouping, both a systolic and diastolic latent variable was specified, creating four latent variables each for systolic and diastolic pressure, one each per wave. From these latent systolic and diastolic variables, latent variables modelling growth were specified, in much the same way as with cognitive function. To model overall blood pressure level, each of the eight lower-order latent variables contributed its unit loaded value (i.e. x1). For blood pressure, the best fit models were those that included both linear and quadratic slope latent variables. To model linear slope of blood pressure, linearly increasing loadings were given as the average age at a given wave progressed (x0, x2.96, x6.72, x9.79). To model quadratic slope, quadratically increasing loadings were assigned (x0, x8.76, x45.16, x95.84).

We modelled cognitive function using a slightly different hierarchical 'factor of curves' model, previously established with these data.[32] For each cognitive test we modelled a level (essentially the age 70 baseline) and a linear slope (the change between age 70 and age 79, taking all four measurement occasions into account) with the same loadings as with the age-respective blood pressure variables. For each cognitive domain (see Cognitive Function above) a latent level and linear slope variable was correspondingly composed of the individual tests' latent level and latent slope variables. At the top of this cognitive hierarchy, latent levels and slopes for general cognitive function were formed from the level and slope variables of each cognitive domain. Similar models have been used and diagrams presented elsewhere[32 34] and a structural diagram to illustrate these measured and latent variables and the associations among them is presented in supplementary figure 2.

For peer review only - http://bmjopen.bmj.com/site/about/guidelines.xhtml

Put simply, BP level is analogous to BP at the study's beginning (age 70 years), BP slope is analogous to the linear magnitude at which BP increases or decreases over the following decade, and BP quadratic (slope) is analogous to the curvature of the change in BP over the following decade. Cognitive function level is analogous to cognitive function at age 70, and is known to be highly correlated with cognitive function at age 11,[32] demonstrating some stability of individual differences in cognitive function across the life course. Slopes of cognitive function represent the rate at which cognitive functions change (mostly decrease, in fact) over subsequent waves, which is otherwise known as cognitive decline.

Time invariant covariates that applied to all waves of data (sex, age 11 cognitive function, their interaction, years of education, and smoking history) were associated with the intercept and slope parameters for latent levels and slopes, including those for the domains of cognitive function, but not onto individual cognitive tests' levels or slopes. *APOE* ε4 status only applied to cognitive function latent variables. Individual systolic and diastolic BP measurements were associated with time varying covariates, i.e. characteristics of individual waves: age at testing, cardiovascular disease history, and stroke history. Latent Growth Curve Model 1A included the time invariant covariates sex, age 11 cognitive functions, their interaction, and education, as well as time varying age at date of assessment. Model 1B included these covariates as well as *APOE* ε4 status and time varying factors of smoking and disease history.

Model parameters were estimated using full-information maximum likelihood, i.e. all data were used for all participants, even individuals who did not complete all waves. Standard errors were calculated using the robust Ruber-White method, and *P*-values were computed using the Yuan-Bentler scaled test statistic.[46] The false-discovery rate correction for multiple testing was applied to the variables in each model that were not control covariates, which included the associations between the latent variables of cognitive function and its subdomains and BP, as well as associations from sex, age 11 cognitive function, and

For peer review only - http://bmjopen.bmj.com/site/about/guidelines.xhtml

their interaction. All analyses were conducted in the R programming language (version 3.3.2), using the 'lavaan' package for modelling.[47]

Patient and public involvement

LBC1936 participants were not involved in the development of any part of this study. The results will be disseminated to participants via a quarterly newsletter sent to LBC1936 participants.

Results

Modelling adjusted blood pressure

The best fit model of BP adjusted for medication was a good fit for these data (χ^2 = 2052.76, df = 259, P < 0.001, CFI = 0.931, RMSEA = 0.080). The correlations between the level, linear slope, and quadratic slope latent BP variables were as follows: $r_{level,slope}$ = -0.466 (z = -10.216, P < 0.001), $r_{level,quad}$ = 0.323 (z = 5.525, P < 0.001), $r_{slope,quad}$ = -0.466 (z = -90.460, P < 0.001).

Blood pressure scores for each wave were derived from the latent variables and illustrated in Figure 1. In both hypertensive and normotensive individuals, blood pressure scores were similar at each age except for a notable but unsurprising difference in magnitude: individuals with hypertension had much higher blood pressure than those who did not have hypertension. Both groups show a quadratic effect, blood pressure rose between ages 70 and 76, and then declined between age 76 and 79.

Adjusted blood pressure and cognitive function

The best fit model of both adjusted blood pressure and cognitive function included latent variables for level and linear slope (χ^2 = 8911.01, df = 4023, P < 0.001, CFI = 0.928,

For peer review only

For peer review only - http://bmjopen.bmj.com/site/about/guidelines.xhtml

RMSEA = 0.033). Models including a quadratic factor of cognitive function change either would not converge successfully or fit very poorly and could not be trusted to produce reliable estimates for cognitive function, and thus were not included in our models. Adjusted blood pressure was best modelled with a quadratic factor of change.

Overall, we found no associations between latent variables of cognitive function with those of blood pressure in our bivariate growth curve (Model 1A, Figure 2 & supplementary table 2). After correction for multiple comparisons, only three tested associations (out of 22) survived. First, a correlation between eighth decade cognitive function level and slope (r = 0.326, SE = 0.059, $P < 0.001$), which suggests that individuals with higher overall cognitive function have steeper cognitive decline over the eighth decade, likely because they have more functional ability to lose. Second, there was a regression path from age 11 cognitive function to eighth decade cognitive function level ($\beta = 0.693$, SE = 0.036, $P < 0.001$), which reproduces a well-known phenomenon: cognitive function shows substantial stability of individual differences across the life course,[48] and different measures taken at different times will still be strongly associated.

Third, a significant regression path was found from the interaction of age 11 cognitive function and sex to eighth decade blood pressure level ($\beta = -0.136$, SE = 0.050, $P = 0.047$). This interaction association indicates that women with higher cognitive function at age 11 have lower blood pressure in later life, and women with lower cognitive function have higher blood pressure. The opposite was true for men: males with higher cognitive function at age 11 had higher blood pressure and vice versa (Figure 3). No additional associations were found between any specific cognitive domains and blood pressure variables. All these tested associations are visualized in Figure 2.

Incorporating control variables into the LGCM (Model 1B, supplementary table 3) reduced the regression weight from the sex and age 11 cognitive function interaction to blood pressure level, so that it was no longer significant ($\beta = -0.106$, SE = 0.058, $P = 0.215$). The variable that most likely caused this change in the model was smoking behaviour;

For peer review only

For peer review only - http://bmjopen.bmj.com/site/about/guidelines.xhtml

specifically, being a current smoker at age 70 had an association with eighth decade blood pressure (β = -0.104, SE = 0.040, post-hoc uncorrected P = 0.009).

Unadjusted blood pressure and cognitive function

We also attempted to fit bivariate LGCMs of unadjusted blood pressure and cognitive function. These models were intended to be the same as the models discussed above, but without the blood pressure variables being adjusted when individuals were recorded as taking anti-hypertensive medication. These models would not converge, and we were unable to progress further with this analysis. In order to investigate the influence of medication further, we carried out cross-sectional and additional longitudinal analyses.

Cross-sectional correlations between cognitive functions and blood pressure scores across waves

Given the quadratic trajectory of blood pressure over time in these data, and earlier evidence suggesting that the association between cognitive function and blood pressure changes depending on age,[49] we examined correlations between cognitive function, both domains and general ability, and blood pressure scores, both with and without adjustment for anti-hypertensive medication. We carried out these correlations within wave, that is, we looked at correlations between age 70 cognitive function and age 70 blood pressure, age 73 cognitive function and age 73, blood pressure, and so on for ages 76 and 79.

The results of these analyses are shown in Figure 4 and supplementary table 4. Correlations were similar at all time points, although all correlations were also small; no 95% confidence intervals did not overlap with zero.

Diagnosis and treatment effects

For peer review only

For peer review only - http://bmjopen.bmj.com/site/about/guidelines.xhtml

In an effort to understand why we found no associations between cognitive function and blood pressure in the eighth decade, we examined the role of medication in post-hoc analyses. The majority of individuals diagnosed with hypertension in the LBC1936 were prescribed medication for the condition. Following from Figure 1, though individuals with hypertension would have had higher blood pressure were they not being treated, they are being treated, and their blood pressure appears to generally decline over the eighth decade.

We fit linear mixed effect models to explore the associations of diagnosis and medication with blood pressure. Using latent variables for blood pressure, we generated blood pressure scores at each wave, but, by contrast with the previous analyses, the scores were based on the raw, medication-unadjusted systolic and diastolic blood pressure measurements. Using these scores, we first fit a mixed model predicting blood pressure score from a 2-way model that included the wave of the study, whether an individual had a hypertension diagnosis at that wave, and the interaction of the two (Model 2A, Table 2). We found a significant main effect of wave on blood pressure score (β = -0.048, SE = 0.014, P < 0.001) and interaction between wave and hypertension diagnosis (β = -0.111, SE = 0.028, P < 0.001), suggesting that individuals who are given a hypertension diagnosis have decreasing blood pressure over time.

In a second mixed effects model we replaced hypertension diagnosis by wave with a single variable indicating if a hypertension diagnosis was given at any wave, i.e. if a participant was diagnosed with hypertension during the eighth decade. This operationalization of diagnosis may capture more information than individual wave diagnoses; for example, an individual who first reports a hypertension diagnosis at wave 3 might already have hypertension at wave 2, and even if they do not have hypertension at wave 1, their blood pressure is likely to be elevated as they will be on their way to developing clinical hypertension. By coding all this information in a single variable we reduce the number of comparisons in the models as well as potentially improving our operationalization. As a result (Model 2B, Table 2) we found similar associations as in model

For peer review only - http://bmjopen.bmj.com/site/about/guidelines.xhtml

2A, except that we also found a main effect of being diagnosed with hypertension at any wave, indicating that individuals with a diagnosis generally had higher blood pressure as we would expect.

We fit two additional linear mixed effects models to examine associations between hypertension diagnosis and cognitive function. Because later life cognitive function is strongly associated with early life cognitive function, we included age 11 cognitive function as an independent variable in our regression models, and included all 2-way and 3-way interactions with wave and whether an individual was diagnosed with hypertension.

These models (Models 3A & 3B, Table 2) reproduced several established effects[32 48]. Age 11 cognitive function is associated with cognitive function scores in the eighth decade (β = 0.680, SE = 0.023, $P < 0.001$). Cognitive function declines across successive waves (β = -0.154, SE = 0.007, $P < 0.001$). Individuals with higher cognitive function early in life have more ability to lose, so declines are greater for these individuals across time (β = -0.039, SE = 0.014, $P = 0.006$).

In the second model of this set of models (Model 3B, Table 2), there was also a main effect of being diagnosed with hypertension at any wave and overall cognitive function level (β = -0.105, SE = 0.029, $P < 0.001$). Having a hypertension diagnosis was associated with lower cognitive function, which could not be fully explained by cognitive function scores from earlier in life. Put another way, being diagnosed with hypertension was not associated with improved cognitive function nor could diagnosis account for for the negative associations between blood pressure and cognitive decline. In previous models (Table 2) being diagnosed with hypertension was associated with reduced blood pressure over time. Therefore, whereas hypertension diagnoses and anti-hypertensive medication appear to successfully treat high blood pressure in this cohort, they do not appear to impact associated cognitive deficiencies.

For peer review only

For peer review only - http://bmjopen.bmj.com/site/about/guidelines.xhtml

Discussion

Our results show that sex and cognitive function from early-life interact to predict blood pressure level during the eighth decade. Women with higher cognitive function are less likely than higher cognitively functioning men to have higher blood pressure, but the opposite is also true: lower cognitive function women are more likely to have higher blood pressure than similarly functioning men (Figure 3). This finding provides mixed support for our first hypothesis and the existing supporting literature,[19 22] as the effect was not robust to the inclusion of covariates. Some of this association can be explained by health behaviors and other health conditions, some of which stand out. Notably, whether one is a smoker or not appeared to explain some of the same variation as the sex by cognitive function interaction.

We did not find compelling evidence to support our second hypothesis. There were no associations between high blood pressure and cognitive function, either in level or trajectory of function, nor were any other health or sociodemographic variables associated with change in either blood pressure or cognitive function in the eighth decade. As a null result, this is a difficult finding to interpret. It may be that we did not have sufficient power to detect such associations in our sample, although nearly 40% of the sample had been diagnosed with hypertension by the initial wave at age 70. Another possibility is that cognitive function is most impacted by side effects of hypertension before the eighth decade: our findings tentatively support this, as individuals with a hypertension diagnosis tended to have lower cognitive function, below what we would expect from baseline assessments at age 11.

Another possibility is that anti-hypertensive medications were effective at reducing hypertensive symptoms, which includes the connections between cognitive function and hypertension. Our findings suggest that participants of this sample who are diagnosed with hypertension take their medication, and this had a significant, ameliorative effect on their raised blood pressure. However, we found no evidence that either medication or behavioral

For peer review only - http://bmjopen.bmj.com/site/about/guidelines.xhtml

changes that might result from being diagnosed with hypertension have a positive association with subsequent cognitive function. In other words, treatment from antihypertensive medication in the eighth decade did not impact age or hypertension-related cognitive decline in this sample. These findings are consistent with large trials that have found no significant link between controlling blood pressure and dementia incidence.[50 51]

There is existing evidence for the importance of lifestyle factors in explaining the associations between cognitive function and physical health. In the National Longitudinal Study of Youth 1979, higher cognitive function women were less likely to be given a hypertension diagnosis,[22] an interaction that closely matches our initial finding in the LBC1936. However, this effect was explained by income differences. The Aberdeen Children of the 1950s cohort yielded results that were also similar to ours; specifically, associations between childhood cognitive function and both stroke and coronary artery events were present in both sexes, but stronger in women.[11] However, in their analyses, the sex by cognitive function interaction effects on stroke and CAD outcomes could be accounted for by education. In both of these cases, there were socioeconomic factors that seemed to drive the interaction between early life cognitive function and sex, whereas we found that behaviors, specifically smoking, seemed to explain some of the interaction. These samples vary in age and location, so chronological and geographic-cultural cohort differences might explain the discrepancies.[52 53]

The present study is limited by a non-trivial proportion of missing data, particularly from individuals who died over the course of the study, or were too frail or otherwise unwilling to continue participating. Moreover, our analytic sample was more affluent than the average population, and may thus limit generalizability. The diagnoses and medications analyzed in the present study were self-reported and we were not able to cross-reference these reports with any physician records. Although we took steps to treat these variables conservatively in our analyses, self-reported diagnoses of hypertension tend to have lower validity than those drawn from medical records.[54]

For peer review only

Conclusions

In general, our results are consistent with previous work that has indicated that the effects of lower childhood cognitive function on hypertension are stronger and more consistent in women.[11 22] Despite there being known connections between high blood pressure and cognitive impairment, we did not find any evidence for this in the eighth decade. It appears that any cognitive lowering associated with hypertension may occur earlier in life. Future research ought to corroborate this and, in general, investigate the critical periods when hypertension might be detrimental to cognitive health.

For peer review only

For peer review only - http://bmjopen.bmj.com/site/about/guidelines.xhtml

Contributors: DMA discussed and planned the study and analyses, analysed the data, interpreted the data and drafted the initial manuscript. JMS discussed and planned the study and analyses, interpreted the data and contributed to the manuscript. IJD discussed and planned the study and analyses, interpreted the data and contributed to the manuscript.

Competing interests: The authors declare that they have no conflicts of interest.

Funding statement: The LBC1936 data were collected using a Research Into Ageing programme grant; this research continues as part of the Age UK-funded Disconnected Mind project. DMA, JMS, and IJD were members of The University of Edinburgh Centre for Cognitive Ageing and Cognitive Epidemiology, which was funded by the Biotechnology and Biological Sciences Research Council and Medical Research Council (MR/K026992/1). DMA is also funded by an MRC Mental Health Data Pathfinder award (MC_PC_17209).

Ethics approval: Ethics permissions were obtained from the Multicentre Research Ethics Committee for Scotland (wave 1, MREC/01/0/56), the Lothian Research Ethics Committee (wave 1, LREC/2003/2/29), and the Scotland a Research Ethics Committee (waves 2–4, 07/MRE00/58).

Data sharing statement: Lothian Birth Cohort 1936 data can be requested from the Lothian Birth Cohort 1936 research team, following completion of a data request application. More information can be found online (http://www.lothianbirthcohort.ed.ac.uk/content/collaboration).

Acknowledgements

The LBC1936 study is funded by Age UK (Disconnected Mind project). We thank the Scottish Council for Research in Education for allowing access to the SMS1947. We thank the LBC1936 Study participants and research team members.

For peer review only

For peer review only - http://bmjopen.bmj.com/site/about/guidelines.xhtml

References

1. Staessen JA, Gasowski J, Wang JG, et al. Risks of untreated and treated isolated systolic hypertension in the elderly: meta-analysis of outcome trials. *The Lancet* 2000;355(9207):865-72.
2. Beckett NS, Peters R, Fletcher AE, et al. Treatment of hypertension in patients 80 years of age or older. *New Engl J Med* 2008;358(18):1887-98.
3. McInnes GT. Hypertension and coronary artery disease: cause and effect. *Journal of Hypertension* 1995;13:S49-S56.
4. Barnes DE, Yaffe K. The projected effect of risk factor reduction on Alzheimer's disease prevalence. *The Lancet Neurology* 2011;10(9):819-28.
5. Salthouse T. Consequences of age-related cognitive declines. *Annual review of psychology* 2012;63:201-26.
6. Iadecola C, Yaffe K, Biller J, et al. Impact of hypertension on cognitive function: a scientific statement from the American Heart Association. *Hypertension* 2016;68(6):e67-e94.
7. Matthews F, Stephan B, Robinson L, et al. A two decade dementia incidence comparison from the Cognitive Function and Ageing Studies I and II. *Nature communications* 2016;7
8. Hughes TM, Sink KM. Hypertension and its role in cognitive function: current evidence and challenges for the future. *American journal of hypertension* 2015;29(2):149-57.
9. Birns J, Kalra L. Cognitive function and hypertension. *Journal of human hypertension* 2009;23(2):86.
10. Wraw C, Deary IJ, Gale CR, et al. Intelligence in youth and health at age 50. *Intelligence* 2015;53:23-32.
11. Lawlor DA, Batty GD, Clark H, et al. Association of childhood intelligence with risk of coronary heart disease and stroke: findings from the Aberdeen Children of the 1950s cohort study. *Eur J Epidemiol* 2008;23(10):695-706.
12. Calvin CM, Batty GD, Deary IJ. Cognitive epidemiology. *The Wiley-Blackwell Handbook of Individual Differences* 2011:427-60.
13. Batty GD, Deary IJ, Gottfredson LS. Premorbid (early life) IQ and later mortality risk: Systematic review. *Ann Epidemiol* 2007;17:278-88.
14. Deary IJ. Cognitive epidemiology: Its rise, its current issues, and its challenges. *Personality and individual differences* 2010;49(4):337-43.
15. Deary IJ. Looking for 'system integrity'in cognitive epidemiology. *Gerontology* 2012;58(6):545-53.
16. Lubinski D. Cognitive epidemiology: With emphasis on untangling cognitive ability and socioeconomic status. *Intelligence* 2009;37(6):625-33.
17. Sabayan B, Westendorp RG. Blood pressure control and cognitive impairment—why low is not always better. *JAMA internal medicine* 2015;175(4):586-87.
18. Jousilahti P, Vartiainen E, Tuomilehto J, et al. Sex, age, cardiovascular risk factors, and coronary heart disease. *Circulation* 1999;99(9):1165-72.
19. Sandberg K, Ji H. Sex differences in primary hypertension. *Biology of sex differences* 2012;3(1):7.
20. Bots SH, Peters SA, Woodward M. Sex differences in coronary heart disease and stroke mortality: a global assessment of the effect of ageing between 1980 and 2010. *BMJ global health* 2017;2(2):e000298.
21. Courtenay WH. Constructions of masculinity and their influence on men's well-being: a theory of gender and health. *Social science & medicine* 2000;50(10):1385-401.
22. Altschul DM, Wraw C, Der G, et al. Hypertension development by midlife and the roles of pre-morbid cognitive function, sex, and their interaction. *Hypertension* 2019
23. Hagger-Johnson G, Mõttus R, Craig LC, et al. Pathways from childhood intelligence and socioeconomic status to late-life cardiovascular disease risk. *Health Psychol* 2012;31(4):403.

For peer review only

For peer review only - http://bmjopen.bmj.com/site/about/guidelines.xhtml

24. Leng B, Jin Y, Li G, et al. Socioeconomic status and hypertension: a meta-analysis. *Journal of hypertension* 2015;33(2):221-29.

25. Backholer K, Peters SAE, Bots SH, et al. Sex differences in the relationship between socioeconomic status and cardiovascular disease: a systematic review and meta-analysis. *J Epidemiol Community Health* 2017;71(6):550-57. doi: 10.1136/jech-2016-207890

26. Taylor AM, Pattie A, Deary IJ. Cohort Profile Update: The Lothian Birth Cohorts of 1921 and 1936. *Int J Epidemiol* 2018;47(4):1042-42r. doi: https://doi.org/10.1093/ije/dyy022

27. Deary IJ, Gow AJ, Taylor MD, et al. The Lothian Birth Cohort 1936: a study to examine influences on cognitive ageing from age 11 to age 70 and beyond. *BMC Geriatr* 2007;7(1):28.

28. Deary IJ, Whalley LJ, Starr JM. A lifetime of intelligence: Follow-up studies of the Scottish mental surveys of 1932 and 1947: American Psychological Association 2009.

29. Verhaeghen P, Salthouse TA. Meta-analyses of age–cognition relations in adulthood: Estimates of linear and nonlinear age effects and structural models. *Psychological bulletin* 1997;122(3):231.

30. Horn JL, Cattell RB. Age differences in fluid and crystallized intelligence. *Acta psychologica* 1967;26:107-29.

31. Cattell RB. Theory of fluid and crystallized intelligence: A critical experiment. *Journal of educational psychology* 1963;54(1):1.

32. Ritchie SJ, Tucker-Drob EM, Cox SR, et al. Predictors of ageing-related decline across multiple cognitive functions. *Intelligence* 2016;59:115-26. doi: https://doi.org/10.1016/j.intell.2016.08.007

33. Tucker-Drob EM, Briley DA, Starr JM, et al. Structure and correlates of cognitive aging in a narrow age cohort. *Psychology and Aging* 2014;29(2):236.

34. Altschul DM, Starr JM, Deary IJ. Cognitive function in early and later life is associated with blood glucose in older individuals: analysis of the Lothian Birth Cohort of 1936. *Diabetologia* 2018;61(9):1946-55. doi: https://doi.org/10.1007/s00125-018-4645-8

35. Altschul DM, Deary IJ. Playing analog games is associated with reduced declines in cognitive function: a 68 year longitudinal cohort study. *The Journals of Gerontology: Series B* 2019 doi: 10.1093/geronb/gbz149

36. Wechsler D. Adult intelligence scale. *New York* 1997;21

37. Wechsler D. Wechsler memory scale. 1945

38. Deary IJ, Der G, Ford G. Reaction times and intelligence differences: A population-based cohort study. *Intelligence* 2001;29:389–99.

39. Deary IJ, Johnson W, Starr JM. Are processing speed tasks biomarkers of cognitive aging? *Psychology and aging* 2010;25(1):219. doi: http://dx.doi.org/10.1037/a0017750

40. Nelson HE, Willison J. National adult reading test (NART): Nfer-Nelson Windsor 1991.

41. Wechsler D. Wechsler Test of Adult Reading: WTAR: Psychological Corporation 2001.

42. Lezak MD. Neuropsychological assessment: Oxford University Press, USA 2004.

43. Tobin MD, Sheehan NA, Scurrah KJ, et al. Adjusting for treatment effects in studies of quantitative traits: antihypertensive therapy and systolic blood pressure. *Stat Med* 2005;24(19):2911-35. doi: 10.1002/sim.2165

44. Preacher KJ. Latent growth curve modeling: Sage 2008.

45. Fontaine KR, Redden DT, Wang C, et al. Years of life lost due to obesity. *JAMA* 2003;289(2):187-93. doi: 10.1001/jama.289.2.187

46. Yuan K-H, Bentler PM. Mean and covariance structure analysis: Theoretical and practical improvements. *Journal of the American statistical association* 1997;92(438):767-74.

47. Rosseel Y. lavaan: an R package for structural equation modeling and more Version 0.4-9 (BETA): Ghent University, 2011.

48. Deary IJ, Pattie A, Starr JM. The stability of intelligence from age 11 to age 90 years: the Lothian birth cohort of 1921. *Psychol Sci* 2013;24(12):2361-68.

For peer review only - http://bmjopen.bmj.com/site/about/guidelines.xhtml

49. Euser SM, Van Bemmel T, Schram MT, et al. The effect of age on the association between blood pressure and cognitive function later in life. *J Am Geriatr Soc* 2009;57(7):1232-37.
50. Williamson JD, Pajewski NM, Auchus AP, et al. Effect of intensive vs standard blood pressure control on probable dementia: a randomized clinical trial. *Jama* 2019;321(6):553-61.
51. Peters R, Beckett N, Forette F, et al. Incident dementia and blood pressure lowering in the Hypertension in the Very Elderly Trial cognitive function assessment (HYVET-COG): a double-blind, placebo controlled trial. *The Lancet Neurology* 2008;7(8):683-89.
52. Tucker-Drob EM, Bates TC. Large cross-national differences in gene× socioeconomic status interaction on intelligence. *Psychol Sci* 2016;27(2):138-49.
53. Allender S, Scarborough P, O'Flaherty M, et al. Patterns of coronary heart disease mortality over the 20 th century in England and Wales: Possible plateaus in the rate of decline. *BMC Public Health* 2008;8(1):148.
54. Gonçalves VS, Andrade KR, Carvalho K, et al. Accuracy of self-reported hypertension: a systematic review and meta-analysis. *Journal of hypertension* 2018;36(5):970-78.

For peer review only

Table 1. Descriptive statistics for cognitive, demographic, and clinical variables.

	Wave 1	Wave 2	Wave 3	Wave 4
Total	1091	866	697	550
Female	543 (49.8%)	418 (48.3%)	337 (48.4%)	275 (50.0%)
Ever had high BP	433 (39.7%)	425 (49.1%)	378 (54.2%)	317 (57.6%)
Ever had CVD	268 (24.6%)	250 (28.9%)	236 (33.95)	204 (37.1%)
Ever had stroke	54 (4.95%)	55 (6.35%)	73 (10.5%)	69 (12.5%)
Ever had diabetes	91 (8.34%)	95 (11.0%)	82 (11.8%)	71 (13.0%)
Current smoker	125 (11.5%)	73 (8.43%)	44 (6.31%)	12 (3.81%)
Ex-smoker	465 (42.6%)	378 (43.6%)	293 (42.0%)	232 (42.2%)
Average sitting systolic BP	149.6 (19.04)	148.7 (18.83)	148.1 (19.31)	145.1 (18.8)
Average sitting diastolic BP	81.3 (10.14)	78.0 (9.81)	78.9 (10.3)	76.8 (10.1)
Years of education	10.7 (1.13)	10.8 (1.14)	10.8 (1.14)	10.9 (1.18)
Age 11 IQ	100.0 (14.99)	100.7 (15.3)	101.5 (15.3)	101.9 (15.3)
Matrix Reasoning*	13.5 (5.13)	13.2 (4.96)	13.0 (4.91)	12.9 (5.03)
Block Design*	33.8 (10.3)	33.6 (10.1)	32.2 (9.95)	31.2 (9.63)
Spatial Span*	7.04 (1.74)	7.06 (1.61)	7.05 (1.59)	6.74 (1.60)
NART†	34.5 (8.15)	34.4 (8.18)	35.0 (8.03)	35.6 (8.19)
WTAR†	41.0 (7.17)	41.0 (6.97)	41.1 (7.02)	41.6 (7.03)
Verbal Fluency†	42.4 (12.5)	43.2 (12.9)	42.9 (12.8)	43.6 (13.3)
Logical Memory‡	71.5 (18.0)	74.3 (17.9)	74.6 (19.2)	72.7 (20.4)
VPA‡	26.4 (9.13)	27.2 (9.46)	26.4 (9.56)	27.1 (9.55)
Digit Span‡	7.7 (2.26)	7.81 (2.29)	7.77 (2.37)	7.56 (2.18)
LNS‡	10.9 (3.16)	10.9 (3.08)	10.5 (2.99)	10.1 (2.89)
Symbol Search§	24.7 (6.39)	24.6 (6.18)	24.6 (6.46)	22.7 (6.63)
Digit-Symbol Coding§	56.6 (12.9)	56.4 (12.3)	53.8 (12.9)	51.2 (13.0)
Inspection Time§	112 (11.0)	111 (11.8)	110 (12.5)	107 (13.6)
Reaction Time§	0.64 (0.09)	0.65 (0.09)	0.68 (0.10)	0.71 (0.11)
MMSE	28.8 (1.43)	28.8 (1.42)	28.6 (1.70)	28.48 (2.16)

CVD, cardiovascular disease; BP, blood pressure; NART, National Adult Reading Test; WTAR, Wechsler Test of Adult Reading; VPA, Visual Paired Associates; LNS, Letter-Number Sequencing; MMSE, Mini-Mental State Examination; T2D, individuals with type 2 diabetes; T2D was defined as self-reported physician diagnosis of diabetes. * part of the fluid ability domain, † part of the crystallized ability domain, ‡ part of the memory domain, § part of the processing speed domain.

For peer review only - http://bmjopen.bmj.com/site/about/guidelines.xhtml

Table 2. Linear mixed effects regression models of blood pressure and cognitive function scores predicted by antihypertensive treatment over time.

Variable	Std Estimate	SE	Wald χ2	*P*
Predicting blood pressure score - Model 2A				
Wave	-0.048	0.014	11.854	< 0.001
Did the individual have HT at this wave?	-0.031	0.023	0.339	0.560
Wave x having HT	-0.111	0.028	15.370	< 0.001
Predicting blood pressure score - Model 2B				
Wave	-0.086	0.015	20.929	< 0.001
Did the individual have HT at any wave?	0.170	0.031	35.162	< 0.001
Wave x having HT	-0.208	0.032	43.536	< 0.001
Predicting cognitive function score - Model 3A				
Wave	-0.154	0.007	490.085	< 0.001
Did the individual have HT at **this** wave?	-0.008	0.013	0.552	0.458
Age 11 cognitive function	0.680	0.023	945.862	< 0.001
Wave x having HT	0.006	0.015	0.148	0.701
Wave x age 11 cognitive function	-0.039	0.014	7.512	0.006
Having HT x age 11 cognitive function	-0.037	0.028	1.751	0.186
Wave x having HT x age 11 function	-0.003	0.029	0.009	0.923
Predicting cognitive function score - Model 3B				
Wave	-0.157	0.007	517.451	< 0.001
Did the individual have HT at **any** wave?	-0.105	0.029	13.230	< 0.001
Age 11 cognitive function	0.678	0.025	754.084	< 0.001
Wave x having HT	-0.007	0.015	0.284	0.594
Wave x age 11 cognitive function	-0.041	0.015	7.747	0.005
Having HT x age 11 cognitive function	0.020	0.054	0.165	0.685
Wave x having HT x age 11 function	-0.014	0.029	0.216	0.642

For peer review only - http://bmjopen.bmj.com/site/about/guidelines.xhtml

Figure 1. Blood pressure trajectories in the eighth decade for individuals with and without hypertension diagnoses. Only study completers, those participants who were present at every wave of the study, were included in this plot. All study participants were used in the statistical analyses. Blood pressure score was derived from the latent variable that represent blood pressure in individual waves. Blood pressure score is a standardized, unitless measure of overall blood pressure magnitude; A score of 0 indicates the individual is in line with average blood pressure, and 1 would indicate that an individual was 1SD higher blood pressure at that time. The upper green line illustrates individuals diagnosed with hypertension at any wave (n = 337) and the lower red line illustrates normotensive individuals (n = 204). The shaded areas represent 95% confidence regions.

For peer review only

Figure 2. Path diagram displaying the statistical tests in a latent growth curve model of cognitive function and blood pressure. Circles represent latent variables and squares represent measured variables. Double arrowed lines represent correlations. Single arrowed lines (on the left side) represent regressions. Coefficients and SEs are the result of model 1A; numbers printed in bold indicate coefficients that were statistically significant after correction for multiple comparisons.

For peer review only

For peer review only - http://bmjopen.bmj.com/site/about/guidelines.xhtml

Figure 3. Blood pressure in older age and its relationship with cognitive function at age 11 years, sex, and their interaction. Blood pressure score was derived from the latent variable that represent blood pressure in individual waves. The green dots and line represent women, and the red dots and line represent men. The shaded areas represent 95% confidence regions.

For peer review only

Figure 4. Pearson correlations between general and subdomain cognitive function scores and blood pressure scores within each wave of the Lothian Birth Cohort. Wave is indicated across the top of each column; the left side of each pane represents correlations among data without adjustment for anti-hypertensive treatment, and the right side of each pane represents correlations among data with adjustment for anti-hypertensive treatment. Error bars are 95% confidence intervals around the correlation coefficient.

For peer review only

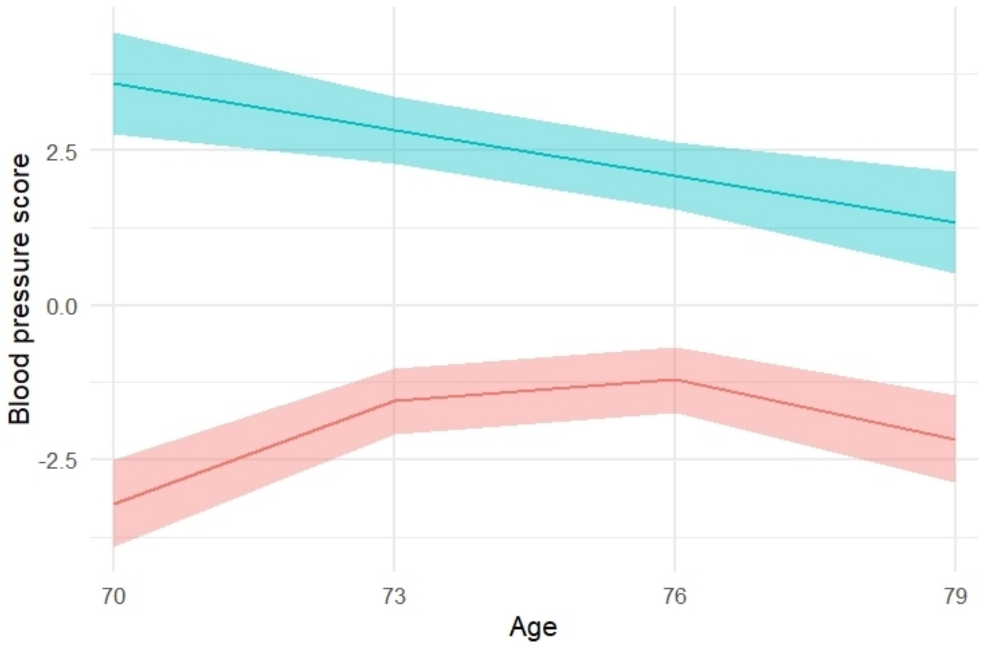

Figure 1. Blood pressure trajectories in the eighth decade for individuals with and without hypertension diagnoses. Only study completers, those participants who were present at every wave of the study, were included in this plot. All study participants were used in the statistical analyses. Blood pressure score was derived from the latent variable that represent blood pressure in individual waves. Blood pressure score is a standardized, unitless measure of overall blood pressure magnitude; A score of 0 indicates the individual is in line with average blood pressure, and 1 would indicate that an individual was 1SD higher blood pressure at that time. The upper green line illustrates individuals diagnosed with hypertension at any wave (n = 337) and the lower red line illustrates normotensive individuals (n = 204). The shaded areas represent 95% confidence regions.

63x41mm (300 x 300 DPI)

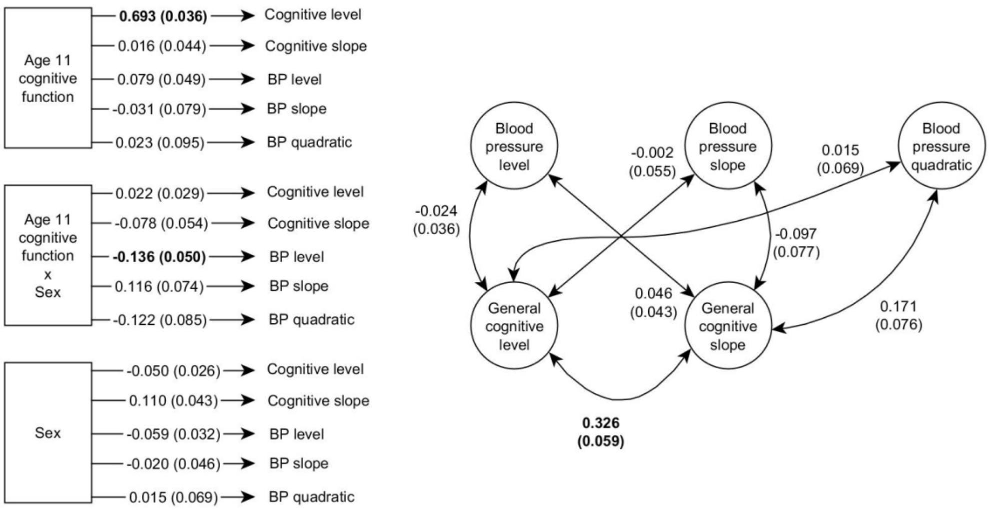

Figure 2. Path diagram displaying the statistical tests in a latent growth curve model of cognitive function and blood pressure. Circles represent latent variables and squares represent measured variables. Double arrowed lines represent correlations. Single arrowed lines (on the left side) represent regressions. Coefficients and SEs are the result of model 1A; numbers printed in bold indicate coefficients that were statistically significant after correction for multiple comparisons.

136x73mm (300 x 300 DPI)

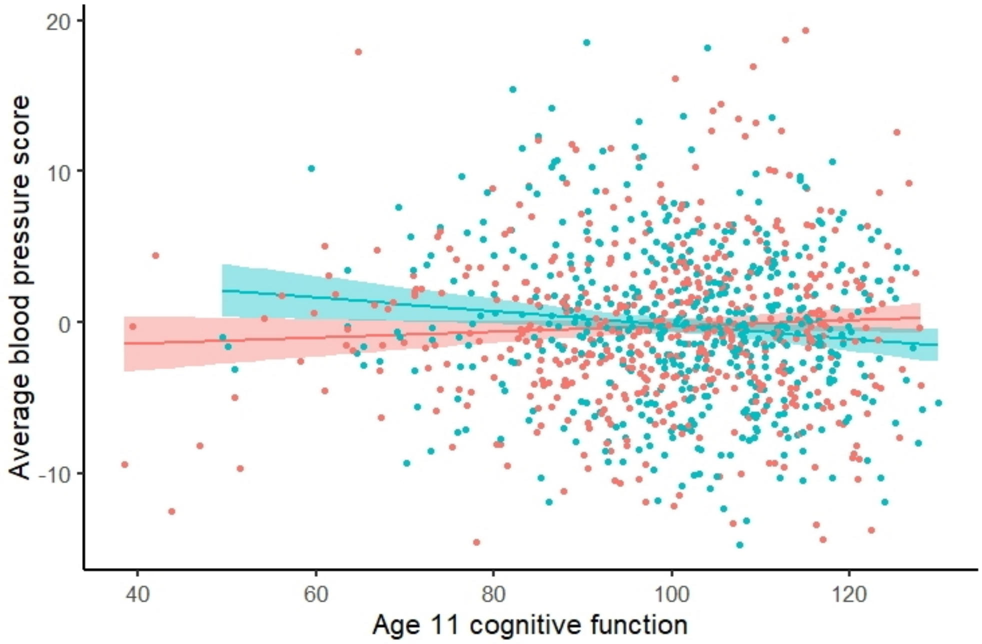

Figure 3. Blood pressure in older age and its relationship with cognitive function at age 11 years, sex, and their interaction. Blood pressure score was derived from the latent variable that represent blood pressure in individual waves. The green dots and line represent women, and the red dots and line represent men. The shaded areas represent 95% confidence regions.

63x41mm (300 x 300 DPI)

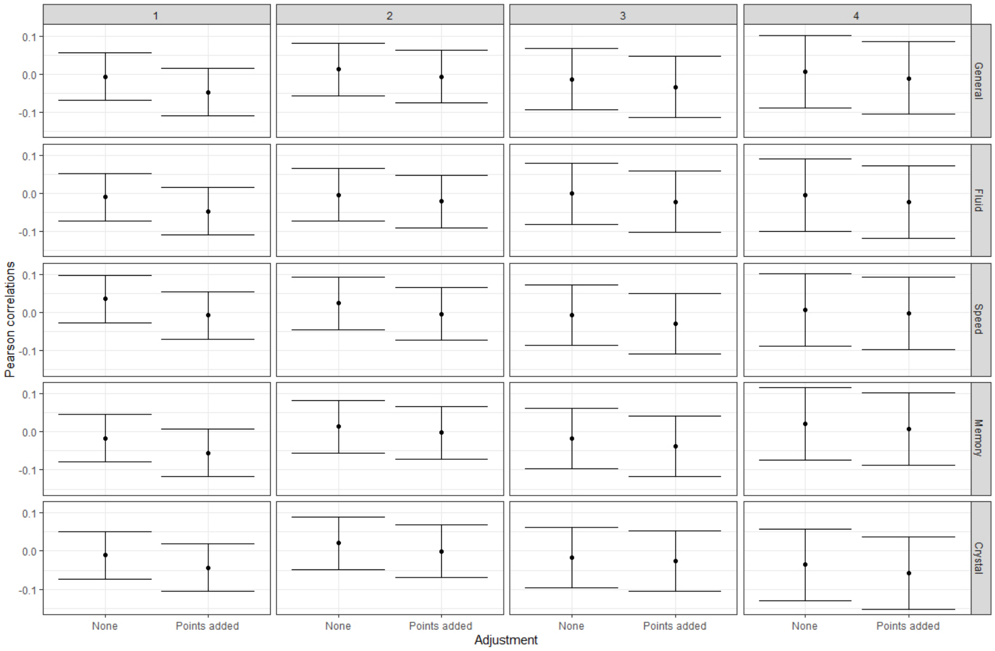

Figure 4. Pearson correlations between general and subdomain cognitive function scores and blood pressure scores within each wave of the Lothian Birth Cohort. Wave is indicated across the top of each column; the left side of each pane represents correlations among data without adjustment for anti-hypertensive treatment, and the right side of each pane represents correlations among data with adjustment for anti-hypertensive treatment. Error bars are 95% confidence intervals around the correlation coefficient.

Blood pressure and cognitive function across the eighth decade: a prospective study of the Lothian Birth Cohort of 1936

Supplementary Materials

Supplementary Figures 1, 2

Supplementary Tables 1 – 4

For peer review only

Supplementary Figure 1. Simplified path diagram of the hierarchical curve of factors latent growth model of blood pressure change in the LBC1936. Circles represent latent variables and squares represent measured variables. Growth curves, including latent level, linear slope, and quadrative slope factors, were estimated from both systolic and diastolic reading. Basis coefficients (loadings on the individual test slopes) were fixed at 0, 2.96, 6.72, and 9.79 for the linear slops and 0, 8.76, 45.16, and 95.84 for the quadratic slopes to precisely reflect the amount of time passing between assessments. Dashed lines indicate the relationships between covariates which differed between waves and their effects on the individual measurements at different waves. To preserve interpretability, not all variables and paths are shown. Solid, double arrowed lines indicate correlations between latent variables. Dashed lines ending in diamonds represent systolic and diastolic measurements, modelled the same way as at age 70.

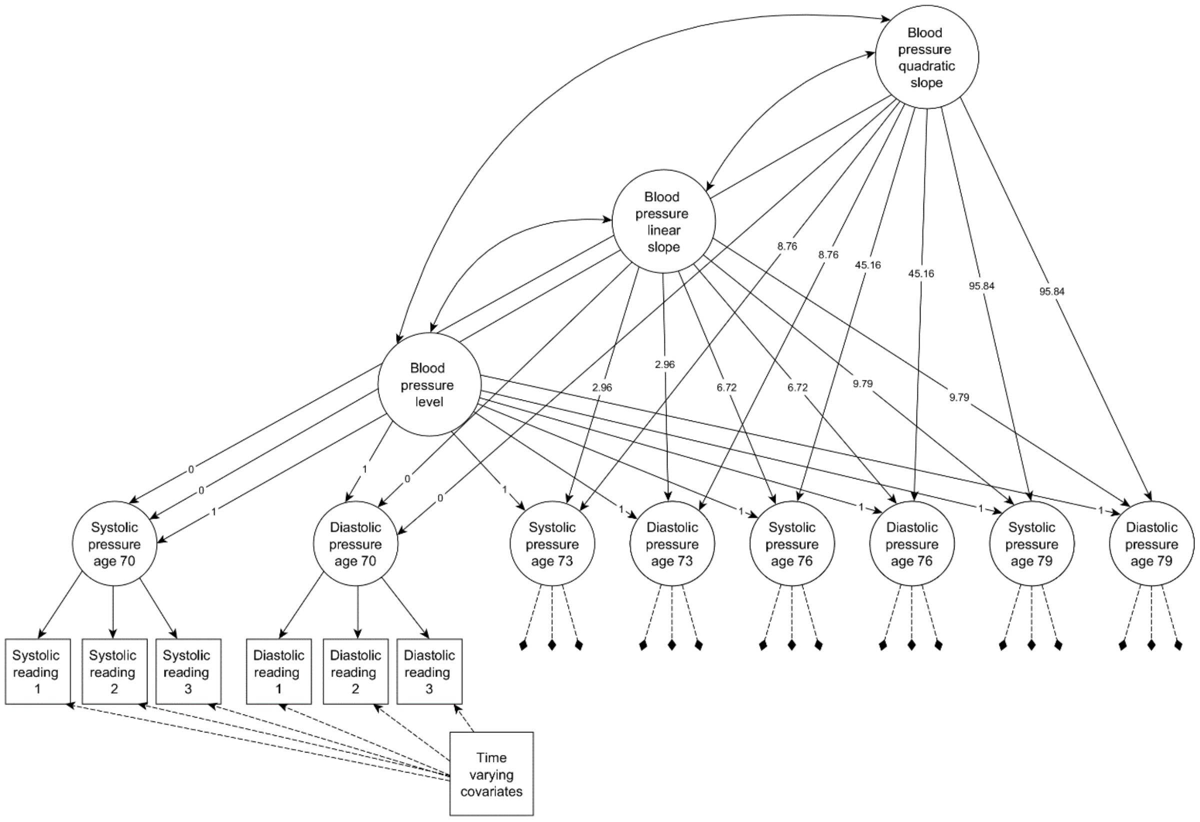

Supplementary Figure 2. Simplified path diagram of the bivariate growth curves of blood pressure and cognitive function, as well as the underlying structure of cognitive function. Circles represent latent variables and squares represent measured variables. Latent variables for blood pressure were modelled as shown in Figure 1. Growth curves, including a latent level and linear slope factor, were estimated for each cognitive test, and the intercepts and slopes were analyzed in a hierarchical model which contained domain and overall factors of both level and slope. Basis coefficients (loadings on the individual test slopes) were fixed at 0, 2.96, 6.72, and 9.79 to precisely represent the amount of time passing between assessments. Dashed lines indicate the relationships between covariates which were fixed across waves and the overall and domain latent variables. To preserve interpretability, not all covariate relationships are shown. Solid, double arrowed lines indicate correlations between latent variables. Although only two domains and four tests are shown here, the full model used all fourteen tests and four domains, as described in the Methods.

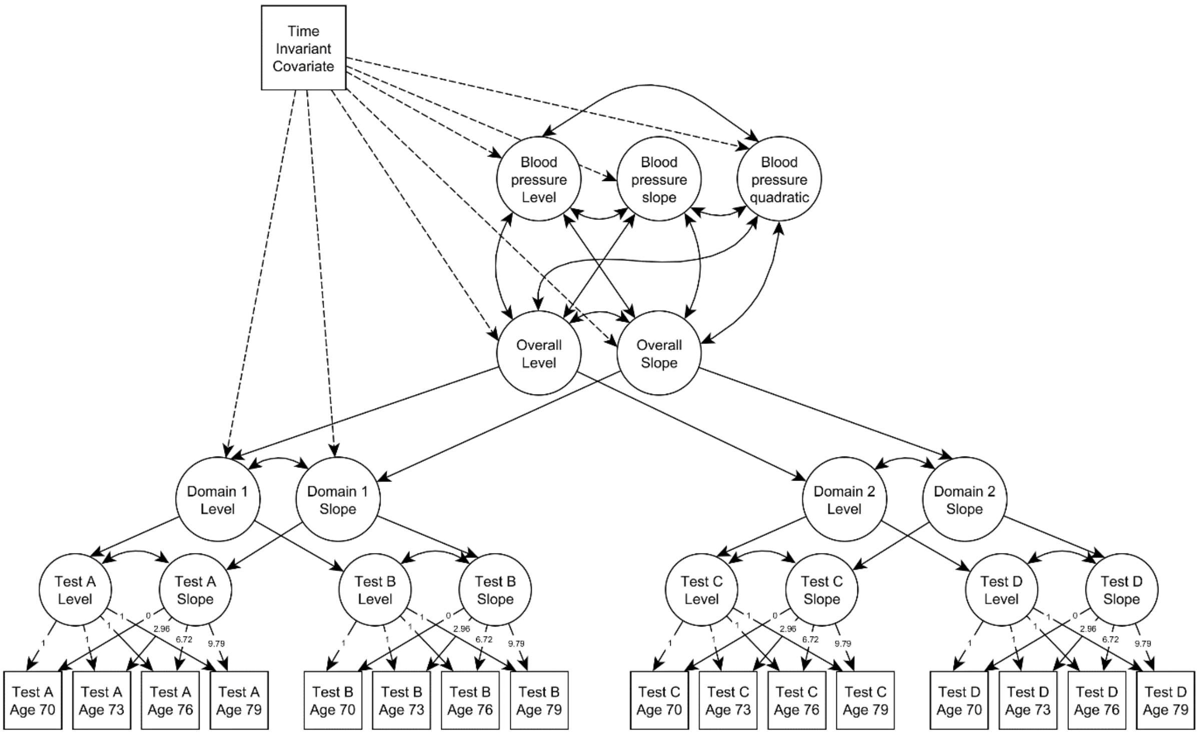

For peer review only - http://bmjopen.bmj.com/site/about/guidelines.xhtml

Supplementary Table 1. Descriptive statistics for cognitive, demographic, and clinical variables from study completers only (n = 539).

	Wave 1	Wave 2	Wave 3	Wave 4
Female	267 (49.5%)	-	-	-
Ever had high BP	193 (35.8%)	256 (47.5%)	286 (53.1%)	314 (58.3%)
Ever had CVD	123 (22.8%)	151 (28.0%)	185 (34.3%)	198 (36.7%)
Ever had stroke	18 (3.33%)	31 (5.75%)	55 (10.2%)	69 (12.5%)
Current smoker	24 (4.54%)	22 (4.08%)	19 (3.52%)	21 (3.90%)
Ex-smoker	226 (41.9%)	228 (42.3%)	222 (41.2%)	229 (42.4%)
Average sitting systolic BP	147.7 (17.63)	147.7 (18.18)	147.7 (18.75)	145.1 (18.7)
Average sitting diastolic BP	81.0 (9.53)	77.9 (9.53)	79.0 (10.3)	76.8 (10.1)
Years of education	10.9 (1.17)	-	-	-
Age 11 IQ	102.2 (15.15)	-	-	-
Matrix Reasoning*	14.7 (5.02)	13.2 (4.89)	13.3 (4.91)	13.0 (5.02)
Block Design*	35.7 (10.1)	34.7 (10.1)	32.7 (9.79)	31.3 (9.66)
Spatial Span*	7.27 (1.71)	7.15 (1.58)	7.15 (1.58)	6.75 (1.60)
NART†	35.6 (7.97)	35.2 (7.99)	35.6 (7.95)	35.7 (8.10)
WTAR†	41.9 (6.95)	41.8 (6.61)	41.6 (6.93)	41.7 (6.97)
Verbal Fluency†	43.6 (12.8)	44.4 (13.0)	43.7 (12.7)	43.7 (13.3)
Logical Memory‡	74.4 (16.9)	76.3 (16.9)	75.8 (18.4)	73.0 (20.3)
VPA‡	27.9 (8.53)	28.4 (9.04)	27.3 (9.25)	27.3 (9.44)
Digit Span‡	8.03 (2.37)	8.01 (2.32)	7.91 (2.41)	7.58 (2.19)
LNS‡	11.4 (3.11)	11.4 (3.08)	10.7 (2.98)	10.1 (2.87)
Symbol Search§	25.6 (6.58)	25.5 (5.92)	25.3 (6.26)	22.8 (6.64)
Digit-Symbol Coding§	59.0 (12.4)	58.4 (12.0)	55.4 (12.3)	51.4 (13.0)
Inspection Time§	113 (10.5)	112 (11.7)	111 (11.8)	107 (13.6)
Reaction Time§	0.63 (0.08)	0.64 (0.08)	0.67 (0.09)	0.70 (0.11)
MMSE	28.9 (1.28)	28.9 (1.21)	28.8 (1.53)	28.5 (2.16)

CVD, cardiovascular disease; BP, blood pressure; NART, National Adult Reading Test; WTAR, Wechsler Test of Adult Reading; VPA, Visual Paired Associates; LNS, Letter-Number Sequencing; MMSE, Mini-Mental State Examination; T2D, individuals with type 2 diabetes; T2D was defined as self-reported physician diagnosis of diabetes. * part of the visuospatial ability domain, † part of the crystallized ability domain, ‡ part of the memory domain, § part of the processing speed domain.

For peer review only

Supplementary Table 2. Bivariate associations of the latent growth curve model (1A) of blood pressure and cognitive function.

LHS	Path	RHS	Std. Estimate	S.E.
Blood pressure level	~~	Blood pressure quadratic	0.293	0.065
Blood pressure slope	~~	Blood pressure quadratic	-0.926	0.017
Blood pressure level	~~	Blood pressure slope	-0.424	0.052
Cognitive function level	~~	Cognitive function slope	0.326	0.059
Fluid function level	~~	Fluid function slope	-0.570	1.150
Processing speed level	~~	Processing speed slope	-0.255	0.123
Memory ability level	~~	Memory ability slope	-0.394	0.108
Crystallized ability level	~~	Crystallized ability slope	-0.277	0.377
Blood pressure level	~~	Cognitive function level	-0.024	0.036
Blood pressure slope	~~	Cognitive function slope	-0.097	0.077
Blood pressure quadratic	~~	Cognitive function slope	0.171	0.076
Blood pressure level	~~	Cognitive function slope	0.046	0.043
Blood pressure slope	~~	Cognitive function level	-0.003	0.055
Blood pressure quadratic	~~	Cognitive function level	0.015	0.069
Cognitive function slope	~	Sex	0.110	0.043
Cognitive function slope	~	Age 11 cognitive function	0.016	0.044
Cognitive function slope	~	Education	-0.069	0.049
Cognitive function slope	~	Sex x age 11 CF	-0.078	0.054
Blood pressure quadratic	~	Sex	0.102	0.054
Blood pressure quadratic	~	Age 11 cognitive function	0.023	0.095
Blood pressure quadratic	~	Education	0.060	0.060
Blood pressure quadratic	~	Sex x age 11 CF	-0.122	0.085
Blood pressure slope	~	Sex	-0.020	0.048
Blood pressure slope	~	Age 11 cognitive function	-0.031	0.079
Blood pressure slope	~	Education	-0.058	0.059
Blood pressure slope	~	Sex x age 11 CF	0.116	0.074

For peer review only

Blood pressure level	~	Sex	-0.059	0.032
Blood pressure level	~	Age 11 cognitive function	0.079	0.049
Blood pressure level	~	Education	-0.024	0.038
Blood pressure level	~	Sex x age 11 CF	-0.136	0.050
Cognitive function level	~	Sex x age 11 CF	0.022	0.029
Cognitive function level	~	Sex	-0.050	0.026
Cognitive function level	~	Age 11 cognitive function	0.694	0.037
Cognitive function level	~	Education	0.222	0.019
Systolic pressure measurement 1, wave 1	~	Age at wave 1	-0.010	0.025
Systolic pressure measurement 2, wave 1	~	Age at wave 1	-0.040	0.024
Systolic pressure measurement 3, wave 1	~	Age at wave 1	-0.057	0.028
Diastolic pressure measurement 1, wave 1	~	Age at wave 1	-0.074	0.025
Diastolic pressure measurement 2, wave 1	~	Age at wave 1	-0.061	0.027
Diastolic pressure measurement 3, wave 1	~	Age at wave 1	-0.089	0.028
Systolic pressure measurement 1, wave 2	~	Age at wave 2	-0.029	0.030
Systolic pressure measurement 2, wave 2	~	Age at wave 2	0.001	0.029
Systolic pressure measurement 3, wave 2	~	Age at wave 2	0.010	0.033
Diastolic pressure measurement 1, wave 2	~	Age at wave 2	0.019	0.039
Diastolic pressure measurement 2, wave 2	~	Age at wave 2	0.020	0.035
Diastolic pressure measurement 3, wave 2	~	Age at wave 2	0.014	0.033
Systolic pressure measurement 1, wave 3	~	Age at wave 3	-0.069	0.036
Systolic pressure measurement 2, wave 3	~	Age at wave 3	-0.092	0.032
Systolic pressure measurement 3, wave 3	~	Age at wave 3	-0.081	0.034
Diastolic pressure measurement 1, wave 3	~	Age at wave 3	0.026	0.038
Diastolic pressure measurement 2, wave 3	~	Age at wave 3	0.028	0.036
Diastolic pressure measurement 3, wave 3	~	Age at wave 3	0.014	0.039
Systolic pressure measurement 1, wave 4	~	Age at wave 4	-0.043	0.032
Systolic pressure measurement 2, wave 4	~	Age at wave 4	-0.077	0.027
Systolic pressure measurement 3, wave 4	~	Age at wave 4	-0.051	0.028
Diastolic pressure measurement 1, wave 4	~	Age at wave 4	0.049	0.042
Diastolic pressure measurement 2, wave 4	~	Age at wave 4	0.039	0.030

For peer review only

Diastolic pressure measurement 3, wave 4	~	Age at wave 4	0.017	0.026
Systolic pressure measurement 1, wave 1	~~	Systolic pressure measurement 1, wave 1	0.131	0.010
Systolic pressure measurement 2, wave 1	~~	Systolic pressure measurement 2, wave 1	0.074	0.008
Systolic pressure measurement 3, wave 1	~~	Systolic pressure measurement 3, wave 1	0.097	0.008
Diastolic pressure measurement 1, wave 1	~~	Diastolic pressure measurement 1, wave 1	0.141	0.014
Diastolic pressure measurement 2, wave 1	~~	Diastolic pressure measurement 2, wave 1	0.093	0.014
Diastolic pressure measurement 3, wave 1	~~	Diastolic pressure measurement 3, wave 1	0.150	0.015
Systolic pressure measurement 1, wave 2	~~	Systolic pressure measurement 1, wave 2	0.247	0.018
Systolic pressure measurement 2, wave 2	~~	Systolic pressure measurement 2, wave 2	0.039	0.008
Systolic pressure measurement 3, wave 2	~~	Systolic pressure measurement 3, wave 2	0.069	0.007
Diastolic pressure measurement 1, wave 2	~~	Diastolic pressure measurement 1, wave 2	0.113	0.013
Diastolic pressure measurement 2, wave 2	~~	Diastolic pressure measurement 2, wave 2	0.082	0.011
Diastolic pressure measurement 3, wave 2	~~	Diastolic pressure measurement 3, wave 2	0.094	0.016
Systolic pressure measurement 1, wave 3	~~	Systolic pressure measurement 1, wave 3	0.155	0.015
Systolic pressure measurement 2, wave 3	~~	Systolic pressure measurement 2, wave 3	0.068	0.013
Systolic pressure measurement 3, wave 3	~~	Systolic pressure measurement 3, wave 3	0.085	0.013
Diastolic pressure measurement 1, wave 3	~~	Diastolic pressure measurement 1, wave 3	0.144	0.021
Diastolic pressure measurement 2, wave 3	~~	Diastolic pressure measurement 2, wave 3	0.137	0.029
Diastolic pressure measurement 3, wave 3	~~	Diastolic pressure measurement 3, wave 3	0.168	0.016
Systolic pressure measurement 1, wave 4	~~	Systolic pressure measurement 1, wave 4	0.113	0.014
Systolic pressure measurement 2, wave 4	~~	Systolic pressure measurement 2, wave 4	0.045	0.009
Systolic pressure measurement 3, wave 4	~~	Systolic pressure measurement 3, wave 4	0.093	0.012
Diastolic pressure measurement 1, wave 4	~~	Diastolic pressure measurement 1, wave 4	0.140	0.025
Diastolic pressure measurement 2, wave 4	~~	Diastolic pressure measurement 2, wave 4	0.093	0.015
Diastolic pressure measurement 3, wave 4	~~	Diastolic pressure measurement 3, wave 4	0.102	0.011
Blood pressure level	~~	Blood pressure level	0.985	0.008
Blood pressure slope	~~	Blood pressure slope	0.989	0.013
Blood pressure quadratic	~~	Blood pressure quadratic	0.979	0.019
Fluid function level	~~	Fluid function level	0.389	0.045
Processing speed level	~~	Processing speed level	0.449	0.044
Memory ability level	~~	Memory ability level	0.258	0.034

For peer review only

Crystallized ability level	~~	Crystallized ability level	0.343	0.023
Cognitive function level	~~	Cognitive function level	0.314	0.028
Fluid function slope	~~	Fluid function slope	0.053	0.241
Processing speed slope	~~	Processing speed slope	0.177	0.089
Memory ability slope	~~	Memory ability slope	0.369	0.065
Crystallized ability slope	~~	Crystallized ability slope	0.311	0.655
Cognitive function slope	~~	Cognitive function slope	0.977	0.012
Sex	~~	Sex	1.000	0.000
Sex	~~	Age 11 cognitive function	0.065	0.000
Sex	~~	Education	-0.008	0.000
Sex	~~	Sex x age 11 CF	0.051	0.000
Sex	~~	Age at wave 1	0.004	0.000
Sex	~~	Age at wave 2	0.022	0.000
Sex	~~	Age at wave 3	-0.009	0.000
Sex	~~	Age at wave 4	0.001	0.000
Age 11 cognitive function	~~	Age 11 cognitive function	1.000	0.000
Age 11 cognitive function	~~	Education	0.432	0.000
Age 11 cognitive function	~~	Sex x age 11 CF	0.660	0.000
Age 11 cognitive function	~~	Age at wave 1	-0.097	0.000
Age 11 cognitive function	~~	Age at wave 2	-0.084	0.000
Age 11 cognitive function	~~	Age at wave 3	-0.103	0.000
Age 11 cognitive function	~~	Age at wave 4	-0.095	0.000
Education	~~	Education	1.000	0.000
Education	~~	Sex x age 11 CF	0.296	0.000
Education	~~	Age at wave 1	-0.075	0.000
Education	~~	Age at wave 2	-0.047	0.000
Education	~~	Age at wave 3	-0.085	0.000
Education	~~	Age at wave 4	-0.066	0.000
Sex x age 11 CF	~~	Sex x age 11 CF	1.000	0.000
Sex x age 11 CF	~~	Age at wave 1	-0.073	0.000
Sex x age 11 CF	~~	Age at wave 2	-0.065	0.000

For peer review only

Sex x age 11 CF	~~	Age at wave 3	-0.098	0.000
Sex x age 11 CF	~~	Age at wave 4	-0.086	0.000
Age at wave 1	~~	Age at wave 1	1.000	0.000
Age at wave 1	~~	Age at wave 2	0.945	0.000
Age at wave 1	~~	Age at wave 3	0.936	0.000
Age at wave 1	~~	Age at wave 4	0.893	0.000
Age at wave 2	~~	Age at wave 2	1.000	0.000
Age at wave 2	~~	Age at wave 3	0.922	0.000
Age at wave 2	~~	Age at wave 4	0.880	0.000
Age at wave 3	~~	Age at wave 3	1.000	0.000
Age at wave 3	~~	Age at wave 4	0.912	0.000
Age at wave 4	~~	Age at wave 4	1.000	0.000

LHS = Left-hand side (of the structural equation); RHS = right-hand side. The path column indicates the type of relationship between the two variables, ~ indicates a regression, and ~~ indicates a correlation or covariance.

For peer review only

Supplementary Table 3. Bivariate associations of the latent growth curve model (1B) of blood pressure and cognitive function with control covariates.

LHS	Path	RHS	Std. Estimate	S.E.
Blood pressure level	~~	Blood pressure quadratic	0.264	0.095
Blood pressure slope	~~	Blood pressure quadratic	-0.929	0.018
Blood pressure level	~~	Blood pressure slope	-0.385	0.077
Cognitive function level	~~	Cognitive function slope	0.358	0.095
Fluid function level	~~	Fluid function slope	-0.682	0.504
Processing speed level	~~	Processing speed slope	-0.395	0.123
Memory ability level	~~	Memory ability slope	-0.455	0.092
Crystallized ability level	~~	Crystallized ability slope	-0.174	0.330
Blood pressure level	~~	Cognitive function level	-0.046	0.059
Blood pressure slope	~~	Cognitive function slope	-0.110	0.081
Blood pressure quadratic	~~	Cognitive function slope	0.168	0.092
Blood pressure level	~~	Cognitive function slope	0.063	0.057
Blood pressure slope	~~	Cognitive function level	0.038	0.090
Blood pressure quadratic	~~	Cognitive function level	-0.005	0.092
Cognitive function slope	~	Age 11 cognitive function	-0.011	0.088
Cognitive function slope	~	Education	-0.081	0.064
Cognitive function slope	~	APOEe4	-0.183	0.064
Cognitive function slope	~	Sex	0.111	0.055
Cognitive function slope	~	Sex x age 11 CF	0.027	0.066
Blood pressure quadratic	~	Age 11 cognitive function	-0.043	0.112
Blood pressure quadratic	~	Education	0.156	0.088
Blood pressure quadratic	~	Current smoker	-0.194	0.100
Blood pressure quadratic	~	Former Current smoker	-0.012	0.075

For peer review only

Blood pressure quadratic	~	Sex	0.171	0.061
Blood pressure quadratic	~	Sex x age 11 CF	-0.035	0.110
Blood pressure slope	~	Age 11 cognitive function	0.002	0.099
Blood pressure slope	~	Education	-0.140	0.075
Blood pressure slope	~	Current smoker	0.178	0.089
Blood pressure slope	~	Former Current smoker	0.014	0.072
Blood pressure slope	~	Sex	-0.073	0.049
Blood pressure slope	~	Sex x age 11 CF	0.054	0.102
Blood pressure level	~	Age 11 cognitive function	0.099	0.072
Blood pressure level	~	Education	-0.029	0.053
Blood pressure level	~	Current smoker	-0.104	0.040
Blood pressure level	~	Former Current smoker	0.011	0.047
Blood pressure level	~	Sex	-0.055	0.046
Blood pressure level	~	Sex x age 11 CF	-0.106	0.058
Cognitive function level	~	Age 11 cognitive function	0.741	0.035
Cognitive function level	~	Education	0.242	0.031
Cognitive function level	~	APOEe4	-0.083	0.027
Cognitive function level	~	Sex	-0.051	0.025
Cognitive function level	~	Sex x age 11 CF	0.011	0.051
Systolic pressure measurement 1, wave 1	~	Age at wave 1	-0.050	0.047
Systolic pressure measurement 1, wave 1	~	CVD history at wave 1	0.011	0.038
Systolic pressure measurement 1, wave 1	~	Stroke history at wave 1	0.037	0.047
Systolic pressure measurement 2, wave 1	~	Age at wave 1	-0.097	0.040
Systolic pressure measurement 2, wave 1	~	CVD history at wave 1	0.005	0.039
Systolic pressure measurement 2, wave 1	~	Stroke history at wave 1	0.063	0.049
Systolic pressure measurement 3, wave 1	~	Age at wave 1	-0.096	0.048
Systolic pressure measurement 3, wave 1	~	CVD history at wave 1	0.012	0.037
Systolic pressure measurement 3, wave 1	~	Stroke history at wave 1	0.061	0.048
Diastolic pressure measurement 1, wave 1	~	Age at wave 1	-0.103	0.038
Diastolic pressure measurement 1, wave 1	~	CVD history at wave 1	-0.050	0.045

For peer review only

Diastolic pressure measurement 1, wave 1	~	Stroke history at wave 1	0.028	0.029
Diastolic pressure measurement 2, wave 1	~	Age at wave 1	-0.079	0.044
Diastolic pressure measurement 2, wave 1	~	CVD history at wave 1	-0.012	0.045
Diastolic pressure measurement 2, wave 1	~	Stroke history at wave 1	0.030	0.028
Diastolic pressure measurement 3, wave 1	~	Age at wave 1	-0.102	0.037
Diastolic pressure measurement 3, wave 1	~	CVD history at wave 1	-0.041	0.044
Diastolic pressure measurement 3, wave 1	~	Stroke history at wave 1	0.035	0.027
Systolic pressure measurement 1, wave 2	~	Age at wave 2	-0.024	0.043
Systolic pressure measurement 1, wave 2	~	CVD history at wave 2	-0.122	0.037
Systolic pressure measurement 1, wave 2	~	Stroke history at wave 2	0.025	0.042
Systolic pressure measurement 2, wave 2	~	Age at wave 2	0.011	0.049
Systolic pressure measurement 2, wave 2	~	CVD history at wave 2	-0.045	0.035
Systolic pressure measurement 2, wave 2	~	Stroke history at wave 2	-0.014	0.045
Systolic pressure measurement 3, wave 2	~	Age at wave 2	0.026	0.046
Systolic pressure measurement 3, wave 2	~	CVD history at wave 2	-0.026	0.031
Systolic pressure measurement 3, wave 2	~	Stroke history at wave 2	-0.011	0.042
Diastolic pressure measurement 1, wave 2	~	Age at wave 2	0.048	0.043
Diastolic pressure measurement 1, wave 2	~	CVD history at wave 2	-0.053	0.053
Diastolic pressure measurement 1, wave 2	~	Stroke history at wave 2	-0.017	0.037
Diastolic pressure measurement 2, wave 2	~	Age at wave 2	0.053	0.041
Diastolic pressure measurement 2, wave 2	~	CVD history at wave 2	-0.051	0.051
Diastolic pressure measurement 2, wave 2	~	Stroke history at wave 2	-0.019	0.035
Diastolic pressure measurement 3, wave 2	~	Age at wave 2	0.042	0.042
Diastolic pressure measurement 3, wave 2	~	CVD history at wave 2	-0.037	0.053
Diastolic pressure measurement 3, wave 2	~	Stroke history at wave 2	-0.014	0.038
Systolic pressure measurement 1, wave 3	~	Age at wave 3	-0.101	0.048
Systolic pressure measurement 1, wave 3	~	CVD history at wave 3	-0.108	0.037
Systolic pressure measurement 1, wave 3	~	Stroke history at wave 3	-0.078	0.040
Systolic pressure measurement 2, wave 3	~	Age at wave 3	-0.092	0.048
Systolic pressure measurement 2, wave 3	~	CVD history at wave 3	-0.116	0.035

For peer review only

Systolic pressure measurement 2, wave 3	~	Stroke history at wave 3	-0.045	0.041
Systolic pressure measurement 3, wave 3	~	Age at wave 3	-0.103	0.049
Systolic pressure measurement 3, wave 3	~	CVD history at wave 3	-0.083	0.038
Systolic pressure measurement 3, wave 3	~	Stroke history at wave 3	-0.028	0.041
Diastolic pressure measurement 1, wave 3	~	Age at wave 3	0.043	0.041
Diastolic pressure measurement 1, wave 3	~	CVD history at wave 3	-0.055	0.056
Diastolic pressure measurement 1, wave 3	~	Stroke history at wave 3	-0.047	0.046
Diastolic pressure measurement 2, wave 3	~	Age at wave 3	0.060	0.043
Diastolic pressure measurement 2, wave 3	~	CVD history at wave 3	-0.033	0.042
Diastolic pressure measurement 2, wave 3	~	Stroke history at wave 3	-0.044	0.045
Diastolic pressure measurement 3, wave 3	~	Age at wave 3	0.026	0.044
Diastolic pressure measurement 3, wave 3	~	CVD history at wave 3	-0.049	0.053
Diastolic pressure measurement 3, wave 3	~	Stroke history at wave 3	-0.046	0.045
Systolic pressure measurement 1, wave 4	~	Age at wave 4	-0.062	0.031
Systolic pressure measurement 1, wave 4	~	CVD history at wave 4	0.013	0.034
Systolic pressure measurement 1, wave 4	~	Stroke history at wave 4	-0.082	0.037
Systolic pressure measurement 2, wave 4	~	Age at wave 4	-0.092	0.031
Systolic pressure measurement 2, wave 4	~	CVD history at wave 4	-0.008	0.034
Systolic pressure measurement 2, wave 4	~	Stroke history at wave 4	-0.029	0.040
Systolic pressure measurement 3, wave 4	~	Age at wave 4	-0.068	0.036
Systolic pressure measurement 3, wave 4	~	CVD history at wave 4	0.004	0.037
Systolic pressure measurement 3, wave 4	~	Stroke history at wave 4	-0.058	0.044
Diastolic pressure measurement 1, wave 4	~	Age at wave 4	0.056	0.046
Diastolic pressure measurement 1, wave 4	~	CVD history at wave 4	-0.061	0.038
Diastolic pressure measurement 1, wave 4	~	Stroke history at wave 4	-0.058	0.045
Diastolic pressure measurement 2, wave 4	~	Age at wave 4	0.039	0.043
Diastolic pressure measurement 2, wave 4	~	CVD history at wave 4	-0.049	0.042
Diastolic pressure measurement 2, wave 4	~	Stroke history at wave 4	-0.030	0.046
Diastolic pressure measurement 3, wave 4	~	Age at wave 4	0.024	0.045
Diastolic pressure measurement 3, wave 4	~	CVD history at wave 4	-0.042	0.044

For peer review only

Diastolic pressure measurement 3, wave 4	~	Stroke history at wave 4	-0.022	0.049
Systolic pressure measurement 1, wave 1	~~	Systolic pressure measurement 1, wave 1	0.129	0.015
Systolic pressure measurement 2, wave 1	~~	Systolic pressure measurement 2, wave 1	0.093	0.010
Systolic pressure measurement 3, wave 1	~~	Systolic pressure measurement 3, wave 1	0.097	0.013
Diastolic pressure measurement 1, wave 1	~~	Diastolic pressure measurement 1, wave 1	0.135	0.018
Diastolic pressure measurement 2, wave 1	~~	Diastolic pressure measurement 2, wave 1	0.099	0.013
Diastolic pressure measurement 3, wave 1	~~	Diastolic pressure measurement 3, wave 1	0.165	0.019
Systolic pressure measurement 1, wave 2	~~	Systolic pressure measurement 1, wave 2	0.197	0.016
Systolic pressure measurement 2, wave 2	~~	Systolic pressure measurement 2, wave 2	0.039	0.008
Systolic pressure measurement 3, wave 2	~~	Systolic pressure measurement 3, wave 2	0.082	0.009
Diastolic pressure measurement 1, wave 2	~~	Diastolic pressure measurement 1, wave 2	0.100	0.015
Diastolic pressure measurement 2, wave 2	~~	Diastolic pressure measurement 2, wave 2	0.075	0.011
Diastolic pressure measurement 3, wave 2	~~	Diastolic pressure measurement 3, wave 2	0.110	0.021
Systolic pressure measurement 1, wave 3	~~	Systolic pressure measurement 1, wave 3	0.141	0.018
Systolic pressure measurement 2, wave 3	~~	Systolic pressure measurement 2, wave 3	0.083	0.010
Systolic pressure measurement 3, wave 3	~~	Systolic pressure measurement 3, wave 3	0.085	0.012
Diastolic pressure measurement 1, wave 3	~~	Diastolic pressure measurement 1, wave 3	0.135	0.022
Diastolic pressure measurement 2, wave 3	~~	Diastolic pressure measurement 2, wave 3	0.146	0.034
Diastolic pressure measurement 3, wave 3	~~	Diastolic pressure measurement 3, wave 3	0.172	0.025
Systolic pressure measurement 1, wave 4	~~	Systolic pressure measurement 1, wave 4	0.118	0.013
Systolic pressure measurement 2, wave 4	~~	Systolic pressure measurement 2, wave 4	0.042	0.007
Systolic pressure measurement 3, wave 4	~~	Systolic pressure measurement 3, wave 4	0.104	0.013
Diastolic pressure measurement 1, wave 4	~~	Diastolic pressure measurement 1, wave 4	0.148	0.023
Diastolic pressure measurement 2, wave 4	~~	Diastolic pressure measurement 2, wave 4	0.102	0.018
Diastolic pressure measurement 3, wave 4	~~	Diastolic pressure measurement 3, wave 4	0.098	0.015
Blood pressure level	~~	Blood pressure level	0.976	0.015
Blood pressure slope	~~	Blood pressure slope	0.950	0.035
Blood pressure quadratic	~~	Blood pressure quadratic	0.922	0.045
Fluid function level	~~	Fluid function level	0.489	0.052
Processing speed level	~~	Processing speed level	0.545	0.051

For peer review only

Memory ability level	~~	Memory ability level	0.350	0.045
Crystallized ability level	~~	Crystallized ability level	0.325	0.037
Cognitive function level	~~	Cognitive function level	0.212	0.031
Fluid function slope	~~	Fluid function slope	0.158	0.249
Processing speed slope	~~	Processing speed slope	0.239	0.100
Memory ability slope	~~	Memory ability slope	0.409	0.104
Crystallized ability slope	~~	Crystallized ability slope	0.361	0.916
Cognitive function slope	~~	Cognitive function slope	0.944	0.026
Age 11 cognitive function	~~	Age 11 cognitive function	1.000	0.000
Age 11 cognitive function	~~	Education	0.445	0.000
Age 11 cognitive function	~~	APOEe4	-0.043	0.000
Age 11 cognitive function	~~	Sex	0.091	0.000
Age 11 cognitive function	~~	Sex x age 11 CF	0.659	0.000
Age 11 cognitive function	~~	Current smoker	0.012	0.000
Age 11 cognitive function	~~	Former Current smoker	-0.092	0.000
Age 11 cognitive function	~~	Age at wave 1	-0.090	0.000
Age 11 cognitive function	~~	CVD history at wave 1	-0.128	0.000
Age 11 cognitive function	~~	Stroke history at wave 1	0.001	0.000
Age 11 cognitive function	~~	Age at wave 2	-0.076	0.000
Age 11 cognitive function	~~	CVD history at wave 2	-0.097	0.000
Age 11 cognitive function	~~	Stroke history at wave 2	0.039	0.000
Age 11 cognitive function	~~	Age at wave 3	-0.081	0.000
Age 11 cognitive function	~~	CVD history at wave 3	-0.055	0.000
Age 11 cognitive function	~~	Stroke history at wave 3	0.019	0.000
Age 11 cognitive function	~~	Age at wave 4	-0.073	0.000
Age 11 cognitive function	~~	CVD history at wave 4	-0.043	0.000
Age 11 cognitive function	~~	Stroke history at wave 4	-0.027	0.000
Education	~~	Education	1.000	0.000
Education	~~	APOEe4	-0.028	0.000
Education	~~	Sex	0.025	0.000

For peer review only

Education	~~	Sex x age 11 CF	0.263	0.000
Education	~~	Current smoker	0.046	0.000
Education	~~	Former Current smoker	-0.091	0.000
Education	~~	Age at wave 1	-0.088	0.000
Education	~~	CVD history at wave 1	-0.056	0.000
Education	~~	Stroke history at wave 1	-0.035	0.000
Education	~~	Age at wave 2	-0.054	0.000
Education	~~	CVD history at wave 2	-0.043	0.000
Education	~~	Stroke history at wave 2	-0.006	0.000
Education	~~	Age at wave 3	-0.098	0.000
Education	~~	CVD history at wave 3	-0.042	0.000
Education	~~	Stroke history at wave 3	0.032	0.000
Education	~~	Age at wave 4	-0.075	0.000
Education	~~	CVD history at wave 4	-0.064	0.000
Education	~~	Stroke history at wave 4	0.033	0.000
APOEe4	~~	APOEe4	1.000	0.000
APOEe4	~~	Sex	-0.104	0.000
APOEe4	~~	Sex x age 11 CF	-0.013	0.000
APOEe4	~~	Current smoker	-0.111	0.000
APOEe4	~~	Former Current smoker	0.009	0.000
APOEe4	~~	Age at wave 1	0.048	0.000
APOEe4	~~	CVD history at wave 1	0.013	0.000
APOEe4	~~	Stroke history at wave 1	-0.047	0.000
APOEe4	~~	Age at wave 2	0.030	0.000
APOEe4	~~	CVD history at wave 2	0.017	0.000
APOEe4	~~	Stroke history at wave 2	-0.044	0.000
APOEe4	~~	Age at wave 3	0.055	0.000
APOEe4	~~	CVD history at wave 3	0.015	0.000
APOEe4	~~	Stroke history at wave 3	0.000	0.000
APOEe4	~~	Age at wave 4	0.033	0.000

For peer review only

APOEe4	~~	CVD history at wave 4	-0.001	0.000
APOEe4	~~	Stroke history at wave 4	0.000	0.000
Sex	~~	Sex	1.000	0.000
Sex	~~	Sex x age 11 CF	0.179	0.000
Sex	~~	Current smoker	0.078	0.000
Sex	~~	Former Current smoker	-0.123	0.000
Sex	~~	Age at wave 1	0.020	0.000
Sex	~~	CVD history at wave 1	-0.122	0.000
Sex	~~	Stroke history at wave 1	-0.021	0.000
Sex	~~	Age at wave 2	0.043	0.000
Sex	~~	CVD history at wave 2	-0.149	0.000
Sex	~~	Stroke history at wave 2	-0.024	0.000
Sex	~~	Age at wave 3	0.003	0.000
Sex	~~	CVD history at wave 3	-0.113	0.000
Sex	~~	Stroke history at wave 3	-0.038	0.000
Sex	~~	Age at wave 4	0.018	0.000
Sex	~~	CVD history at wave 4	-0.084	0.000
Sex	~~	Stroke history at wave 4	-0.047	0.000
Sex x age 11 CF	~~	Sex x age 11 CF	1.000	0.000
Sex x age 11 CF	~~	Current smoker	0.021	0.000
Sex x age 11 CF	~~	Former Current smoker	-0.066	0.000
Sex x age 11 CF	~~	Age at wave 1	-0.046	0.000
Sex x age 11 CF	~~	CVD history at wave 1	-0.104	0.000
Sex x age 11 CF	~~	Stroke history at wave 1	-0.018	0.000
Sex x age 11 CF	~~	Age at wave 2	-0.032	0.000
Sex x age 11 CF	~~	CVD history at wave 2	-0.082	0.000
Sex x age 11 CF	~~	Stroke history at wave 2	0.027	0.000
Sex x age 11 CF	~~	Age at wave 3	-0.068	0.000
Sex x age 11 CF	~~	CVD history at wave 3	-0.054	0.000
Sex x age 11 CF	~~	Stroke history at wave 3	0.016	0.000

For peer review only

Sex x age 11 CF	~~	Age at wave 4	-0.047	0.000
Sex x age 11 CF	~~	CVD history at wave 4	-0.073	0.000
Sex x age 11 CF	~~	Stroke history at wave 4	-0.041	0.000
Current smoker	~~	Current smoker	1.000	0.000
Current smoker	~~	Former Current smoker	-0.176	0.000
Current smoker	~~	Age at wave 1	0.056	0.000
Current smoker	~~	CVD history at wave 1	0.069	0.000
Current smoker	~~	Stroke history at wave 1	0.081	0.000
Current smoker	~~	Age at wave 2	0.049	0.000
Current smoker	~~	CVD history at wave 2	0.085	0.000
Current smoker	~~	Stroke history at wave 2	0.043	0.000
Current smoker	~~	Age at wave 3	0.026	0.000
Current smoker	~~	CVD history at wave 3	0.054	0.000
Current smoker	~~	Stroke history at wave 3	0.006	0.000
Current smoker	~~	Age at wave 4	0.058	0.000
Current smoker	~~	CVD history at wave 4	0.020	0.000
Current smoker	~~	Stroke history at wave 4	0.026	0.000
Former Current smoker	~~	Former Current smoker	1.000	0.000
Former Current smoker	~~	Age at wave 1	0.006	0.000
Former Current smoker	~~	CVD history at wave 1	0.109	0.000
Former Current smoker	~~	Stroke history at wave 1	0.004	0.000
Former Current smoker	~~	Age at wave 2	-0.025	0.000
Former Current smoker	~~	CVD history at wave 2	0.102	0.000
Former Current smoker	~~	Stroke history at wave 2	0.027	0.000
Former Current smoker	~~	Age at wave 3	-0.002	0.000
Former Current smoker	~~	CVD history at wave 3	0.080	0.000
Former Current smoker	~~	Stroke history at wave 3	0.076	0.000
Former Current smoker	~~	Age at wave 4	-0.011	0.000
Former Current smoker	~~	CVD history at wave 4	0.130	0.000
Former Current smoker	~~	Stroke history at wave 4	0.053	0.000

For peer review only

Age at wave 1	~~	Age at wave 1	1.000	0.000
Age at wave 1	~~	CVD history at wave 1	0.111	0.000
Age at wave 1	~~	Stroke history at wave 1	0.148	0.000
Age at wave 1	~~	Age at wave 2	0.949	0.000
Age at wave 1	~~	CVD history at wave 2	0.080	0.000
Age at wave 1	~~	Stroke history at wave 2	0.110	0.000
Age at wave 1	~~	Age at wave 3	0.944	0.000
Age at wave 1	~~	CVD history at wave 3	0.035	0.000
Age at wave 1	~~	Stroke history at wave 3	0.067	0.000
Age at wave 1	~~	Age at wave 4	0.893	0.000
Age at wave 1	~~	CVD history at wave 4	0.098	0.000
Age at wave 1	~~	Stroke history at wave 4	0.024	0.000
CVD history at wave 1	~~	CVD history at wave 1	1.000	0.000
CVD history at wave 1	~~	Stroke history at wave 1	0.010	0.000
CVD history at wave 1	~~	Age at wave 2	0.102	0.000
CVD history at wave 1	~~	CVD history at wave 2	0.779	0.000
CVD history at wave 1	~~	Stroke history at wave 2	-0.002	0.000
CVD history at wave 1	~~	Age at wave 3	0.105	0.000
CVD history at wave 1	~~	CVD history at wave 3	0.635	0.000
CVD history at wave 1	~~	Stroke history at wave 3	0.027	0.000
CVD history at wave 1	~~	Age at wave 4	0.085	0.000
CVD history at wave 1	~~	CVD history at wave 4	0.567	0.000
CVD history at wave 1	~~	Stroke history at wave 4	0.036	0.000
Stroke history at wave 1	~~	Stroke history at wave 1	1.000	0.000
Stroke history at wave 1	~~	Age at wave 2	0.148	0.000
Stroke history at wave 1	~~	CVD history at wave 2	-0.014	0.000
Stroke history at wave 1	~~	Stroke history at wave 2	0.761	0.000
Stroke history at wave 1	~~	Age at wave 3	0.132	0.000
Stroke history at wave 1	~~	CVD history at wave 3	-0.014	0.000
Stroke history at wave 1	~~	Stroke history at wave 3	0.570	0.000

For peer review only

Stroke history at wave 1	~~	Age at wave 4	0.132	0.000
Stroke history at wave 1	~~	CVD history at wave 4	-0.047	0.000
Stroke history at wave 1	~~	Stroke history at wave 4	0.511	0.000
Age at wave 2	~~	Age at wave 2	1.000	0.000
Age at wave 2	~~	CVD history at wave 2	0.056	0.000
Age at wave 2	~~	Stroke history at wave 2	0.111	0.000
Age at wave 2	~~	Age at wave 3	0.923	0.000
Age at wave 2	~~	CVD history at wave 3	0.020	0.000
Age at wave 2	~~	Stroke history at wave 3	0.060	0.000
Age at wave 2	~~	Age at wave 4	0.877	0.000
Age at wave 2	~~	CVD history at wave 4	0.067	0.000
Age at wave 2	~~	Stroke history at wave 4	0.013	0.000
CVD history at wave 2	~~	CVD history at wave 2	1.000	0.000
CVD history at wave 2	~~	Stroke history at wave 2	-0.014	0.000
CVD history at wave 2	~~	Age at wave 3	0.082	0.000
CVD history at wave 2	~~	CVD history at wave 3	0.738	0.000
CVD history at wave 2	~~	Stroke history at wave 3	0.014	0.000
CVD history at wave 2	~~	Age at wave 4	0.053	0.000
CVD history at wave 2	~~	CVD history at wave 4	0.684	0.000
CVD history at wave 2	~~	Stroke history at wave 4	0.000	0.000
Stroke history at wave 2	~~	Stroke history at wave 2	1.000	0.000
Stroke history at wave 2	~~	Age at wave 3	0.107	0.000
Stroke history at wave 2	~~	CVD history at wave 3	-0.027	0.000
Stroke history at wave 2	~~	Stroke history at wave 3	0.719	0.000
Stroke history at wave 2	~~	Age at wave 4	0.104	0.000
Stroke history at wave 2	~~	CVD history at wave 4	-0.039	0.000
Stroke history at wave 2	~~	Stroke history at wave 4	0.643	0.000
Age at wave 3	~~	Age at wave 3	1.000	0.000
Age at wave 3	~~	CVD history at wave 3	0.049	0.000
Age at wave 3	~~	Stroke history at wave 3	0.076	0.000

For peer review only

Age at wave 3	~~	Age at wave 4	0.902	0.000
Age at wave 3	~~	CVD history at wave 4	0.102	0.000
Age at wave 3	~~	Stroke history at wave 4	0.034	0.000
CVD history at wave 3	~~	CVD history at wave 3	1.000	0.000
CVD history at wave 3	~~	Stroke history at wave 3	0.015	0.000
CVD history at wave 3	~~	Age at wave 4	0.032	0.000
CVD history at wave 3	~~	CVD history at wave 4	0.783	0.000
CVD history at wave 3	~~	Stroke history at wave 4	-0.020	0.000
Stroke history at wave 3	~~	Stroke history at wave 3	1.000	0.000
Stroke history at wave 3	~~	Age at wave 4	0.065	0.000
Stroke history at wave 3	~~	CVD history at wave 4	0.012	0.000
Stroke history at wave 3	~~	Stroke history at wave 4	0.807	0.000
Age at wave 4	~~	Age at wave 4	1.000	0.000
Age at wave 4	~~	CVD history at wave 4	0.061	0.000
Age at wave 4	~~	Stroke history at wave 4	0.030	0.000
CVD history at wave 4	~~	CVD history at wave 4	1.000	0.000
CVD history at wave 4	~~	Stroke history at wave 4	-0.039	0.000
Stroke history at wave 4	~~	Stroke history at wave 4	1.000	0.000

LHS = Left-hand side (of the structural equation); RHS = right-hand side. The path column indicates the type of relationship between the two variables, ~ indicates a regression, and ~~ indicates a correlation or covariance.

For peer review only

Supplementary Table 4. Cross-sectional Pearson correlations between cognitive function and blood pressure scores.

Wave	Domain	Adjustment	*r*	95% C.I.		*p*
1	General	None	-0.007	-0.069 —	0.055	0.823
		Points added	-0.048	-0.109 —	0.015	0.133
	Fluid	None	-0.010	-0.072 —	0.053	0.762
		Points added	-0.047	-0.108 —	0.016	0.143
	Speed	None	0.036	-0.026 —	0.098	0.255
		Points added	-0.007	-0.069 —	0.055	0.818
	Memory	None	-0.017	-0.079 —	0.045	0.590
		Points added	-0.055	-0.117 —	0.007	0.083
	Crystallized	None	-0.011	-0.073 —	0.052	0.737
		Points added	-0.043	-0.105 —	0.019	0.173
2	General	None	0.013	-0.056 —	0.082	0.718
		Points added	-0.007	-0.076 —	0.062	0.846
	Fluid	None	-0.004	-0.073 —	0.065	0.901
		Points added	-0.021	-0.090 —	0.048	0.554
	Speed	None	0.024	-0.045 —	0.093	0.494
		Points added	-0.003	-0.072 —	0.065	0.921
	Memory	None	0.014	-0.055 —	0.083	0.696
		Points added	-0.002	-0.071 —	0.067	0.962
	Crystallized	None	0.020	-0.049 —	0.089	0.562
		Points added	0.000	-0.069 —	0.069	0.995
3	General	None	-0.013	-0.092 —	0.067	0.750
		Points added	-0.033	-0.112 —	0.046	0.413
	Fluid	None	-0.001	-0.080 —	0.079	0.982
		Points added	-0.022	-0.101 —	0.058	0.596
	Speed	None	-0.006	-0.086 —	0.073	0.874

For peer review only

		Points added	-0.029	-0.109 —	0.050	0.470
	Memory	None	-0.018	-0.097 —	0.062	0.663
		Points added	-0.037	-0.116 —	0.042	0.360
	Crystallized	None	-0.018	-0.097 —	0.062	0.662
		Points added	-0.026	-0.105 —	0.054	0.530
4	General	None	0.006	-0.088 —	0.101	0.895
		Points added	-0.011	-0.106 —	0.084	0.826
	Fluid	None	-0.005	-0.099 —	0.090	0.925
		Points added	-0.024	-0.118 —	0.071	0.625
	Speed	None	0.007	-0.087 —	0.102	0.878
		Points added	-0.001	-0.096 —	0.094	0.985
	Memory	None	0.022	-0.073 —	0.116	0.655
		Points added	0.007	-0.088 —	0.102	0.887
	Crystal	None	-0.036	-0.131 —	0.059	0.454
		Points added	-0.059	-0.153 —	0.037	0.227

For peer review only

STROBE 2007 (v4) Statement—Checklist of items that should be included in reports of *cohort studies*

Section/Topic	Item #	Recommendation	Reported on page #
**Title and abstract**	1	(*a*) Indicate the study's design with a commonly used term in the title or the abstract	1
		(*b*) Provide in the abstract an informative and balanced summary of what was done and what was found	2,3
**Introduction**			
Background/rationale	2	Explain the scientific background and rationale for the investigation being reported	5, 6
Objectives	3	State specific objectives, including any prespecified hypotheses	6
**Methods**			
Study design	4	Present key elements of study design early in the paper	6, 7
Setting	5	Describe the setting, locations, and relevant dates, including periods of recruitment, exposure, follow-up, and data collection	6, 7
Participants	6	(*a*) Give the eligibility criteria, and the sources and methods of selection of participants. Describe methods of follow-up	6, 7
		(*b*) For matched studies, give matching criteria and number of exposed and unexposed	NA
Variables	7	Clearly define all outcomes, exposures, predictors, potential confounders, and effect modifiers. Give diagnostic criteria, if applicable	7, 8
Data sources/ measurement	8*	For each variable of interest, give sources of data and details of methods of assessment (measurement). Describe comparability of assessment methods if there is more than one group	7, 8
Bias	9	Describe any efforts to address potential sources of bias	10
Study size	10	Explain how the study size was arrived at	6, 7
Quantitative variables	11	Explain how quantitative variables were handled in the analyses. If applicable, describe which groupings were chosen and why	9, 10
Statistical methods	12	(*a*) Describe all statistical methods, including those used to control for confounding	9, 10
		(*b*) Describe any methods used to examine subgroups and interactions	9, 10
		(*c*) Explain how missing data were addressed	10
		(*d*) If applicable, explain how loss to follow-up was addressed	10
		(*e*) Describe any sensitivity analyses	NA
**Results**			

For peer review only

Participants	13*	(a) Report numbers of individuals at each stage of study—eg numbers potentially eligible, examined for eligibility, confirmed eligible, included in the study, completing follow-up, and analysed	Table 1, pg 22
		(b) Give reasons for non-participation at each stage	7
		(c) Consider use of a flow diagram	NA
Descriptive data	14*	(a) Give characteristics of study participants (eg demographic, clinical, social) and information on exposures and potential confounders	Table 1, pg 22
		(b) Indicate number of participants with missing data for each variable of interest	Table 1, pg 22; SMs
		(c) Summarise follow-up time (eg, average and total amount)	7, 11
Outcome data	15*	Report numbers of outcome events or summary measures over time	Table 1, pg 22
Main results	16	(*a*) Give unadjusted estimates and, if applicable, confounder-adjusted estimates and their precision (eg, 95% confidence interval). Make clear which confounders were adjusted for and why they were included	11-14
		(*b*) Report category boundaries when continuous variables were categorized	NA
		(*c*) If relevant, consider translating estimates of relative risk into absolute risk for a meaningful time period	NA
Other analyses	17	Report other analyses done—eg analyses of subgroups and interactions, and sensitivity analyses	13-15
**Discussion**			
Key results	18	Summarise key results with reference to study objectives	15
**Limitations**			
Interpretation	20	Give a cautious overall interpretation of results considering objectives, limitations, multiplicity of analyses, results from similar studies, and other relevant evidence	15, 16
Generalisability	21	Discuss the generalisability (external validity) of the study results	16, 17
**Other information**			
Funding	22	Give the source of funding and the role of the funders for the present study and, if applicable, for the original study on which the present article is based	18

*Give information separately for cases and controls in case-control studies and, if applicable, for exposed and unexposed groups in cohort and cross-sectional studies.

Note: An Explanation and Elaboration article discusses each checklist item and gives methodological background and published examples of transparent reporting. The STROBE checklist is best used in conjunction with this article (freely available on the Web sites of PLoS Medicine at http://www.plosmedicine.org/, Annals of Internal Medicine at http://www.annals.org/, and Epidemiology at http://www.epidem.com/). Information on the STROBE Initiative is available at www.strobe-statement.org.

For peer review only
